# Genome Mining Leads to the Discovery of Kasichelins A–D, Unusual β‑Alanine- and β‑Aminoisobutyric Acid-Containing Siderophores from *Streptomyces*

**DOI:** 10.1021/acs.jnatprod.5c00461

**Published:** 2025-06-25

**Authors:** Marius Bader, Sehee Jang, Patrik Stange, Julian C. Schmid, Stephanie Grond, Chambers C. Hughes, Leonard Kaysser

**Affiliations:** † Department of Pharmaceutical Biology, Pharmaceutical Institute, 9188 University of Tübingen, 72076 Tübingen, Germany; ‡ Interfaculty Institute of Microbiology and Infection Medicine (IMIT), 9188 University of Tübingen, 72076 Tübingen, Germany; § Institute of Organic Chemistry, 9188 University of Tübingen, 72076 Tübingen, Germany; ∥ Cluster of Excellence EXC 2124: Controlling Microbes to Fight Infection, 9188 University of Tübingen, 72076 Tübingen, Germany; ⊥ German Center for Infection Research, Partner Site Tübingen, 72076 Tübingen, Germany; # Department of Pharmaceutical Biology, Institute for Drug Discovery, University of Leipzig, 04109 Leipzig, Germany

## Abstract

Genome mining of a *Streptomyces* strain collected in Tanzania revealed its potential to produce a novel siderophore. Heterologous expression of the corresponding biosynthetic gene cluster (BGC) in *S. coelicolor* M512 resulted in the production of kasichelin A, a new metal-chelating molecule featuring a phenolate oxazoline and a hydroxamate moiety as iron-binding ligands connected via a β-aminoisobutyric acid (BAIBA) linker. Further analysis of the heterologous producer and subsequent optimization of the culture conditions for the wild-type strain led to the isolation of three additional congeners, kasichelins B, C, and D. Marfey’s analysis of kasichelin C, the most abundant congener, established its absolute configuration as 2*R*,7*R*,10*S*,11*R.* Notably, kasichelin C was found to be identical to L-654,040, a partially characterized compound previously described in the patent literature. The iron-binding properties of the kasichelins were evaluated using the chrome azurol S (CAS) assay, UV/vis spectroscopy, and electrospray ionization-mass spectrometry (ESI-MS). All four kasichelins exhibit comparable iron­(III)-binding affinities and surpass deferoxamine in their ability to sequester the metal, despite natural variations in their ligands and linker structures. The biosynthetic origin of the unusual BAIBA moiety presents an intriguing direction for future research.

## Graphical abstract

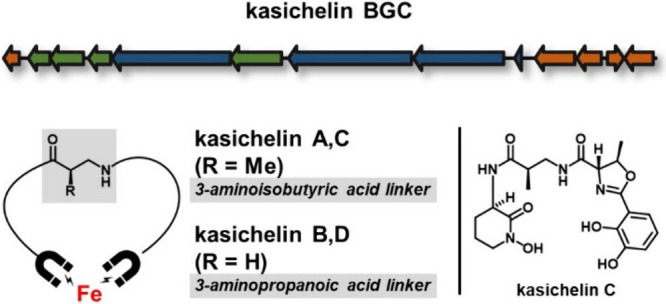

The availability of iron is essential for all living organisms. Many bacteria secure their supply by capturing ferric iron from the environment with small-molecule high-affinity chelators. These “siderophores” are secreted to form stable ferric complexes and are then recognized by specific cell surface receptors that trigger cellular reuptake. In addition to their obvious function in the survival of the individual bacterial cell, siderophores play consequential roles in various physiological and ecological situations. They are, for example, known to shape bacterial communities in the short and long term, triggering coevolution processes and ecological dependencies.^1^ Siderophore-mediated iron acquisition is crucial for the formation of stable biofilms and for the establishment of pathogenic bacteria at the infection site.^2^ Siderophores can also have a range of different bioactivities extending from antimicrobial and cytotoxic; some have even been shown to selectively inhibit the muscarinic M3 receptor.^3,4^ Notably, these molecules have gained increasing attention for their application in the drug development pipeline. One particular field is the use of drug-siderophore conjugates to deliver antibiotics to multiresistant pathogens using a trojan horse strategy.^5^ Other areas include the potential treatment of cancer or malaria.^3^ Thus, the identification and characterization of novel siderophores are highly important from a scientific and pharmaceutical perspective.

Siderophores are often synthesized via nonribosomal peptide synthetase (NRPS) multienzyme assembly lines, in which proteinogenic and nonproteinogenic amino acids are incorporated in a peptidic precursor molecule.^6^ This precursor is further modified by specific NRPS domains or other enzymes to build the final siderophore. One such modification includes the formation of heterocycles, generated e.g. by the cyclization of serine and threonine side chains. Other modifications involve *N*-hydroxylations to produce hydroxamates. The chelating properties of siderophores are typically conferred by the incorporation of multiple bidentate oxygen- or nitrogen-heterocycle-based ligands in a single molecule. Depending on the character of these ligands, one can classify siderophores as hydroxamate-, α-hydroxy carboxylate-, and catecholate and phenolate oxazoline-type siderophores or hybrids thereof. On the basis of these characteristic chemical features, it is possible to match siderophores with their biosynthetic gene clusters (BGCs) with increasing accuracy or, vice versa, to predict the molecular structure of siderophores from orphan BGCs. In order to identify new natural products with interesting bioactivities, we have recently investigated the genus *Nocardia*, mostly known as causative agents of the disease nocardiosis, for their metabolic proficiency.^7^ We found that the pathway for the production of the virulence-conferring nocobactin family of siderophores is widely spread throughout the genus with structurally diverse subtypes.^8^ Thereby, the terpenibactins were discovered and shown to exhibit antimuscarinic activity.^9^ In our bioprospecting campaign we have also focused on bacteria from extreme and unusual environments.^10^ In this regard, first assessments in the early 2000s found a set of strains from the Kilimanjaro region (Tanzania, Mramba Forest, Mwanga District) to be a particularly rich source of chemically interesting molecules.^11,12^ Here, we report genome mining of one of these isolates, *Streptomyces* sp. K17/9, and the discovery of new siderophores, kasichelin A–D. The kasichelins contain phenolate or catecholate oxazoline- and hydroxamate-type ligands linked by rare β-aminobutyric acid (BAIBA) or β-alanine moieties. Furthermore, these compounds were connected to their respective BGC by heterologous pathway expression.

## Results and Discussion

### Genome Mining and Identification of New Siderophores

An actinobacterium strain originally designated as *Actinomyces* sp. K17/9 was isolated from soil samples associated with the unarmed tree *Commiphora eminii*, Burseraceae, in the Kilimanjaro region (Tanzania, Mramba Forest, Mwanga District) among others. Chemical profiling of these strains by Bergere (2000) and Ströch (2003) revealed the isolate K17/9 as a particularly talented producer of secondary metabolites including a number of aromatic compounds, e.g., the benzoxazols UK-1 and piercidin A1.^11–14^ To gain the full picture of the biosynthetic capacity of *Actinomyces* sp. K17/9, we sequenced the genome of the bacterium and subjected it to an automated search for biosynthetic gene clusters (BGCs) using the antiSMASH platform.^15^ The de novo sequencing revealed two contigs with a total genome length of 7,945,601 bp, a GC content of 73%, and 6,953 open reading frames. AutoMLST (Automated Multi-Locus Species Tree) studies were performed to classify strain K17/9.^16^ It was found that the closest homologue in the Genbank sequence database was the bacterium *Streptomyces mobaraensis* DSM 40847 with an average nucleotide identity to K17/9 of 93.3%. Therefore, the strain was reassigned to the genus *Streptomyces* and is now referred to as *Streptomyces* sp. K17/9. The BGC search produced 41 putative pathways with 12 BGCs based on type I polyketide synthases and seven BCGs based on NRPS (Supplemental Figure S1).

One of the NRPS clusters, which encoded putative iron transporter proteins and was annotated as possibly producing a siderophore, particularly caught our attention. This BGC features three genes for NRPS modules: *kasE*, *kasG*, and *kasH* (Figure 1). KasE and KasG are each predicted to contain a condensation (C) domain, an adenylation (A) domain, a peptidyl carrier protein (PCP) and an epimerization (E) domain. In comparison, KasH lacks the E domain and includes a heterocyclization (Cy) domain instead of the regular C domain. Given the direction of the genes and the possible involvement of a single-domain PCP (KasI), the predicted *kas* assembly line would consist of the domain order PCP-Cy-A-PCP-C-A-PCP-E-C-A-PCP-E (Figure 1A). In the KnownClusterBlast, this NRPS machinery revealed highest similarities to enzymes from the gene clusters of amychelin, gobichelin, and cahuitamycin, which are phenolate oxazoline-containing siderophores.^17–19^ This class of natural products also comprises nocobactin NA, vibriobactin, the oxachelins, and the mycobactins, among others. Such compounds have been shown to exhibit diverse bioactivities in various physiological processes, ranging from biofilm inhibition to bacterial virulence.^7–9,19^ Lastly, in silico analysis of the amino acid specificities of the *kas* A domains produced weak to moderate matches for the activation of l-cysteine (A_KasH_), *N*-hydroxy-l-ornithine (A_KasG_), and l-isoleucine (A_KasE_).

**Figure 1:**
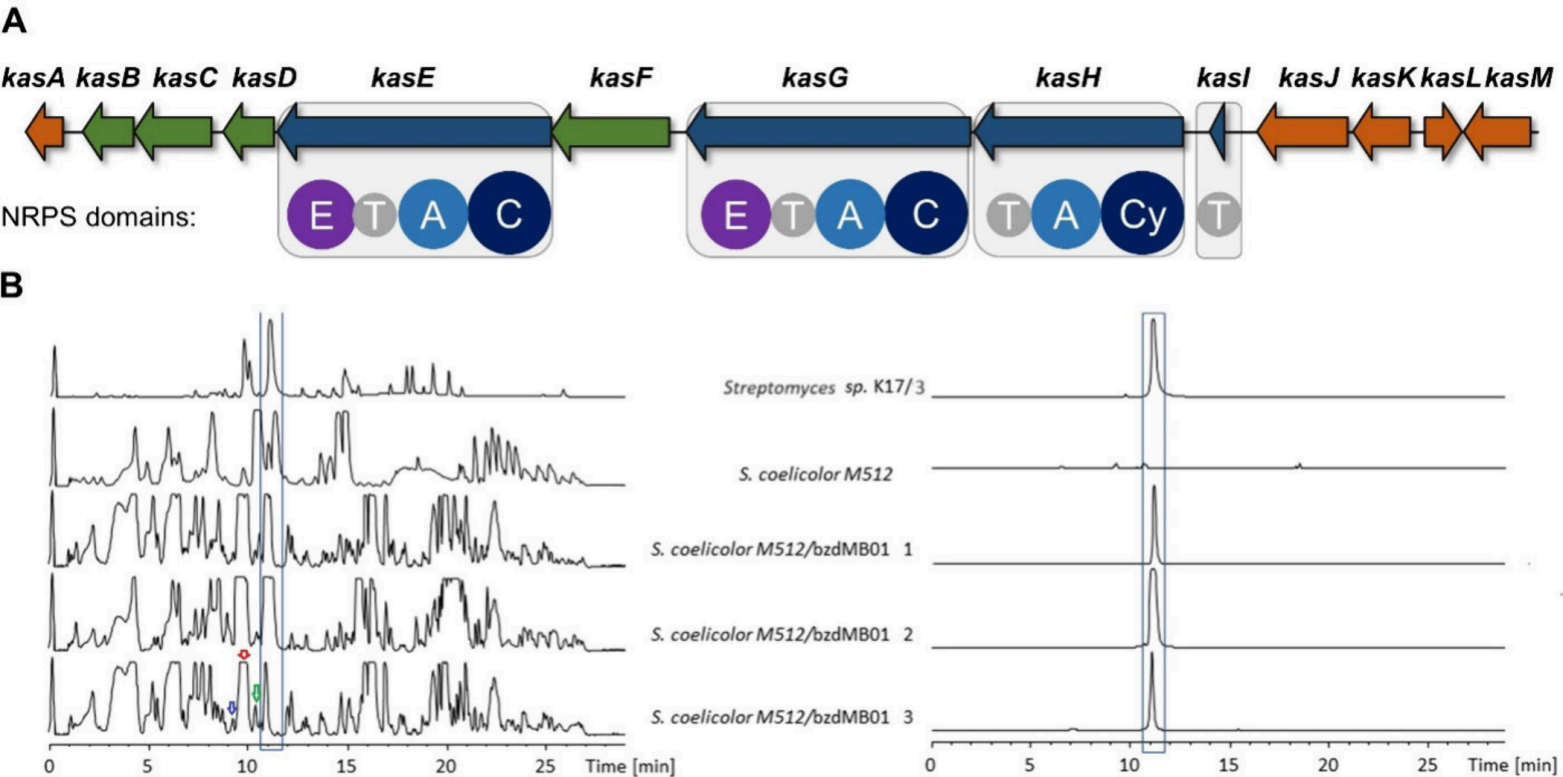
The *kas* biosynthetic gene cluster (BGC) and heterologous production of kasichelin A. A) The *kas* BGC and predicted NRPS domains in KasE, KasG, KasH, and KasI. Blue: NRPS; green: biosynthetic enzymes; brown: transport and regulation. Condensation (C), adenylation (A), cyclization (Cy), epimerization (E), and thiolation (T) domains. B) LC-MS analysis of the heterologous expression of the *kas* BGC in *Streptomyces coelicolor* M512; base peak chromatograms (BPCs) and extracted ion chromatograms (EICs) with *m*/*z* 419.19 for kasichelin A. Peaks representing kasichelin B (green), kasichelin C (red), and kasichelin D (blue) are indicated with colored arrows.

The *kas* BGC encodes additional enzymes that could be involved in secondary metabolite biosynthesis. KasB is a putative SAM-dependent methyltransferase with 39% amino acid sequence identity to NocS2 from the nocathiacin pathway and other *N*-methyltransferases.^20^ KasC shows conserved protein domains found in ATP-grasp enzymes, such as carbamoyl-phosphate synthases, biotin carboxylases, and argininosuccinate lyases. KasD is 80% identical to the arginine dihydrolase Lon22 which generates l-ornithine from l-arginine,^21^ and KasF is a putative dehydrogenase with 69% identity to CdeT, an enzyme that has been associated with the provision of β-alanine in cadaside biosynthesis.^22^ A probable gene cluster would also involve *kasA* and *kasJ,* encoding a putative LuxR-type regulator and a putative transporter of the major facilitator superfamily (MFS), respectively. The gene *kasK* was annotated to encode a putative ViuB-like siderophore interacting protein. Such FAD-binding reductases are thought to facilitate the release of iron from siderophore complexes via the NADPH-dependent reduction of Fe­(III) to Fe­(II), e.g. in *Vibrio cholerae*.^23,24^ From our analysis, we concluded that the *kas* BGC directs the production of a novel siderophore with unusual structural features.

In order to get our hands on the respective molecule, we cloned the gene cluster for heterologous expression. To this end, we constructed a pCC1FOS-based genomic library of *Streptomyces* sp. K17/9 and found fosmid 1D4 to contain the complete putative pathway. We then generated fosmid bzdMB01 by replacing the chloramphenicol resistance gene in the backbone of 1D4 with a restriction cassette. This cassette contained phage-derived machinery for site-specific integration into *Streptomyces* chromosomes. We introduced the *kas* BGC into *Streptomyces coelicolor* M512 and analyzed the resulting mutants for the production of novel compounds by HPLC-MS. Indeed, we could identify a prominent mass signal with *m*/*z* 419.19 in the culture extracts of *Streptomyces* sp. K17/9 and the three investigated heterologous clones (Figure 1B). The MS/MS spectra showed matching fragmentation patterns for *m*/*z* = 419.19 from all four strains. This *m*/*z* 419.19 feature was not observed in the extracts of *S. coelicolor* M512 without the *kas* BGC. High-resolution mass spectrometry gave a monoisotopic mass of *m*/*z* 419.1931 [M + H]^+^ for the heterologous product, which correlated to a molecular formula of C_20_H_26_N_4_O_6_ with a mass error of 0.1 ppm.

Notably, a molecule with the same molecular formula was previously reported from *Streptomyces* sp. K17/9.^11^ Based on misinterpreted preliminary NMR data, it was initially proposed to contain an unusual 2,1-benzisoxazole moiety. However, when we supplemented cultures of *S. coelicolor* M512/bzdMB01 with ^15^N^13^C_4_-l-threonine, we observed a mass shift of +5 Da indicating incorporation of all isotopic labels from the precursor (Supplemental Figure S2–S5). This observation contradicted the originally proposed 2,1-benzisoxazole-containing structure from a biosynthetic perspective and instead pointed to a novel scaffold not previously described in the literature or represented in known databases. A thorough comparative analysis of the metabolic profiles from *S. coelicolor* M512 with and without the *kas* gene cluster as well as the *Streptomyces* sp. K17/9 wild type strain (Figure 1B) revealed three additional compounds which were clearly related in terms of their UV/vis absorption spectra and exact masses (see below). In order to characterize the four kasichelins A–D (**1**–**4**), we prepared an organic extract from a 2 L culture of *Streptomyces* sp. K17/9 and purified the compounds by preparative HPLC. Next, we determined their chemical structures via NMR spectroscopy (Supplemental Figures S6–S29).

### Structure Elucidation of Kasichelins A–D

Kasichelin A (**1**) was isolated as a white solid and found to have the molecular formula C_20_H_26_N_4_O_6_, as deduced from ^1^H and ^13^C NMR data (Table 1; Supplemental Table S1) and HRESIMS (*m*/*z* [M + H]^+^ = 419.1923, calcd for C_20_H_27_N_4_O_6_^+^, 419.1925) (Figure 2). The ^1^H and multiplicity-edited HSQC NMR data in CD_3_OD showed that kasichelin A (**1**) possessed 22 protons bound to carbon, four aromatic protons (δ_H_ 7.67, 7.41, 6.95, 6.89), three heteroatom-bound methine protons (δ_H_ 4.91, 4.50, 4.48), four heteroatom-bound methylene protons (δ_H_ 3.59 (2H), 3.44/3.33), one aliphatic methine proton (δ_H_ 2.63), four aliphatic methylene protons (δ_H_ 2.01/1.77, 2.03–1.94 (2H)), and two methyl groups (δ_H_ 1.54, 1.11). The four protons that did not appear in the ^1^H NMR spectrum were assumed to be exchangeable. The ^13^C NMR spectrum showed four (putative) carbonyl carbons (δ_C_ 177.3, 173.1, 167.8, 166.9), six aromatic carbons (δ_C_ 161.1, 135.0, 129.5, 119.9, 117.7, 111.7), five heteroatom-bound carbons (δ_C_ 80.5, 75.8, 52.5, 51.3, 43.7), three aliphatic carbons (δ_C_ 41.7, 28.6, 21.8), and two methyl groups (δ_C_ 21.3, 15.5).

**Figure 2:**
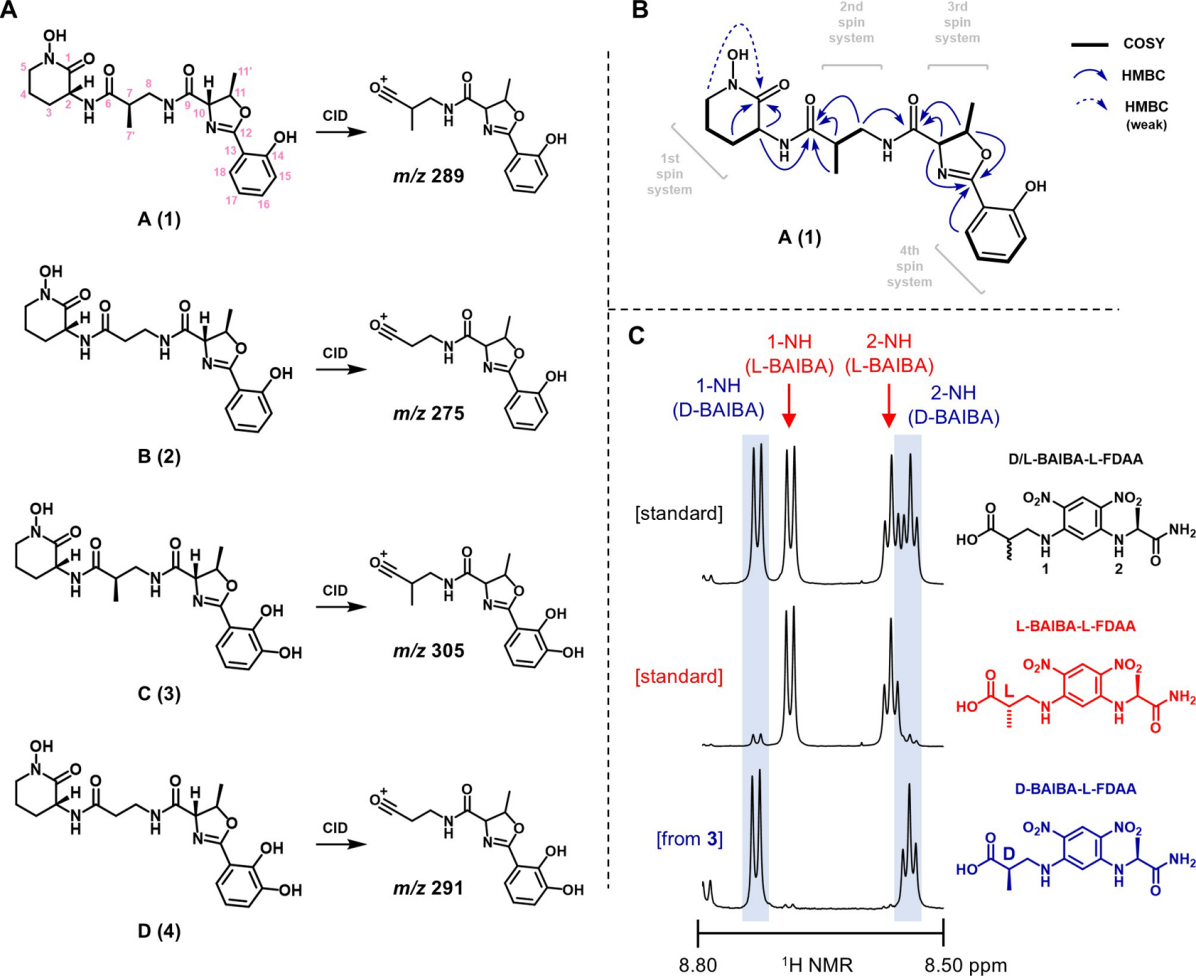
Structure elucidation of kasichelins A–D (**1**–**4**). A) Chemical structures of kasichelins A–D (**1**–**4**). Prominent b_2_-fragments (*m*/*z* 289, 275, 305, and 291) produced upon collision-induced dissociation (CID) of **1**–**4** revealed a common C-terminal residue weighing 130 Da, which corresponded to hydroxyornithine lactam. B) Key COSY and HMBC NMR correlations for **1**. C) ^1^H NMR spectra (δ_H_ 8.80–8.50) for d/l-BAIBA-l-FDAA and l-BAIBA-l-FDAA standards and d-BAIBA-l-FDAA derived from **3**, the most abundant kasichelin.

**Table 1:** ^1^H and ^13^C NMR Data for Kasichelins A–D (**1**–**4**) in CD_3_OD^a^

	kasichelin A (**1**)		kasichelin B (**2**)		kasichelin C (**3**)		kasichelin D (**4**)	
position	δ_C_, type	δ_H_, mult (*J* in Hz)	δ_C_, type	δ_H_, mult (*J* in Hz)	δ_C_, type	δ_H_, mult (*J* in Hz)	δ_C_, type	δ_H_, mult (*J* in Hz)
1	166.9, C	--	166.9, C	--	167.3, C	--	167.3, C	--
2	51.3, CH	4.48, overlap	51.4, CH	4.44, overlap	51.3, CH	4.48, overlap	51.4, CH	4.44, overlap
3a	28.6, CH_2_	2.01, overlap	28.8, CH_2_	1.97, m	28.5, CH_2_	2.01, overlap	28.7, CH_2_	1.97, m
3b		1.77, m		1.68, m		1.77, m		1.67, m
4	21.8, CH_2_	2.03–1.94, m	21.7, CH_2_	1.95–1.88, m	21.8, CH_2_	2.03–1.90, m	21.7, CH_2_	1.95–1.88, m
5	52.5, CH_2_	3.59, overlap	52.5, CH_2_	3.56, overlap	52.4, CH_2_	3.59, overlap	52.5, CH_2_	3.56, overlap
6	177.3, C	--	173.7, C	--	177.3, C	--	173.7, C	--
7	41.7, CH	2.63, m	36.5, CH_2_	2.46, m	41.9, CH	2.63, m	36.5, CH_2_	2.46, m
7’	15.5, CH_3_	1.11, d (7.0)	--	--	15.5, CH_3_	1.11, d (7.0)	--	--
8a	43.7, CH_2_	3.44, dd (13.4,5.2)	37.0, CH_2_	3.56, overlap	43.7, CH_2_	3.44, dd (13.4,5.2)	37.0, CH_2_	3.58, overlap
8b		3.33, overlap		3.52, m		3.33, overlap		3.50, ddd (13.5,6.7,6.7)
9	173.1, C	--	173.0, C	--	173.1, C	--	173.0, C	--
10	75.8, CH	4.50, d (7.5)	75.8, CH	4.46, overlap	75.7, CH	4.49, overlap	75.7, CH	4.46, overlap
11	80.5, CH	4.91, overlap	80.5, CH	4.89, overlap	80.6, CH	4.89, overlap	80.5, CH	4.89, overlap
11’	21.3, CH_3_	1.54, d (6.4)	21.4, CH_3_	1.55, d (6.4)	21.3, CH_3_	1.53, d (6.3)	21.4, CH_3_	1.54, d (6.3)
12	167.8, C	--	167.8, C	--	168.2, C	--	168.2, C	--
13	111.7, C	--	111.7, C	--	111.8, C	--	111.8, C	--
14	161.1, C	--	161.1, C	--	149.5, C	--	149.5, C	--
15	117.7, CH	6.95, dd (8.3,1.1)	117.7, CH	6.96, dd (8.4,1.1)	146.8, C	--	146.8, C	--
16	135.0, CH	7.41, t (7.7)	135.0, CH	7.41, t (7.5)	120.2, CH	6.95, dd (8.0,1.5)	120.3, CH	6.96, dd (8.0, 1.5)
17	119.9, CH	6.89, t (7.7)	120.0, CH	6.90, t (7.5)	119.9^b^, CH	6.74, t (8.0)	119.9, CH	6.75, t (8.0)
18	129.5, CH	7.67, dd (8.0,1.8)	129.5, CH	7.67, dd (8.0,1.7)	119.8^b^, CH	7.17, dd (8.0,1.5)	119.9, CH	7.17, dd (8.0,1.5)

a ^1^H and ^13^C NMR were recorded at 700 and 175 MHz, respectively.

b These signals are exchangeable.

Analysis of the COSY NMR data of **1** revealed four spin systems (see Figure 2). The first spin system consisted of H_1_-2 (δ_H_ 4.48, δ_C_ 51.3), H_2_-3 (δ_H_ 2.01/1.77, δ_C_ 28.6), H_2_-4 (δ_H_ 2.03–1.90, δ_C_ 21.8), H_2_-5 (δ_H_ 3.59, δ_C_ 52.5), and the second spin system extended from H_3_-7’ (δ_H_ 1.11, δ_C_ 15.5) to H_1_-7 (δ_H_ 2.63, δ_C_ 41.7) to H_2_-8 (δ_H_ 3.44/3.33, δ_C_ 43.7). The third spin system extended from H_1_-10 (δ_H_ 4.50, δ_C_ 75.8) to H_1_-11 (δ_H_ 4.91, δ_C_ 80.5) to H_3_-11’ (δ_H_ 1.54, δ_C_ 21.3). The last aromatic spin system extended from H_1_-15 (δ_H_ 6.95, δ_C_ 117.7) to H_1_-16 (δ_H_ 7.41, δ_C_ 135.0) to H_1_-17 (δ_H_ 6.89, δ_C_ 119.9) to H_1_-18 (δ_H_ 7.67, δ_C_ 129.5).

These four partial structures were then connected using HMBC correlations in order to obtain the complete planar structure for **1**. The ornithine lactam was established using HMBC correlations from H_1_-2, H_2_-3, and H_2_-5 to C-1 (δ_C_ 166.9), although the H_2_-5 to C-1 correlation was quite weak. Mutual HMBC correlations from H_1_-2, H_1_-7, H_3_-7’, and H_2_-8 to C-6 (δ_C_ 177.3) connected the first spin system to a β-aminoisobutyric acid (BAIBA) spin system via an amide linkage. This second spin system was connected to the third spin system via HMBC correlations from H_2_-8, H_1_-10, and H_1_-11 to C-9 (δ_C_ 173.1). Connecting the third and fourth spin systems was more difficult due to the presence of several contiguous quaternary carbon atoms and a lone HMBC correlation connecting H_1_-10, H_1_-11, and H_1_-18 to a putative carbonyl carbon at δ_C_ = 168.2. As such, the structure of this portion of kasichelin A (**1**) could not be immediately deciphered.

Kasichelin B (**2**) was isolated as a white solid and found to have the molecular formula C_19_H_24_N_4_O_6_, as deduced from ^1^H and ^13^C NMR data (see Table 1) and HRESIMS (*m*/*z* [M + H]^+^ = 405.1764, calcd for C_19_H_25_N_4_O_6_^+^, 405.1769). The ^1^H and multiplicity-edited HSQC NMR data showed that kasichelin B (**2**) possessed 20 protons bound to carbon. The H_3_-7’ methyl doublet in the second spin system of **1** was conspicuously absent in the ^1^H NMR spectrum of **2**, and the H_1_-7 methine proton (δ_H_ 2.63, δ_C_ 41.7) in **1** was replaced by H_2_-7 methylene protons (δ_H_ 2.46, δ_C_ 36.5) in **2**. COSY correlations between H_2_-7 and H_2_-8 (δ_H_ 3.56/3.52, δ_C_ 37.0) now defined the second spin system. With the remainder of the NMR data being essentially the same as those for **1**, kasichelin B (**2**) was proposed to contain a central β-aminopropanoic acid (β-alanine) moiety.

Kasichelin C (**3**) was isolated as a white solid and found to have the molecular formula C_20_H_26_N_4_O_6_, as deduced from ^1^H and ^13^C NMR data (see Table 1) and HRESIMS (*m*/*z* [M + H]^+^ 435.1872, calcd for C_20_H_27_N_4_O_7_^+^, 435.1874). The ^1^H and multiplicity-edited HSQC NMR data showed that kasichelin C (**3**) possessed 21 protons bound to carbon. The absence of one aromatic doublet of doublets in the ^1^H NMR spectrum of **3** (when compared to **1**) was conspicuous. COSY correlations from H_1_-16 (δ_H_ 6.95, δ_C_ 120.2) to H_1_-17 (δ_H_ 6.74, δ_C_ 119.9) to H_1_-18 (δ_H_ 7.17, δ_C_ 119.8) now defined the fourth spin system. After careful comparison of the NMR data of **3** with NMR data for other known siderophores, such as serratochelin,^25^ vibriobactin,^26^ fluvibactin,^27^ agrobactin,^28^ and fimsbactin,^29^ the catecholate oxazoline structure of **3** became evident. With the remainder of the NMR data essentially the same as that for **1**, the structure of kasichelin C (**3**), except for the placement of a hydroxy group (see below), was established (see Figure 2). Using this insight, the phenolate oxazoline structures of kasichelins A (**1**) and B (**2**) were then revealed.

Kasichelin D (**4**) was isolated as a white solid and found to have the molecular formula C_19_H_24_N_4_O_7_, as deduced from ^1^H and ^13^C NMR data (see Table 1) and HRESIMS (*m*/*z* M+H]^+^ 421.1716, calcd for C_19_H_25_N_4_O_7_^+^, 421.1718). The ^1^H and multiplicity-edited HSQC NMR data showed that kasichelin D (**4**) possessed 19 protons bound to carbon. Both structural variances between kasichelin B (**2**) and **1** and between kasichelin C (**3**) and **1**, the β-alanine and catechol substructures, characterized kasichelin D (**4**). Similar to **2**, COSY correlations between H_2_-7 (δ_H_ 2.46, δ_C_ 36.5) and H_2_-8 (δ_H_ 3.58/3.50, δ_C_ 37.0) defined the β-alanine spin system. Similar to **3**, COSY correlations from H_1_-16 (δ_H_ 6.96, δ_C_ 120.3) to H_1_-17 (δ_H_ 6.75, δ_C_ 119.9) to H_1_-18 (δ_H_ 7.17, δ_C_ 119.9) defined the catechol spin system (see Figure 2).

Having solved and connected all four spin systems in this way, the placement of an additional hydroxyl group was necessary to account for all atoms in the molecular formulas of **1**–**4**. The C-5 chemical shift (δ_C_ 52.4–52.5) and MS/MS analysis of the kasichelins, specifically the prominent b_2_-fragments, suggested a C-terminal *N*-hydroxyornithine lactam residue (Supplemental Figures S30–S33). Hydroxamates are common metal-binding motifs found in a number of siderophores, and *N*-hydroxyornithine lactams, a specific type of hydroxamate, are present in other siderophores like pyoverdine.^30^

At this point, we became aware of a *Streptomyces*-derived natural product called L-654,040 that was described in a patent as having the same planar structure as kasichelin C (**3**).^31^ The full stereostructure of L-654,040 was determined as 2*R*,7*R*,10*S*,11*R* via single crystal X-ray diffraction analysis of crystals grown from a saturated aqueous solution. Unfortunately, ^1^H NMR and optical rotation data for L-650,040 were not described, and the ^13^C NMR data in 10% CD_3_OD/CDCl_3_ for kasichelin C (**3**) were not in perfect agreement with the data reported for L-654,040 in the same solvent. We were thus reluctant to conclude that the two compounds even shared the same relative configuration, and this uncertainty prompted us to undertake a complete configurational analysis of compound **3**. Accordingly, Marfey’s analysis on the acid hydrolysate of **3** was conducted with the goal of establishing the absolute configuration of the molecule. In this way, threonine liberated from hydrolysis of the oxazoline in **3** was determined to have the l-configuration (10*S*,11*R*) (Supplemental Figure S34). Ornithine, which was produced unexpectedly from hydrolysis of **3** via an unknown reductive mechanism, was shown to have the d-configuration (2*R*) (Supplemental Figure S35). Although a previous report describes a small difference in retention time between l-FDAA-l-BAIBA and l-FDAA-d-BAIBA that can be exploited using Gaussian peak fitting,^32^ in our hands the two Marfey’s adducts coeluted, and the configuration of the β-aminoisobutyric acid (BAIBA) subunit from **3** could not be unambiguously determined. For this reason, we developed an NMR-based method for determination of the BAIBA configuration that takes advantage of the fact that several distinct ^1^H NMR signals can be assigned to l-FDAA-l-BAIBA and l-FDAA-d-BAIBA, including the 1-NH amide proton (δ_H(L)_ = 8.69; δ_H(D)_ = 8.73) and 2-NH amide proton (δ_H(L)_ = 8.57; δ_H(D)_ = 8.54) of the Marfey adducts. Indeed, purified l-FDAA-BAIBA from kasichelin C (**3**) showed ^1^H NMR signals that corresponded to those from an l-FDAA-d-BAIBA (7R) standard (Supplemental Figure S36). Thus, kasichelin C (**3**) has the 2*R*,7*R*,10*S*,11*R* configuration and is identical to L-654,040.^31^

### Siderophore Properties of Kasichelins A–D

Since both chemical features and elements of the BGC of kasichelin A–D (**1**–**4**) suggested that the compounds function as siderophores, we set out to determine this empirically (Figure 3). Kasichelin C (**3**), the most abundant kasichelin, was subjected to the standard chrome azurol S (CAS) assay using deferoxamine as a reference (Figure 3B). In the absence of a competing siderophore, the cetrimonium bromide (CTAB)-CAS-Fe­(III) solution exhibited a distinct blue color. Upon titration with kasichelin C, a distinct color change from blue to orange occurred at 40 μM, indicating iron chelation. Compared to deferoxamine (EC_50_ = 26 μM), kasichelin C displayed a lower EC_50_ of 17 μM, suggesting a higher affinity for iron (Supplementary Figure S37). CAS assays for kasichelins A, B, and D produced very similar values, indicating that variations in linker (β-aminobutyric acid vs β-alanine) or ligand structure (phenolate oxazoline vs catecholate oxazoline) have little impact on iron binding (Supplemental Figures S38–S41).

**Figure 3:**
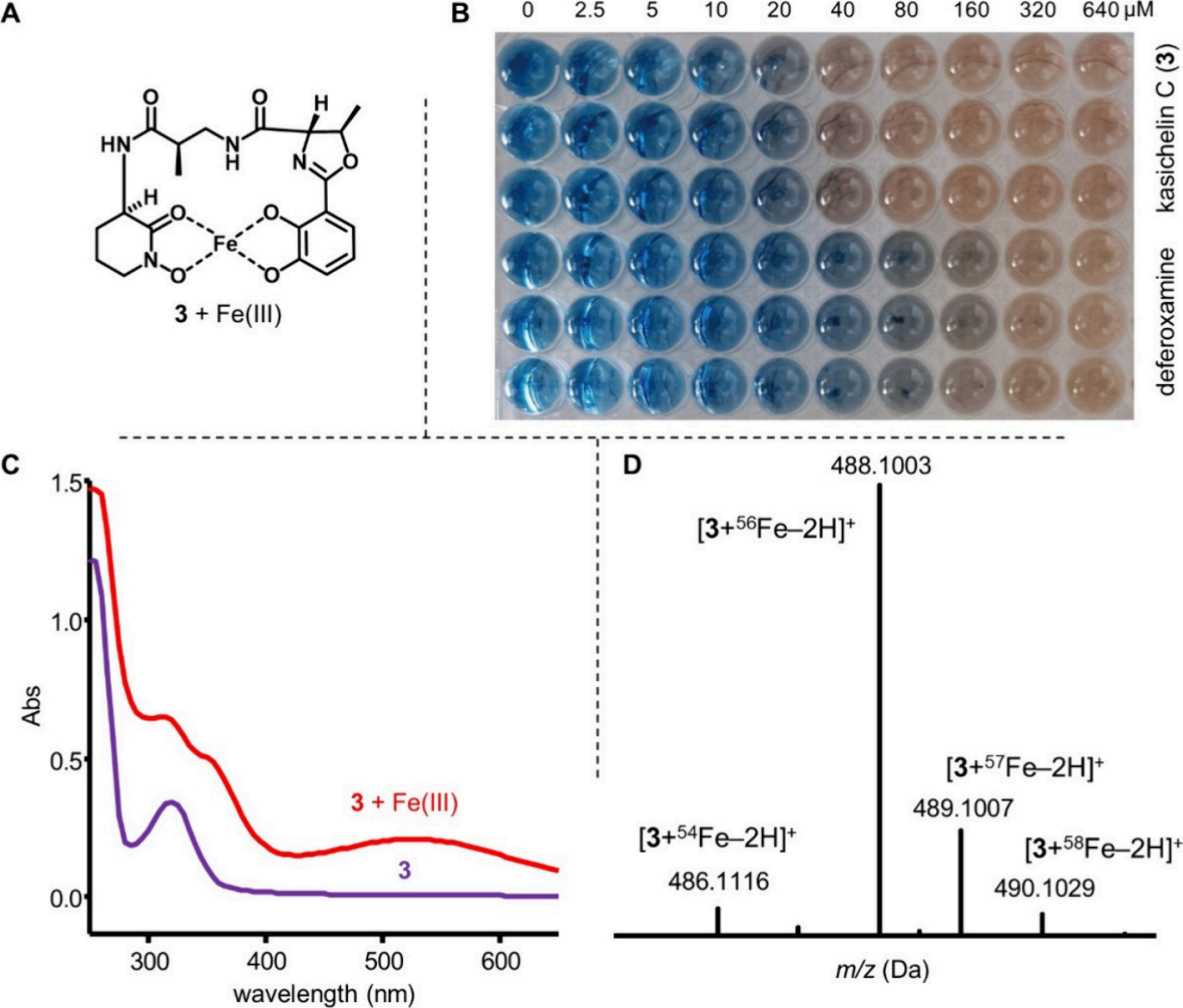
Siderophore properties of kasichelin C (**3**), the most abundant kasichelin. A) Putative structure of the **3**-Fe complex. B) Chrome azurol S (CAS) assay with **3** and deferoxamine as a control. Each well contained a buffered CTAB-CAS-Fe­(III) solution (75 μM, pH 5.6) and final concentrations of siderophore ranging from 0 to 640 μM. The siderophores were tested in triplicate. C) UV/vis spectrum of **3** in MeCN before and after treatment with an aqueous FeCl_3_ solution. D) (+)-ESI-MS spectrum of **3** following treatment with an aqueous FeCl_3_ solution. Very similar results were obtained for kasichelins A, B, and D.

The formation of a stable kasichelin C (**3**)-Fe­(III) complex was further confirmed in orthogonal assays. Treatment of a dilute solution of **3** in MeCN with an unbuffered aqueous solution of FeCl_3_ resulted in a color change from clear to orange. Indeed, the UV/vis spectrum of this treated solution showed the appearance of a long-wavelength absorption band at λ_max_ 500 nm due to ligand-to-metal charge-transfer (Figure 3C). HPLC-(+)­ESI-MS analysis of the same solution confirmed the formation of an Fe­(III) complex that remained stable under reversed-phase chromatographic conditions and exhibited pseudomolecular ions with an isotope pattern (^54^Fe:^56^Fe:^57^Fe:^58^Fe, 6%:92%:2%:0.3%) characteristic of iron coordination (Figure 3D). Similar UV/vis and MS profiles were obtained for kasichelins A, B, and D upon Fe­(III) treatment (Supplemental Figures S42–S43).

To further probe the coordination environment, the kasichelin C–Ga­(III) complex was analyzed by NMR spectroscopy in DMSO-*d*_6_. In the resulting ^1^H NMR spectra, the signals corresponding to the catecholate hydroxyl protons and the *N*-hydroxyornithine lactam proton were no longer observed, consistent with metal coordination. Moreover, changes in the NOESY spectrum indicated a structural reorganization upon metal complexation, particularly within the 5 Å proximity network (Supplemental Table S2).

## Discussion

In this study we report on the discovery of four new siderophores by genome mining, the kasichelins A–D. Catecholates, hydroxamates, and carboxylates are widespread features of siderophores.^8,9^ Although it is a distinct subset of catecholates, the catecholate oxazoline ligand found in the kasichelins is not an infrequent substructure in siderophores. The hydroxamate group in the kasichelins, a *N*-hydroxyornithine lactam, also has precedence among siderophores such as pyoverdine and pseudobactin. What sets the kasichelins apart from other known siderophores is the unusual β-aminobutyric acid (in **1** and **3**) and β-alanine (in **2** and **4**) moieties that join the iron-chelating groups in the molecule. To the best of our knowledge, there are no other siderophores in the literature with a β-aminobutyric acid linker and very few with a β-alanine linker (e.g., madurastatin). The iron-chelating groups of all other known siderophores are joined either by diamino (e.g., fluvibactin, serratochelin), diacid (e.g., desferrioxamine), β-hydroxy acid (e.g., enterobactin), α-amino acid (e.g., ferrichrome), or much more complex (e.g., pyoverdine, yersiniabactin) linkers.

We also confirmed that the *kas* NRPS assembly line is responsible for the production of kasichelins by heterologous expression. The three NRPS modules incorporate three amino acids, l-threonine, β-alanine, and *N*-hydroxy-d-ornithine. However, further details concerning the biosynthesis of the kasichelins can only be speculated about at the current stage. The substrate predictions obtained from the analysis of the *kas* A-domains produced only weak to moderate matches. The antiSMASH Stachelhaus code analysis suggested cysteine as the substrate for A_KasH_, but other algorithms, e.g. SANDPUMA^33^ and NRPSPredictor,^34^ indicated threonine as a putative substrate. Whereas the assignment of isoleucine as a potential substrate for A_KasE_ resulted from a rather weak match of 75%, the antiSMASH Stachelhaus prediction with the highest confidence was for A_KasG_ to activate *N*-hydroxy-l-ornithine. It is thus possible that the kasichelin assembly line functions in the order KasH­(Thr)-KasE­(X)-KasG­(OH-Orn), opposite to the sequence of genes in the BGC (*kasH*-*kasG*-*kasE*). This notion is supported by a manual analysis of BAIBA-specific A-domains from the suguramide, cadaside, and cryptophycin BGCs showing an 80% match of A_KasE_ to A_1_ of SurC (Supplemental Figure S44).^22,35,36^ Analysis of KasH revealed a putative heterocyclization domain, which is likely responsible to generate the oxazoline ring from ACP- or PCP-activated salicylic acid derivatives and l-threonine. The origin of the salicylic acid cannot be explained on the basis of the annotated BGC. However, a similar situation is known from the pathways of other phenolate oxazoline-type siderophores e.g. nocobactin NA.^37^ Here, the enzymes for the synthesis and activation of salicylic acid are encoded at a different locus elsewhere in the genome of the producer strain. In *Streptomyces* sp. K17/9, a putative salicylate synthase, and a putative 2,3-dihydroxybenzoate-AMP ligase can be found in cluster 16 (Supplemental Figure S45). We have assigned this BGC to the production of benzoxazole compound UK-1 that was previously isolated from *Streptomyces* sp. K17/9.^11,38,39^ One could speculate that the required salicylate- and dihydroxybenzoyl-ACPs for kasichelin assembly derives from cluster 16. Indeed, such a biosynthetic and regulatory cross-talk between different siderophore pathways and a separate genetic locus encoding salicylate synthase and a salicylate-AMP ligase has been reported for *Rhodococcus jostii* RHA1.^40^ The NRPS assigned to the production of kasichelins does not contain a thioesterase domain, typically responsible for the release of the peptide from the assembly line. A similar NRPS architecture is observed in the biosynthesis of amychelin, where product release is catalyzed by a stand-alone hydrolase, AmcB.^17^ This enzyme is predicted to use the hydroxylamine group of terminal N-OH-Orn as a nucleophile to generate a cyclic N-OH-Orn moiety. Given the structural and biosynthetic similarities between the kasichelins and amychelins, it is tempting to assume a common release mechanism. However, no AmcB homologue is encoded within the kasichelin BGC. Whether the putative arginine dihydrolase KasD might fulfill a similar role remains purely speculative.

We have further shown that the kasichelin peptides are composed of two d-amino acid residues, one of which is an unusual β-aminoisobutyric acid (BAIBA) amino acid. The presence of both d-amino acids is biosynthetically reflected by the presence of two putative epimerase domains in KasE and KasG. The BAIBA subunit can be found only in very few other bacterial natural products, i.e., the cadasides, cryptophycins, and surugamide F.^22,35,36^ The biosynthesis of this moiety has not been fully clarified in any of these compounds. It has been postulated that the formation of BAIBA follows a catabolic route from thymine, similar to the pyrimidine degradation in animals. The first dedicated step in this pathway involves a dihydropyrimidine aldehyde dehydrogenase.^41^ Intriguingly, KasF is a putative aldehyde dehydrogenase and shows high homology to CdeT from the cadaside BGC and Orf1 from the suguramide BGC with 69% and 63% amino acid sequence identity, respectively. This analysis suggests that the formation of the BAIBA moiety in these compounds indeed starts with the reduction of thymine and is mediated by KasF in the case of kasichelins A and C. Furthermore, the degradation of uracil by the same pathway may generate the β-alanine found in kasichelins B and D. Whether the ATP-grasp enzyme KasC and the hydrolase KasD participate in the subsequent hydrolysis and decarbamoylation reactions to synthesize BAIBA can only be speculated at the current stage. Alternatively, KasC and KasD may function to support the provision of ornithine in the kasichelin assembly process.

The role of putative SAM-dependent methyltransferase KasB in kasichelin biosynthesis remains unclear. Indeed, the role of this putative methyltransferase in kasichelin biosynthesis prompted our interest. Initially, we hypothesized that KasB might contribute to the formation of the BAIBA moiety via the methylation of a β-alanine precursor. However, feeding experiments using l-methionine-[methyl-^13^C] in *Streptomyces* sp. K17/9 did not result in detectable incorporation (Supplemental Figure S46). KasB is a member of the UbiG superfamily, which encompasses numerous SAM-dependent *O*-methyltransferases known to act on phenolic substrates. This raises the possibility that KasB could generate *O*-methylated kasichelin derivatives. Nevertheless, our LC-MS analysis of the culture extracts failed to reveal any credible candidates for such modified compounds (data not shown).

The patent describing the structure of L-654,040 (kasichelin C) includes a thorough description of the antibacterial properties of the siderophore.^31^ Against a variety of Gram-positive and Gram-negative pathogens, including *Staphylococcus aureus*, *Streptococcus faecalis*, *Escherichia coli*, *Klebsiella pneumonia*, and *Pseudomonas aeruginosa*, kasichelin C showed MICs equal to or exceeding 128 μg mL^–1^. Against anaerobic bacteria such as *Clostridium perfringens* and several species of *Bifidobacterium*, kasichelin C likewise showed little antibacterial activity (MIC > 128 μg mL^–1^). These findings suggest that although the kasichelins are chemically unique siderophores, they have limited potential as direct antibacterial agents.

## Experimental Section

### General Experimental Procedures

All reagents and solvents were purchased commercially and were used without further purification. Extracts, fractions, and pure compounds were analyzed on an analytical 1100 Series Agilent Technologies HPLC system coupled to UV/vis (210, 254, and 360 nm) and evaporative light-scattering (ELS) detectors using a Phenomenex Kinetex C18 column (100 × 4.6 mm, 5 μm, 100 Å) with a 10 min gradient from 10–100% MeCN in water containing 0.1% formic acid and a 1.0 mL min^–1^ flow rate. For high-resolution and MS/MS measurements, extracts were analyzed on an analytical 1290 Infinity II Series Agilent Technologies HPLC system coupled to a Bruker Impact II QTOF MS. Chromatography was performed using a Phenomenex Kinetex C18 column (50 × 2.1 mm, 1.7 μm, 100 Å) with a 10 min gradient from 5–100% MeCN in water containing 0.1% formic acid and a 0.5 mL min^–1^ flow rate. Column chromatography was performed on a Teledyne CombiFlash Rf+ Lumen flash chromatography system. Preparative HPLC was performed using a Millipore Waters 600E solvent delivery system with a Phenomenex Kinetex C18 column (250 mm × 21.2 mm, 5 μm, 100 Å) and a 13 mL min^–1^ flow rate. Compounds were detected with a single-wavelength Knauer UV detector at 254 or 360 nm. Infrared (IR) spectra were recorded on a Thermo Nicolet Summit FT-IR. Optical rotations were recorded on an Anton Paar MCP 150 polarimeter. UV/vis spectra were recorded on an Agilent Cary 60 UV/vis spectrophotometer. ^1^H and 2D NMR spectra were recorded on a Bruker 700 MHz spectrometer in CD_3_OD (residual solvent referenced to 3.31 ppm) or DMSO-*d*_6_ (residual solvent referenced to 2.50 ppm). ^13^C NMR spectra were recorded on the same instrument at 175 MHz in CD_3_OD (referenced to 49.0 ppm). The following abbreviations are used to indicate the multiplicity in ^1^H NMR spectra: s = singlet, d = doublet, t = triplet, dd = doublet of doublets, td = triplet of doublets, m = multiplet, and br s = broad singlet.

### Identification and Cultivation of *Streptomyces* sp. K17/9

*Streptomyces* sp. K17/9 was first described as an isolate from soil samples associated with *Commiphora eminii* in the Kilimanjaro region (Tanzania, Mramba Forest, Mwanga District).[11] The samples were collected during several field trips to investigate biodiversity and nutrient dynamics of tropical mountain forests by the Faculty of Forest Sciences and Forest Ecology (Georg-August-Universität Göttingen) in the late 1990s. Nutrient-rich malt-based M2 agar (10 g L^–1^ malt extract, 4 g L^–1^ yeast extract, and 4 g L^–1^ glycose, pH 7.0, with or without 20 g L^–1^ agar) was used for bacterial isolation and maintenance.

### Genome Sequencing, Assembly, and Annotation

High molecular mass genomic DNA was purified from a 100 mL culture of *Streptomyces* sp. K17/9 in tryptic soy broth (TSB) supplemented with 0.5% glycine using the phenol/chloroform extraction method as described by Kieser et al.^42^ DNA quality was assessed by gel electrophoresis, and the quantity was estimated by a fluorescence-based method with the Quant-iT PicoGreen dsDNA kit (Invitrogen). Sequencing of the genomic DNA was provided by Macrogen Inc. using PacBio’s highly accurate long-read sequencing technology. The de novo sequencing and assembly revealed two contigs with a total genome length of 7,945,601 bp at a GC content of 73%, and 6,953 protein coding genes. The draft genome sequence was deposited at NCBI and is available under project number SUB15250581. Annotation of putative biosynthetic gene clusters was conducted with the antiSMASH platform.^15,43^ Genbank files of the kasichelin BGC and the Streptomyces K17/9 genome sequences are provided in the Supporting Information (as files TXT-1 and TXT-2).

### Construction and Screening of Genomic Libraries

For fosmid library construction, the genomic DNA of *Streptomyces* sp. K17/9 was sheared by pipetting to yield approximately 40 kb fragments. The fragmented DNA was cloned into the vector pCCFOS1 using the CopyControl Fosmid Library Production Kit according to the manufacturer’s instructions. The generated genomic library contained 1920 clones. To isolate the *kas* biosynthetic gene cluster, the library was screened with the primer pairs Bzd_S_FW (CTGGACT­GGTACGCC­ATGGG)/Bzd_S_RV (CTGTCCT­CAAACGC­CGGACG) and BZD_E_FW (CGGTGAC­GCTGACCG­ACATC)/Bzd_S_RV (CATCTTGTCCGCTCGCTGCG) to identify the fosmid clone 1D4 containing a 34998 bp insert. Restriction analysis and end-sequencing confirmed that fosmid 1D4 carries the complete putative *kas* cluster. *In silico* analysis was performed with BLAST, antiSMASH and Artemis (Wellcome Trust Genome Campus; Cambridge; UK). The sequence of the *kas* cluster is available at NCBI under accession number SUB15250581. Information on the *kas* gene cluster has also been deposited at the MIBiG database.^8^

### Heterologous Pathway Expression

To allow stable chromosomal integration, the chloramphenicol resistance gene *cat* in fosmid 1D4 was replaced with an integration cassette (int_neo) by λ-Red–mediated recombination to generate fosmid bzdMB01. The int_neo cassette contains an attachment site (attP) and the integrase gene (*int)* of phage ΦC31, a kanamycin resistance gene (*neo*) and an origin of transfer (*ori*T) to allow site-specific integration into most *Streptomyces* chromosomes.^44^ The cassette was obtained as an XbaI restriction fragment from epnLK01, as described previously.^45^ The resulting fosmid bzdMB01 was verified by restriction analysis and transferred into *E. coli* ET12567.^46^ Introduction in *Streptomyces coelicolor* M512 was achieved by triparental intergeneric conjugation with the help of *E. coli* ET12567/pUB307.^47^ Kanamycin resistant exconjugants were selected and designated as *S. coelicolor* M512/bzdMB01 (13).

### Production and Extraction of Kasichelins

For the optimal production of kasichelins A–D (**1**–**4**), the strain was grown in an SM-based medium composed of 2% soy flour, 2% mannitol, 0.5% glycerin, 50 mM l-ornithine (the biosynthetic precursor of **1**–**4**), and 5 mL of 50% (w/v) XAD-16. The pH of the medium was adjusted to 7.2. The preculture was cultivated for 72 h at 30 °C and 100 rpm, and the main production culture (1 L × 5) was then inoculated with 10 mL of preculture per 1 L of medium. After 1 day of cultivation, 5 mL of 10% l-ornithine was added to the culture to further increase production. The strain was cultivated for another 8 days at 30 °C and 100 rpm and then centrifuged at 4,000 rpm for 15 min. The pellet (cells and resin) was washed with water (2 × 5 L), extracted with methanol (2 × 1.5 L), and filtered. The combined organic extract was concentrated under a vacuum to give 708 mg of crude material.

### Purification of kasichelins A–D (**1**–**4**)

A portion of the extract was fractionated via flash chromatography (CombiFlash Rf+ Lumen, 40 g silica gel) using a gradient from 0 to 50% methanol in dichloromethane over 20 min, a 40 mL min^–1^ flow rate, and 254 nm detection. Fractions containing the kasichelins were pooled and concentrated, and the compounds were then purified by preparative HPLC (Phenomenex Kinetex C18, 250 × 21.2 mm, 5 μm, 100 Å) using 45% methanol in water at a 13 mL min^–1^ flow rate and 254 nm detection to yield, according to elution order, kasichelin D (**4**, 13.2 mg, *t*_R_ = 18–20 min), kasichelin C (**3**, 24.8 mg, *t*_R_ = 22–24 min), kasichelin B (**2**, 4.5 mg, *t*_R_ = 26.5–27.5 min), kasichelin A (**1**, 7.3 mg, *t*_R_ = 34–35 min) as white solids.

### Kasichelin A (**1**)

White solid; UV (MeCN) λ_max_ (log ε) 305 (3.67) nm; IR (film): 3292, 2890, 1595 cm^–1^; [α]_D_^24^ +8 (*c* 0.10, MeOH); ^1^H and ^13^C NMR data, see Table 1; HRESIMS *m*/*z* [M + H]^+^ 419.1923, calcd for C_20_H_27_N_4_O_6_^+^, 419.1925.

### Kasichelin B (**2**)

White solid; UV (MeCN) λ_max_ (log ε) 305 (3.71) nm; IR (film): 3280, 2890, 1654 cm^–1^; [α]_D_^24^ +28 (*c* 0.10, MeOH); ^1^H and ^13^C NMR data, see Table 1; HRESIMS *m*/*z* [M + H]^+^ 405.1764, calcd for C_19_H_25_N_4_O_6_^+^, 405.1769.

### Kasichelin C (**3**)

White solid; UV (MeCN) λ_max_ (log ε) 256 (3.98) nm; IR (film): 3299, 2885, 1599, 1497 cm^–1^; [α]_D_^24^–10 (*c* 0.10, MeOH); ^1^H and ^13^C NMR data, see Table 1; HRESIMS *m*/*z* [M + H]^+^ 435.1872, calcd for C_20_H_27_N_4_O_7_^+^, 435.1874.

### Kasichelin D (**4**)

White solid; UV (MeCN) λ_max_ (log ε) 255 (4.04) nm; IR (film): 3300, 2889, 1590, 1499 cm^–1^; [α]_D_^24^–8 (*c* 0.10, MeOH); ^1^H and ^13^C NMR data, see Table 1; HRESIMS *m*/*z* [M + H]^+^ 421.1716, calcd for C_19_H_25_N_4_O_7_^+^, 421.1718.

### Feeding of ^15^N^13^C_4_-l-Threonine and ^13^C_3_-Glycerin

*Streptomyces* sp. K17/9 was cultivated in 50 mL of medium for 24 h and then fed with 1 pulse per hour for 8 h until a final concentration of 5 mM was reached. After 72 h of total fermentation, the cultures were extracted with ethyl acetate (ratio 1:1); the extract was evaporated to dryness and analyzed by LC-DAD-ESI-HR-MS/MS (3 mg mL^–1^ in methanol). Instrument setup: Thermo Scientific UltiMate 3000 UHPLC coupled with Bruker MaXis 4G ESI QTOF, LC Setup: Macherey-Nagel Nucleoshell EC RP-C 18 (150 mm × 2 mm, 2.7 μm), gradient: 10–100% in 20 min at 0.3 mL min^–1^ with methanol containing 0.06% formic acid and water containing 0.1% formic acid. ^15^N^13^C_4_-l-Threonine was fed to a culture in oat bran medium (pH 7.8, 20 g L^–1^ of oat bran with 2.5 mL of trace element solution 11 (3.0 g L^–1^ CaCl_2_·2 H_2_O, 1.0 g L^–1^ Fe­(III) citrate, 220 mg·L^–1^, MnSO_4_·1 H_2_O, 7.3 mg L^–1^,CoCl_2_·6 H_2_O, 25.0 mg L^–1^, CuSO_4_·5 H_2_O, 100 mg L^–1^ ZnCl_2_, 10.0 mg L^–1^, Na_2_MnO_4_·2 H_2_O, 20.0 mg L^–1^ Na_2_B_4_O_7_). ^13^C_3_-Glycerin was fed with 5 mM l-ornithine to a culture in S/M medium (20 g L^–1^ defatted soybean meal, 20 g L^–1^ mannitol).

### Determination of Absolute Configuration

Kasichelin C (2 mg) was hydrolyzed by using 6 N HCl (2 mL) at 115 °C for 1 h. The mixture was allowed to cool and then dried under nitrogen. To remove residual HCl, water (2 mL) was added to the hydrolysate, and the solution was again dried under nitrogen. This procedure was repeated three times. The hydrolysate was dissolved in 1 N NaHCO_3_ (200 μL), and a solution of l-1-fluoro-2,4-dinitrophenyl-5-alanine amide (l-FDAA, 100 μL, 10 mg mL^–1^) in acetone was added. The mixture was heated at 80 °C for 5 min, allowed to cool, quenched with 1 N HCl (200 μL), and then dried under nitrogen. The residue was redissolved in methanol (500 μL), and the solution was analyzed by HPLC-MS (Phenomenex Kinetex C18, 100 × 4.6 mm, 5 μm, 100 Å) using a gradient of 10–60% acetonitrile in water containing 0.1% FA over 50 min at a 1.0 mL min^–1^ flow rate and 360 nm detection. The retention times of l-FDAA standards was used to assign the configuration of kasichelin A: l-FDAA-l-Thr (12.9 min), l-FDAA-d-Thr (16.2 min), l-FDAA-l-allo-Thr (13.1 min), l-FDAA-d-allo-Thr (14.4 min), l-FDAA_2_-l-Orn (26.5 min), and l-FDAA_2_-d-Orn (24.7 min). Since l-FDAA-l-BAIBA and l-FDAA-d-BAIBA coeluted at 19.2 min, the l-FDAA-BAIBA Marfey product from reaction with kasichelin A was purified by preparative HPLC (Phenomenex Kinetex C18, 250 × 21.2 mm, 5 μm, 100 Å) using 25% acetonitrile in water containing 0.1% TFA at a 13 mL min^–1^ flow rate and 360 nm detection) to yield l-FDAA-d-BAIBA (t_R_ = 23.5–24.5 min) as determined by ^1^H NMR spectroscopy (700 MHz, DMSO-*d*_6_): δ 8.98 (s, 1H), 8.73 (d, *J* = 6.6 Hz, 1H), 8.54 (t, *J* = 5.6 Hz, 1H), 7.71 (br s, 1H), 7.41 (br s, 1H), 5.80 (s, 1H), 4.34 (m, 1H), 3.51 (m, 1H), 3.45 (m, 1H), 2.85 (m, 1H), 1.45 (d, *J* = 6.8 Hz, 3H), 1.18 (d, J = 7.1 Hz, 3H)

l-FDAA-d/l-BAIBA: ^1^H NMR (700 MHz, DMSO-*d*_6_): δ 8.99 (s, 1H), δ 8.98 (s, 1H), 8.73 (d, *J* = 6.5 Hz, 1H), 8.69 (d, *J* = 6.5 Hz, 1H), 8.57 (t, *J* = 5.8 Hz, 1H), 8.55 (t, *J* = 5.6 Hz, 1H), 7.72 (br s, 1H), 7.70 (br s, 1H), 7.41 (br s, 2H), 5.80 (s, 1H), 5.79 (s, 1H), 4.34 (m, 1H), 4.31 (m, 1H), 3.56 (m, 1H), 3.52 (m, 1H), 3.45 (m, 1H), 3.43 (m, 1H), 2.85 (m, 1H), 2.81 (m, 1H), 1.46 (d, *J* = 6.8 Hz, 3H), 1.45 (d, *J* = 6.8 Hz, 3H), 1.18 (d, J = 7.1 Hz, 3H), 1.17 (d, J = 7.1 Hz, 3H)

l-FDAA-l-BAIBA: ^1^H NMR (700 MHz, DMSO-*d*_6_): δ 8.98 (s, 1H), 8.69 (d, *J* = 6.5 Hz, 1H), 8.57 (t, *J* = 5.8 Hz, 1H), 7.71 (br s, 1H), 7.41 (br s, 1H), 5.80 (s, 1H), 4.31 (m, 1H), 3.56 (ddd, J = 13.5, 7.9, 5.7 Hz, 1H), 3.43 (dt, *J* = 13.5, 5.7 Hz, 1H), 2.81 (m, 1H), 1.46 (d, *J* = 6.8 Hz, 3H), 1.17 (d, *J* = 7.1 Hz, 3H).

### Chrome Azurol S (CAS) Assay

The iron-binding affinity of the kasichelins was determined using the chrome azurol S assay as described in the literature.^48^ Hexadecyltrimethylammonium bromide (HDTMA) (21.9 mg) was dissolved in water (25 mL) at room temperature. To this solution were added an iron­(III) chloride solution (1.5 mL, 1 mM, prepared by dissolving anhydrous FeCl_3_ in a 10 mM aqueous HCl solution) and an aqueous CAS solution (7.5 mL, 2 mM) at room temperature. 2-(*N*-Morpholino)­ethanesulfonic acid (9.76 g, MES) was diluted in water (50 mL), and a 50% KOH solution was used to adjust the pH of this solution to 5.6. Then, the premade CTAB-CAS-Fe­(III) solution was poured into the MES buffer, and water was added to make 100 mL of the modified CAS assay solution. CTAB-CAS-Fe­(III) with MES buffer solution (100 μL) was added to each well of a 96-well microplate. Stock solutions of kasichelins A–D (12.8 mM, DMSO) were serially diluted in water. Each well was then treated with 100 μL of dilute solutions of the kasichelins (or deferoxamine mesylate) to achieve final concentrations ranging from 640 to 2.5 μM. After incubation at 37 °C for 3 h, the resulting absorbance changes were measured on a microplate reader at 620 nm. Readings were transformed to *A*/*A*_0_ where *A* is the average absorbance for duplicate samples and *A*_0_ is the average absorbance of the associated blanks. The “log­(agonist) vs response” analysis tool from Prism software (Version 10.4.2 for Windows 64-bit; GraphPad Software, Inc.) was used to estimate the EC_50_ values.

### UV/vis Spectroscopic Analysis of Kasichelin-Fe­(III) Complexes

UV/vis spectra of kasichelins A–D were collected from 250 to 650 nm using 2 nm steps with a semimicrocell 10 mm cuvette. Stock solutions of kasichelins A–D (12.8 mM) were prepared by dissolving them in DMSO. A stock solution of iron­(III) was prepared by dissolving anhydrous FeCl_3_ in a 10 mM aqueous HCl solution. Solutions of the kasichelins and iron­(III) were diluted in MeCN (1 mL) to achieve final concentrations of 0.128 mM: apo-**1** UV (MeCN) λ_max_ (log ε) 305 (3.67) nm, halo-**1** UV (MeCN) λ_max_ (log ε) 310 (3.88), 410 (3.26) nm; apo-**2** UV (MeCN) λ_max_ (log ε) 305 (3.71) nm, halo-**2** UV (MeCN) λ_max_ (log ε) 310 (3.86), 409 (3.24) nm; apo-**3** UV (MeCN) λ_max_ (log ε) 256 (3.98), 320 (3.43) nm, halo-**3** UV (MeCN) λ_max_ (log ε) 259 (4.05), 316 (3.71), 354 (3.58), 525 (3.22) nm; apo-**4** UV (MeCN) λ_max_ (log ε) 255 (4.04), 315 (3.43) nm, halo-**4** UV (MeCN) λ_max_ (log ε) 255 (4.08), 356 (3.58), 530 (3.23) nm.

### HRMS Analysis of Kasichelin-Fe­(III) Complexes

To a solution of kasichelin A, B, C, or D (1 mg) in MeCN (1 mL) was added a solution of FeCl_3_ in water (100 μL, 5 mM). The solutions were analyzed by HPLC-(+)­ESI-MS: halo-**1** HRESIMS *m*/*z* [M + ^56^Fe – 2H]^+^ 472.1041, calcd for C_19_H_22_N_4_O_7_^56^Fe^+^, 472.1034; halo-**2** HRESIMS *m*/*z* [M + ^56^Fe – 2H]^+^ 458.0884, calcd for C_20_H_24_N_4_O_7_^56^Fe^+^, 458.0877; halo-**3** HRESIMS *m*/*z* [M + ^56^Fe – 2H]^+^ 488.0986, calcd for C_20_H_24_N_4_O_7_^56^Fe^+^, 488.0983; halo-**4** HRESIMS *m*/*z* [M + ^56^Fe – 2H]^+^ 474.0832, calcd for C_20_H_24_N_4_O_7_^56^Fe^+^, 474.0827.

## Supplementary Material







## Data Availability

The NMR data for kasichelins A–D have been deposited at nmrXiv (www.nmrxiv.org) and can be found at DOI: 10.57992/nmrxiv.p113.

## Notes

^1^H, ^13^C, COSY, TOCSY, HSQC, and HMBC NMR spectra, MS/MS spectra, HPLC chromatograms, and ^1^H NMR spectra from Marfey’s derivatization, CAS assay results, UV/vis spectra, (+)-ESI-MS spectra of iron complexes (PDF)
DNA sequence information as Genbank files (TXT-1 and TXT-2)
