# Supplementary material for: Genome Mining Leads to the Discovery of Kasichelins A–D, Unusual β‑Alanine- and β‑Aminoisobutyric Acid-Containing Siderophores from Streptomyces
Source: J Nat Prod. 2025 Jun 25;88(7):1719–28. doi: 10.1021/acs.jnatprod.5c00461 (PMC12305651; doi:10.1021/acs.jnatprod.5c00461)
Supplement: Supplementary file 1 [file np5c00461_si_001.pdf]

Genome mining leads to the discovery of kasichelins A-D, unusual β-alanine- and β-aminoisobutyric acid-containing siderophores from *Streptomyces*

Marius Bader[1,‡], Sehee Jang[2,‡], Patrik Stange[3,‡], Julian C. Schmid[3], Stephanie Grond[3,*], Chambers C. Hughes[2,4,5,*], Leonard Kaysser[6,*]

[1] Department of Pharmaceutical Biology, Pharmaceutical Institute, University of Tübingen, 72076 Tübingen, Germany

[2] Interfaculty Institute of Microbiology and Infection Medicine (IMIT), University of Tübingen, 72076 Tübingen, Germany

[3] Institute of Organic Chemistry, University of Tübingen, 72076 Tübingen, Germany

[4] Cluster of Excellence EXC 2124: Controlling Microbes to Fight Infection, University of Tübingen, 72076 Tübingen, Germany

[5] German Center for Infection Research, Partner Site Tübingen, 72076 Tübingen, Germany

[6] Department of Pharmaceutical Biology, Institute for Drug Discovery, University of Leipzig, 04109 Leipzig, Germany

[‡] contributed equally

correspondence to

[*] leonard.kaysser@uni-leipzig.de

[*] chambers.hughes@uni-tuebingen.de

[*] stephanie.grond@uni-tuebingen.de

Table of Contents

Figure S1 antiSMASH analysis of the *Streptomyces* sp. K17/9 genome sequence....1
Figure S2 MS/MS analysis of kasichelin A and $^{15}N^{13}C_4$-L-kasichelin A....2
Figure S3 MS/MS analysis of kasichelin B and $^{15}N^{13}C_4$-L-kasichelin B....3
Figure S4 MS/MS analysis of kasichelin C and $^{15}N^{13}C_4$-L-kasichelin C....4
Figure S5 MS/MS analysis of kasichelin D and $^{15}N^{13}C_4$-L-kasichelin D....5
Table S1 NMR spectral data for kasichelin A (**1**) in CD_3OD at 700 MHz....6
Figure S6 ^{1}H NMR (CD_3OD, 700 MHz) of kasichelin A (**1**)....7
Figure S7 ^{13}C NMR (CD_3OD, 700 MHz) of kasichelin A (**1**)....8
Figure S8 COSY NMR (CD_3OD, 700 MHz) of kasichelin A (**1**)....9
Figure S9 TOCSY NMR (CD_3OD, 700 MHz) of kasichelin A (**1**)....10
Figure S10 HSQC NMR (CD_3OD, 700 MHz) of kasichelin A (**1**)....11
Figure S11 HMBC NMR (CD_3OD, 700 MHz) of kasichelin A (**1**)....12
Figure S12 ^{1}H NMR (CD_3OD, 700 MHz) of kasichelin B (**2**)....13
Figure S13 ^{13}C NMR (CD_3OD, 700 MHz) of kasichelin B (**2**)....14
Figure S14 COSY NMR (CD_3OD, 700 MHz) of kasichelin B (**2**)....15
Figure S15 TOCSY NMR (CD_3OD, 700 MHz) of kasichelin B (**2**)....16
Figure S16 HSQC NMR (CD_3OD, 700 MHz) of kasichelin B (**2**)....17
Figure S17 HMBC NMR (CD_3OD, 700 MHz) of kasichelin B (**2**)....18
Figure S18 ^{1}H NMR (CD_3OD, 700 MHz) of kasichelin C (**3**)....19
Figure S19 ^{13}C NMR (CD_3OD, 700 MHz) of kasichelin C (**3**)....20
Figure S20 COSY NMR (CD_3OD, 700 MHz) of kasichelin C (**3**)....21
Figure S21 TOCSY NMR (CD_3OD, 700 MHz) of kasichelin C (**3**)....22
Figure S22 HSQC NMR (CD_3OD, 700 MHz) of kasichelin C (**3**)....23
Figure S23 HMBC NMR (CD_3OD, 700 MHz) of kasichelin C (**3**)....24
Figure S24 ^{1}H NMR (CD_3OD, 700 MHz) of kasichelin D (**4**)....25
Figure S25 ^{13}C NMR (CD_3OD, 700 MHz) of kasichelin D (**4**)....26
Figure S26 COSY NMR (CD_3OD, 700 MHz) of kasichelin D (**4**)....27
Figure S27 TOCSY NMR (CD_3OD, 700 MHz) of kasichelin D (**4**)....28
Figure S28 HSQC NMR (CD_3OD, 700 MHz) of kasichelin D (**4**)....29
Figure S29 HMBC NMR (CD_3OD, 700 MHz) of kasichelin D (**4**)....30
Figure S30 MS/MS fragmentation of kasichelin A (**1**)....31
Figure S31 MS/MS fragmentation of kasichelin B (**2**)....32
Figure S32 MS/MS fragmentation of kasichelin C (**3**)....33

Figure S33 MS/MS fragmentation of kasichelin D (**4**)..34
Figure S34 Marfey's analysis of threonine 35
Figure S35 Marfey's analysis of ornithine..36
Figure S36 ^{1}H NMR (DMSO-d_6, 700 MHz) of L-FDAA-D/L-BAIBA 37
Figure S47 Dose–response curves for the iron binding activity of kasichelin C (**3**)....................................42
Figure S38 Chrome azurol S (CAS) assay of kasichelin A (**1**)...38
Figure S39 Chrome azurol S (CAS) assay of kasichelin B (**2**)...39
Figure S40 Chrome azurol S (CAS) assay of kasichelin C (**3**)...40
Figure S41 Chrome azurol S (CAS) assay of kasichelin D (**4**)...41
Figure S42 UV-visible spectra of kasichelins A-D (**1**-**4**) and Fe(III)-kasichelin A-D complexes 43
Figure S43 MS spectra of Fe(III)-kasichelin A-D complexes 44
Table S2 ^{1}H and ^{13}C NMR data for kasichelin C (**3**) with Ga(III) and $^{13}C_3$-labeled compound 45
Figure S44 Analysis of the kasichelin A domains...46
Figure S45 Genetic organization of cluster 16 47
Figure S46 LC-MS analysis of L-methionine-[methyl-^{13}C] feeding……………………………………………...48

antiSMASH antibiotics & Secondary Metabolite Analysis SHell
Version 4.0.2

Select Gene Cluster:
Overview 1 2 3 4 5 6 7 8 9 10 11 12 13 14 15 16 17 18 19 20 21 22 23 24 25 26 27 28 29 30 31 32 33 34 35 36 37 38 39 40 41

Identified secondary metabolite clusters

Cluster	Type	From	To	Most similar known cluster	MIBiG BGC-I
The following clusters are from record MB02_1_1:					
Cluster 1	T1pks	51070	98695	-	-
Cluster 2	Thiopeptide-T3pks-Lantipeptide-T1pks	104649	251187	Cyclothiazomycin_B_biosynthetic_gene_cluster (100% of genes show similarity)	BGC0001145_
Cluster 3	Terpene-Nrps	252307	338990	Herbimycin_biosynthetic_gene_cluster (10% of genes show similarity)	BGC0000074_
Cluster 4	Terpene	392963	413883	-	-
Cluster 5	T2pks	462665	505153	Simocyclinone_D9_/_simocyclinone_D10_/_simocyclinone_D11 (33% of genes show similarity)	BGC0001308_
Cluster 6	Bacteriocin	509295	520215	-	-
Cluster 7	T1pks	520420	567826	Filipin_biosynthetic_gene_cluster (15% of genes show similarity)	BGC0000059_
Cluster 8	T1pks-Terpene	621091	693252	Cinnamycin_biosynthetic_gene_cluster (14% of genes show similarity)	BGC0000503_
Cluster 9	Nrps	730901	774364	Marformycins_biosynthetic_gene_cluster (12% of genes show similarity)	BGC0001214_
Cluster 10	Terpene	842271	863287	-	-
Cluster 11	Lantipeptide-T1pks-Nrps	902392	1018304	Cahuitamycins_biosynthetic_gene_cluster (25% of genes show similarity)	BGC0001351_
Cluster 12	Terpene	1020043	1042121	-	-
Cluster 13	T1pks-Otherks	1065333	1119326	Dutomycin_biosynthetic_gene_cluster (4% of genes show similarity)	BGC0001409_
Cluster 14	T1pks-T3pks-Terpene	1206157	1298894	Furaquinocin_A_biosynthetic_gene_cluster (26% of genes show similarity)	BGC0001078_
Cluster 15	Indole-Terpene	1300418	1402073	Hopene_biosynthetic_gene_cluster (76% of genes show similarity)	BGC0000663_
Cluster 16	Otherks-Nrps	1454651	1528795	Nataxazole_biosynthetic_gene_cluster (48% of genes show similarity)	BGC0001213_
Cluster 17	Terpene	1540643	1562055	Kanamycin_biosynthetic_gene_cluster (53% of genes show similarity)	BGC0000706_
Cluster 18	Otherks-T1pks-Siderophore	1601680	1670751	Grincamycin_biosynthetic_gene_cluster (5% of genes show similarity)	BGC0000229_
Cluster 19	T1pks	1678537	1722439	Meilingmycin_biosynthetic_gene_cluster (3% of genes show similarity)	BGC0000093_
Cluster 20	Ladderane	1885675	1928086	Metatricycloene_biosynthetic_gene_cluster (9% of genes show similarity)	BGC0001369_
Cluster 21	Siderophore	2029042	2040829	-	-
Cluster 22	T3pks	2425988	2467100	Siomycin_biosynthetic_gene_cluster (7% of genes show similarity)	BGC0000611_
Cluster 23	Lantipeptide	2789480	2815291	Meilingmycin_biosynthetic_gene_cluster (2% of genes show similarity)	BGC0000093_
Cluster 24	T3pks-Siderophore	3458904	3507938	Herboxidiene_biosynthetic_gene_cluster (2% of genes show similarity)	BGC0001065_
Cluster 25	T1pks	4595174	4639208	Siomycin_biosynthetic_gene_cluster (7% of genes show similarity)	BGC0000655_
Cluster 26	T2pks	4949997	4992545	Aranciamycin_biosynthetic_gene_cluster (78% of genes show similarity)	BGC0000197_
Cluster 27	Terpene	5457088	5478386	Lomofungin_biosynthetic_gene_cluster (13% of genes show similarity)	BGC0001302_
Cluster 28	T3pks	5676690	5717838	-	-
Cluster 29	Lassopeptide	5723609	5746206	-	-
Cluster 30	Other	5936603	5977391	Albonoursin_biosynthetic_gene_cluster (50% of genes show similarity)	BGC0000851_
Cluster 31	Siderophore	6127810	6139471	-	-
Cluster 32	T1pks	6169721	6218683	Lipomycin_biosynthetic_gene_cluster (22% of genes show similarity)	BGC0001003_
Cluster 33	Terpene	6411056	6432075	Streptomycin_biosynthetic_gene_cluster (2% of genes show similarity)	BGC0000717_
Cluster 34	Terpene	6685172	6706410	-	-
Cluster 35	T1pks	6751316	6796670	Macrotetrolide_biosynthetic_gene_cluster (33% of genes show similarity)	BGC0000244_
Cluster 36	T1pks-Nrps	6856323	7062129	Piericidin_A1_biosynthetic_gene_cluster (100% of genes show similarity)	BGC0001169_
Cluster 37	Other	7181461	7222834	A-503083_biosynthetic_gene_cluster (7% of genes show similarity)	BGC0000288_
Cluster 38	Arylpolyene	7256870	7297997	Hygromycin_A_biosynthetic_gene_cluster (75% of genes show similarity)	BGC0000698_
Cluster 39	Fused-Terpene	7330808	7370932	Leupyrrin_biosynthetic_gene_cluster (5% of genes show similarity)	BGC0000380_
Cluster 40	Nrps	7489305	7597582	Streptomycin_biosynthetic_gene_cluster (6% of genes show similarity)	BGC0000717_
Cluster 41	T1pks-Nrps	7657811	7723523	Pyridomycin_biosynthetic_gene_cluster (19% of genes show similarity)	BGC0001039_

Figure S1 Overview of the antiSMASH analysis of the *Streptomyces* sp. K17/9 genome sequence.

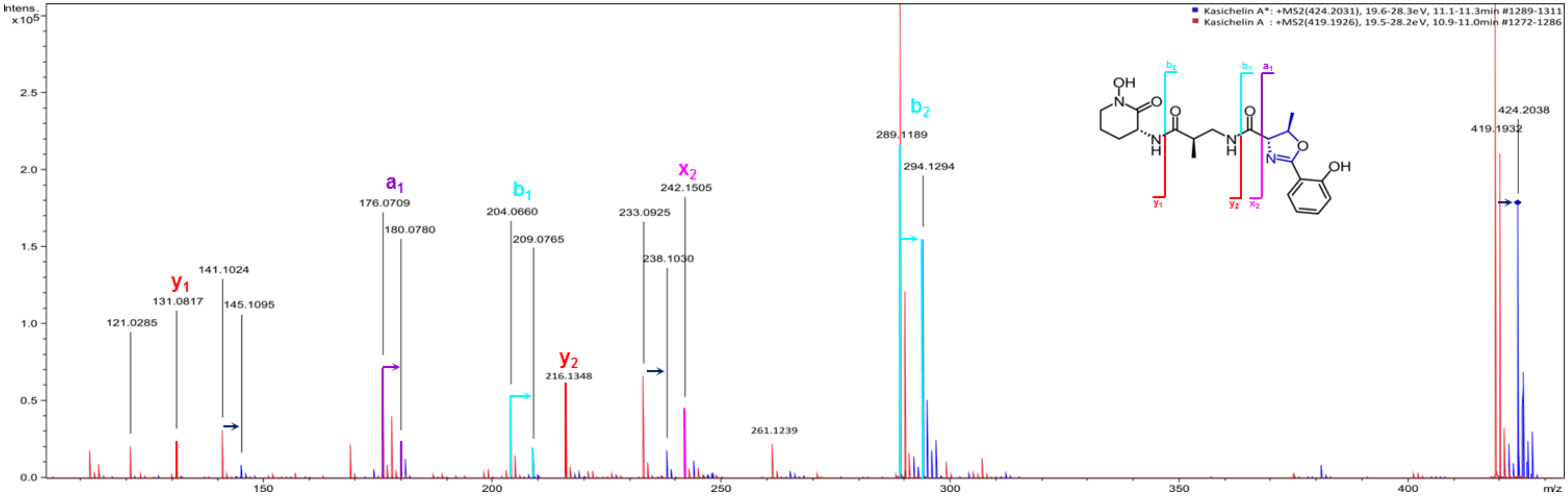

Figure S2 MS/MS analysis of kasichelin A (**1**, HRESIMS *m/z* $[M+H]^+$ 419.1932, calcd for $C_{20}H_{27}N_4O_6$, 419.1925) vs MS/MS pattern of incorporated $^{15}N^{13}C_4$-L-threonine kasichelin A (HRESIMS *m/z* $[M+H]^+$ 424.2038, calcd for $^{13}C_4C_{16}H_{27}{}^{15}N_1N_3O_6$, 424.2029).

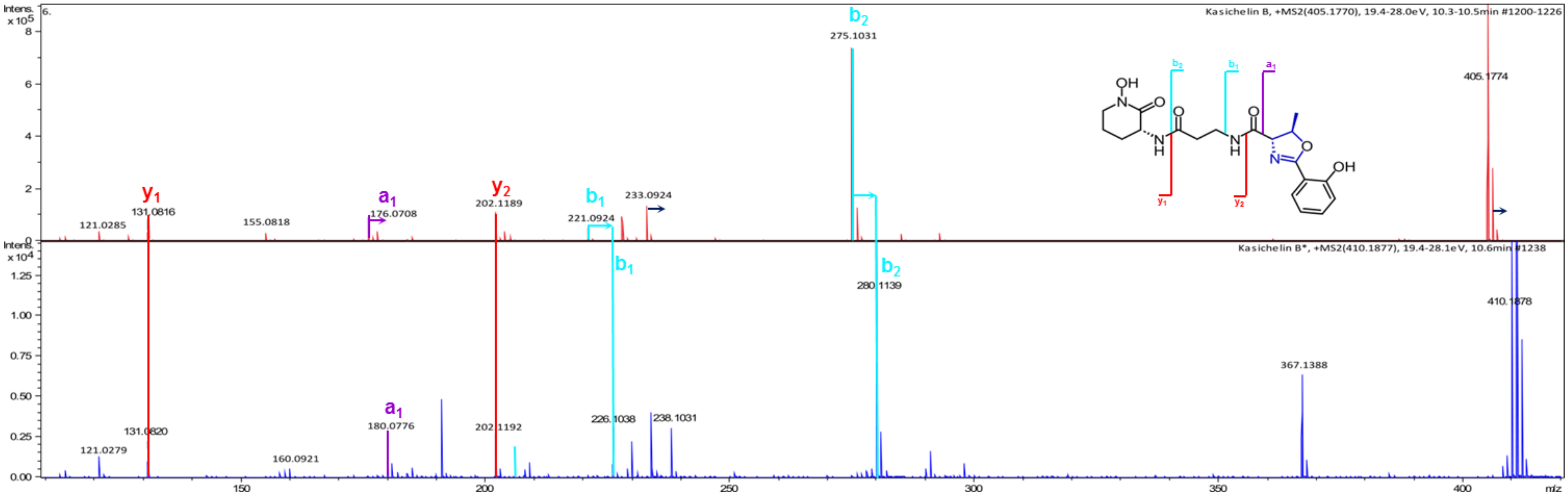

Figure S3 MS/MS analysis of kasichelin B (**2**, HRESIMS *m/z* $[M+H]^+$ 405.1774, calcd for $C_{19}H_{25}N_4O_6$, 405.1769) vs MS/MS pattern of incorporated $^{15}N^{13}C_4$-L-threonine kasichelin B (HRESIMS *m/z* $[M+H]^+$ 410.1878, calcd for $^{13}C_4C_{15}H_{25}{}^{15}N_1N_3O_6$, 410.1873).

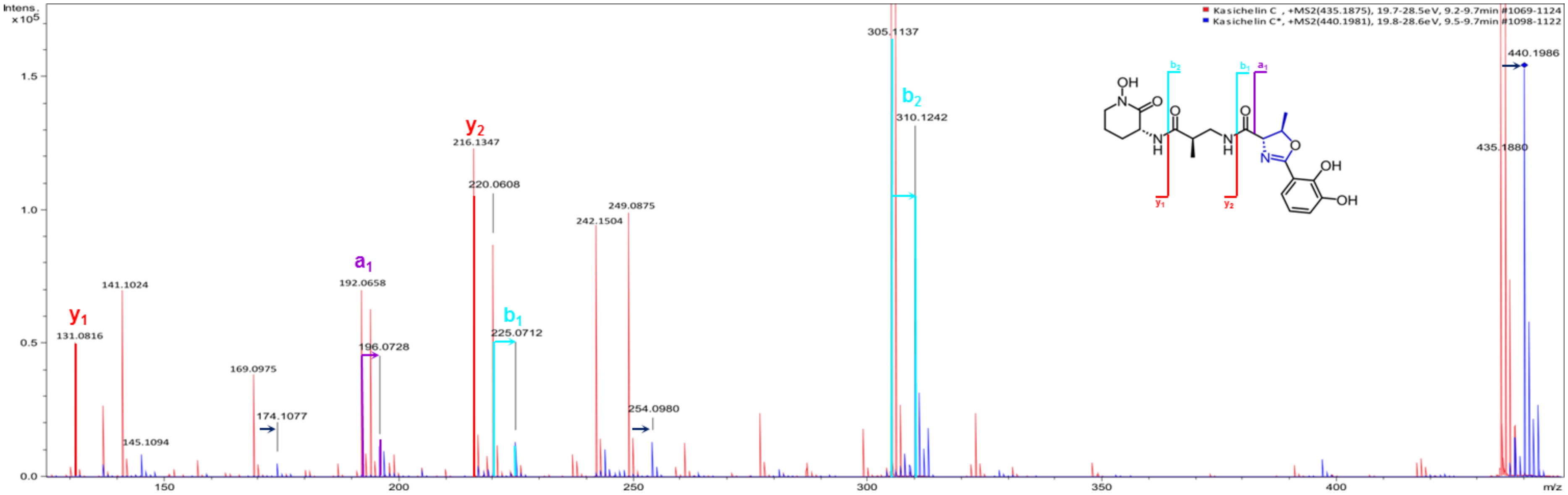

Figure S4 MS/MS analysis of kasichelin C (**3**, HRESIMS *m/z* [M+H]$^+$ 435.1880, calcd for $C_{20}H_{27}N_4O_7$, 435.1874) vs MS/MS pattern of incorporated $^{15}N^{13}C_4$-L-threonine kasichelin C (HRESIMS *m/z* [M+H]$^+$ 440.1986, calcd for $^{13}C_4C_{16}H_{27}{}^{15}N_1N_3O_7$, 440.1979).

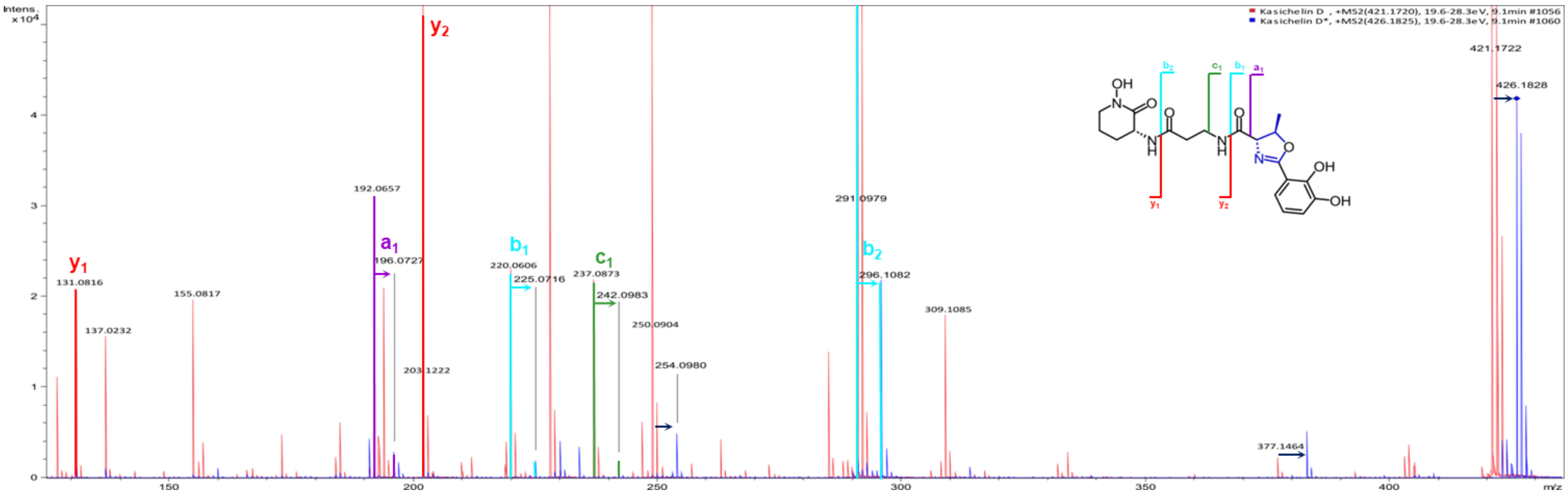

Figure S5 MS/MS analysis of kasichelin D (**4**, HRESIMS *m/z* M+H]$^+$ 421.1722, calcd for $C_{19}H_{25}N_4O_7$, 421.1718) vs MS/MS pattern of incorporated $^{15}N^{13}C_4$-L-threonine kasichelin D (HRESIMS *m/z* [M+H]$^+$ 426.1828, calcd for $^{13}C_4C_{16}H_{25}{}^{15}N_1N_3O_7$, 4260.1822).

Table S1. NMR spectral data for kasichelin A (**1**) in CD_3OD at 700 MHz

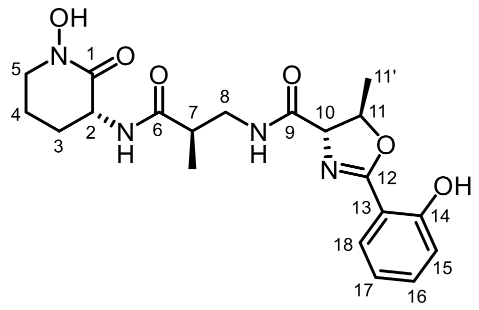

carbon #	δ_C	δ_H, mult (*J* in Hz)	COSY	HMBC
1	166.9	--	--	--
2	51.3	4.48, overlap	3a,3b	1,3,6
3a<br>3b	28.6	2.01, overlap<br>1.77, m	3b<br>3a	2,4<br>2,4
4	21.8	2.03-1.94, m	5	3
5	52.5	3.59, overlap	4	1,3,4
6	177.3	--	--	--
7	41.7	2.63, m	8	6,8
7'	15.5	1.11, d (7.0)	--	--
8a<br>8b	43.7	3.44, dd (13.4,5.2)<br>3.33, overlap	7<br>7	6,7<br>6,7
9	173.1	--	--	--
10	75.8	4.50, d (7.5)	11	9,12
11	80.5	4.91, overlap	10,11'	9,11,11',12
11'	21.3	1.54, d (6.4)	11	10,11
12	167.8	--	--	--
13	111.7	--	--	--
14	161.1	--	--	--
15	117.7	6.95, dd (8.3,1.1)	16	--
16	135.0	7.41, t (7.7)	15,17	14,15,17
17	119.9	6.89, t (7.7)	16,18	13,15
18	129.5	7.67, dd (8.0,1.8)	17	12,14,17

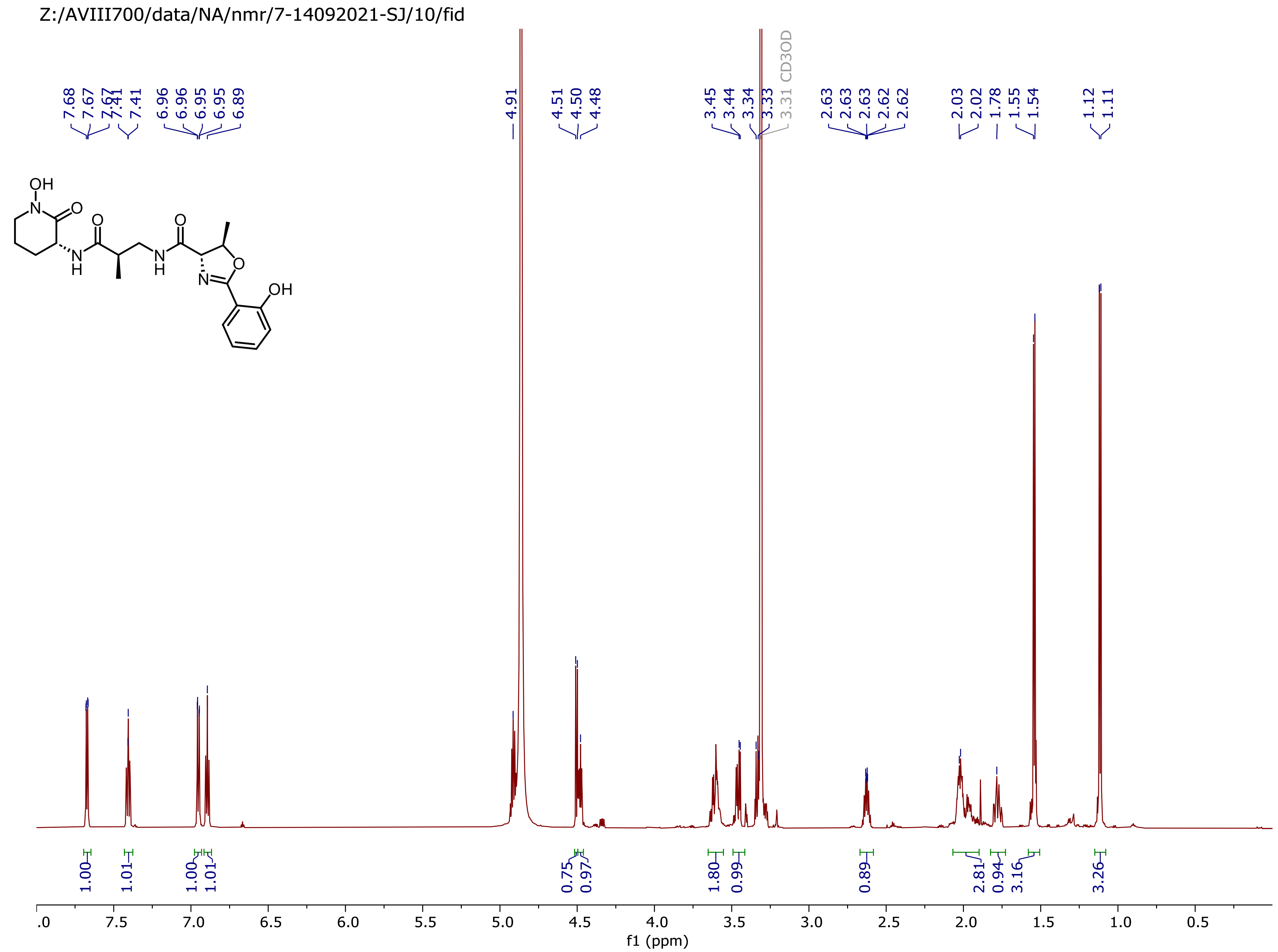

Figure S6 ^{1}H NMR (CD_3OD, 700 MHz) of kasichelin A (**1**)

Z:/AVIII700/data/NA/nmr/7-14092021-SJ/11/fid

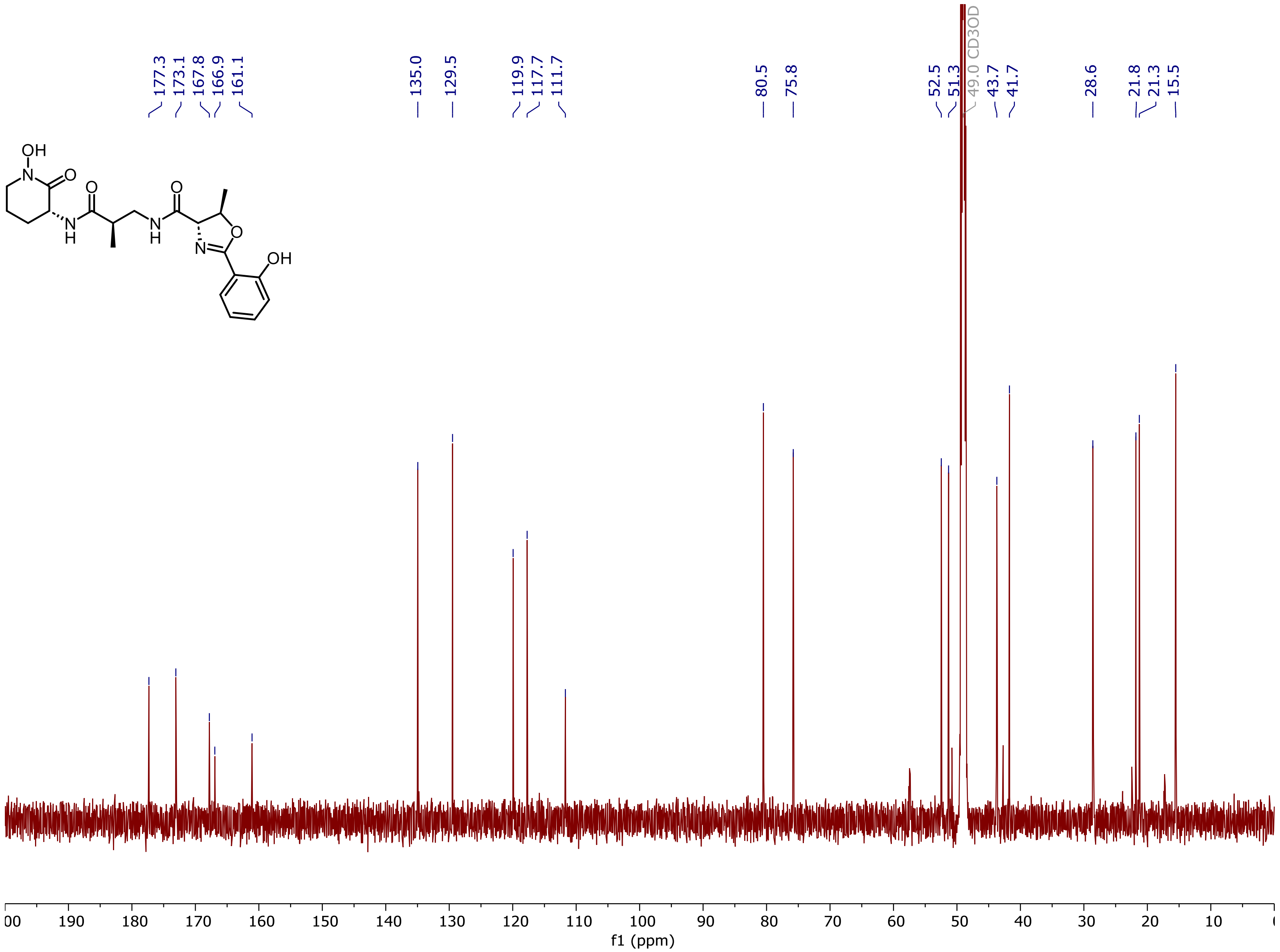

Figure S7 ^{13}C NMR (CD_3OD, 700 MHz) of kasichelin A (**1**)

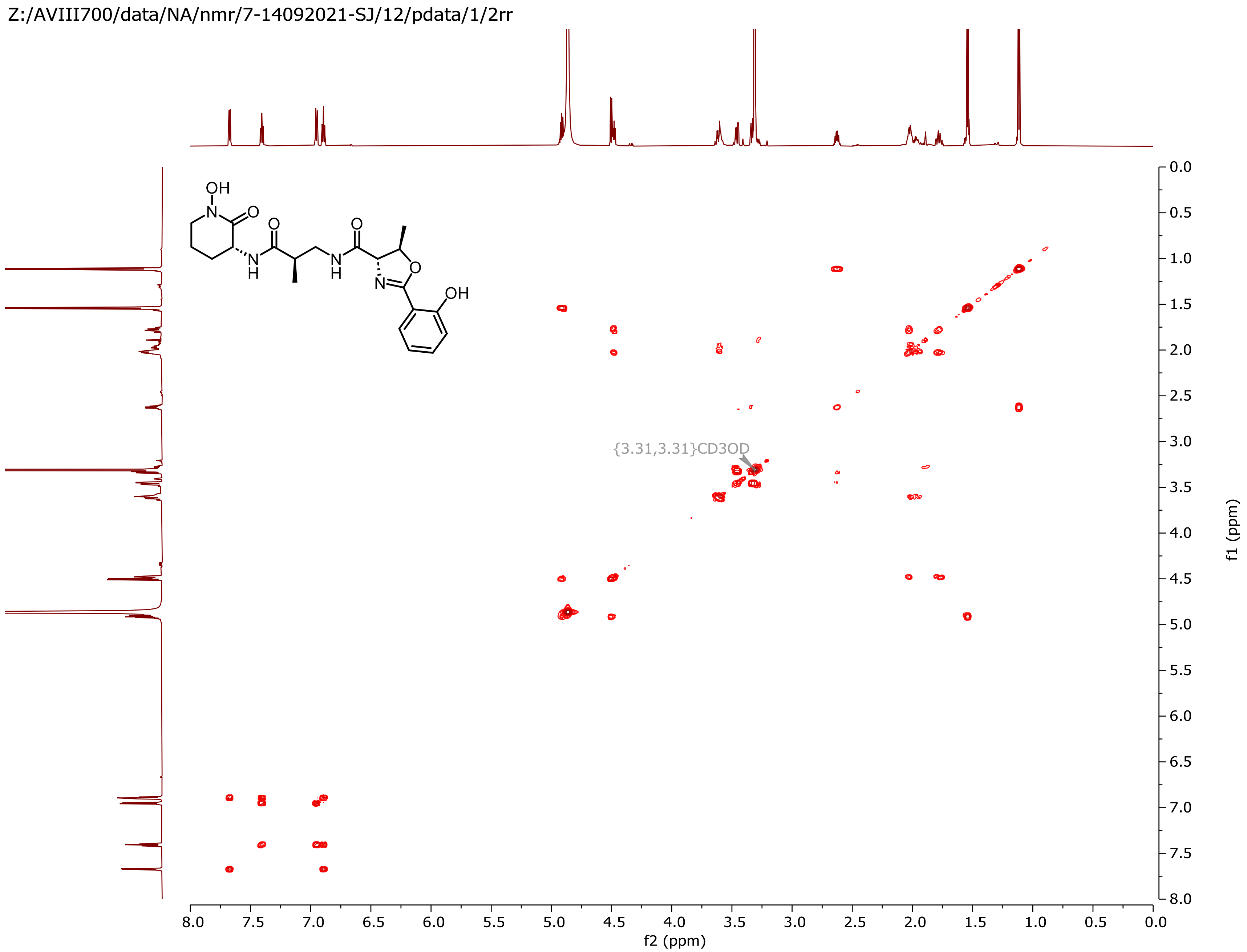

Figure S8 COSY NMR (CD_3OD, 700 MHz) of kasichelin A (**1**)

Z:/AVIII700/data/NA/nmr/7-14092021-SJ/13/ser

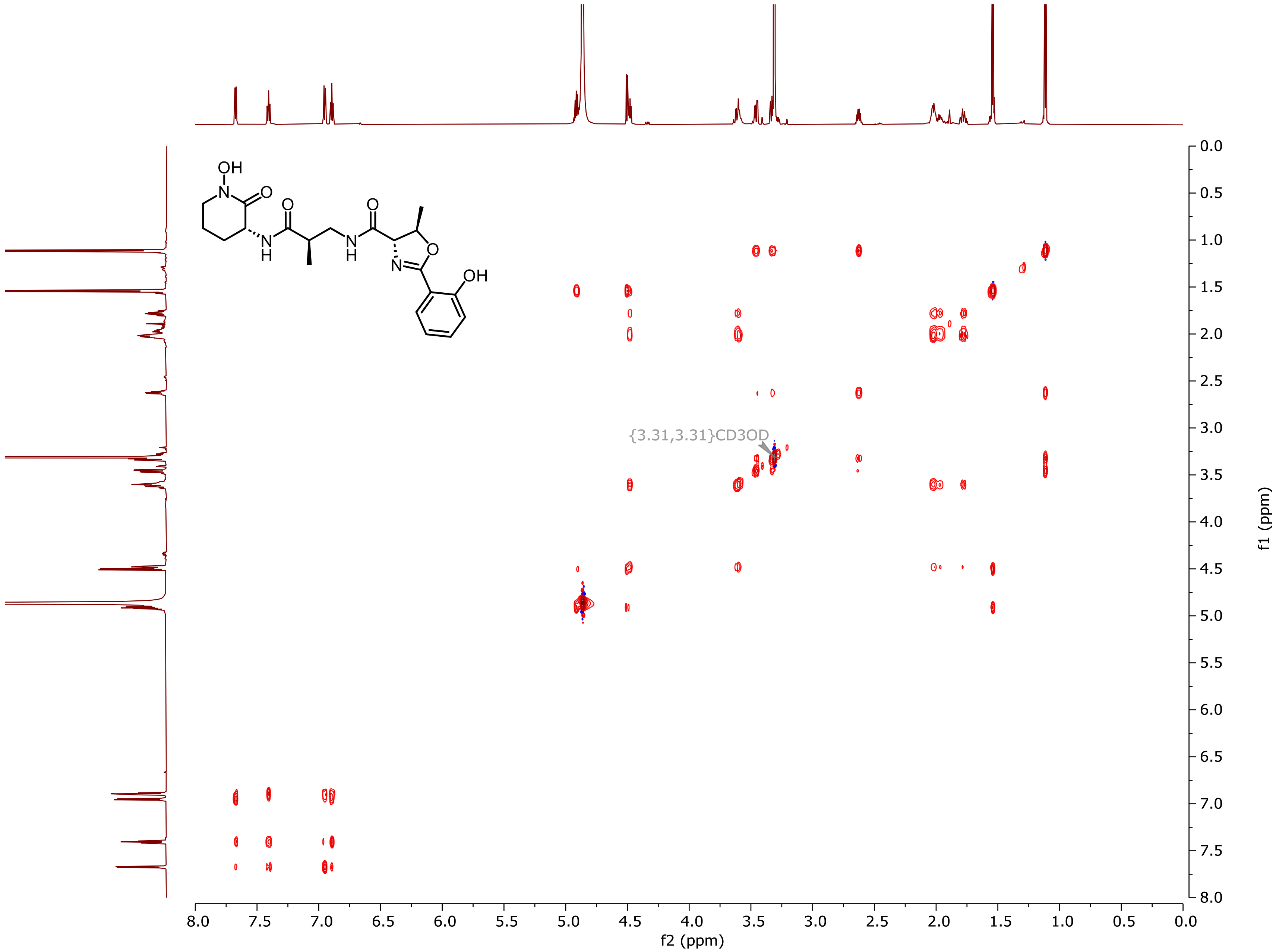

Figure S9 TOCSY NMR (CD_3OD, 700 MHz) of kasichelin A (**1**)

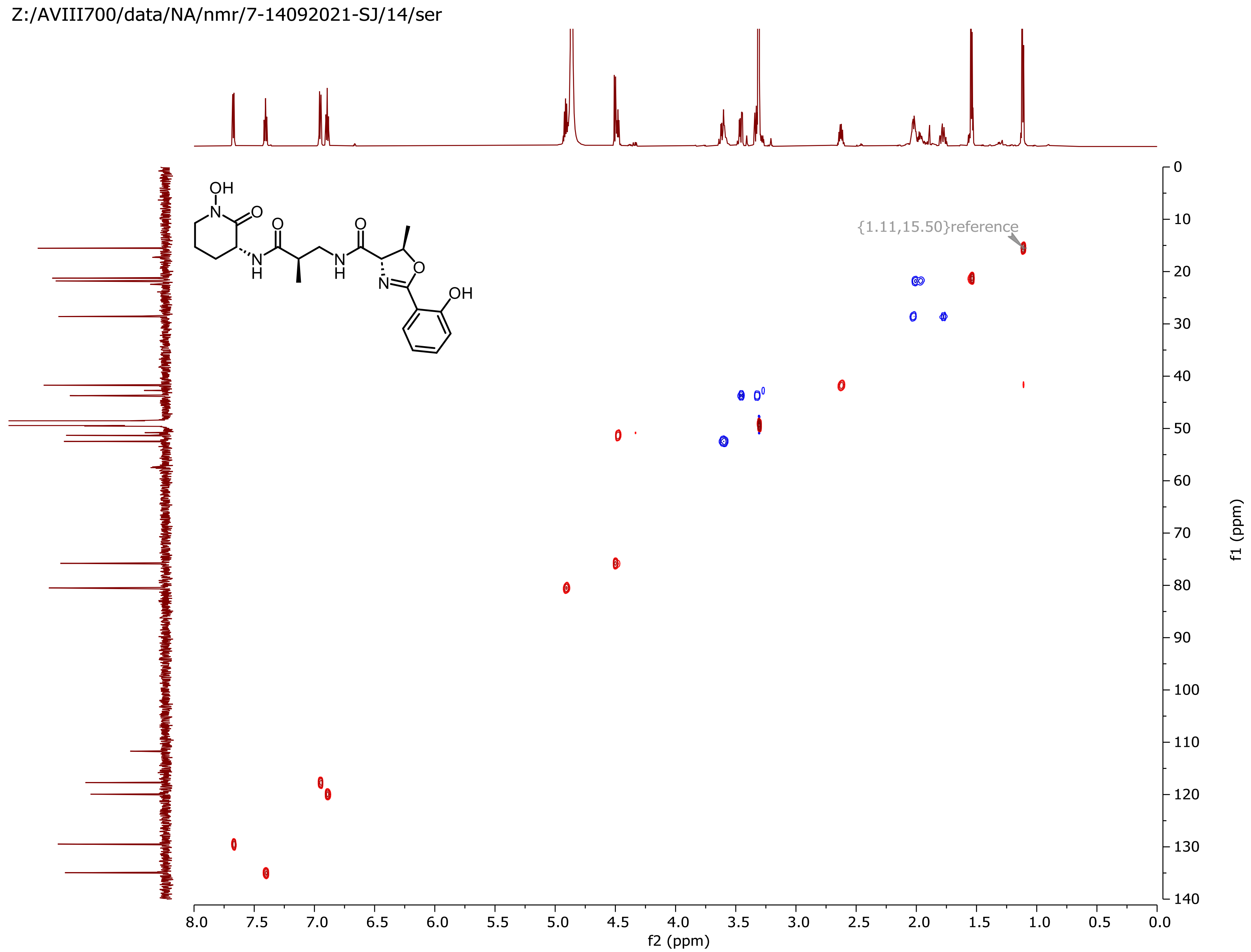

Figure S10 HSQC NMR (CD_3OD, 700 MHz) of kasichelin A (**1**)

Z:/AVIII700/data/NA/nmr/7-14092021-SJ/15/pdata/1/2rr

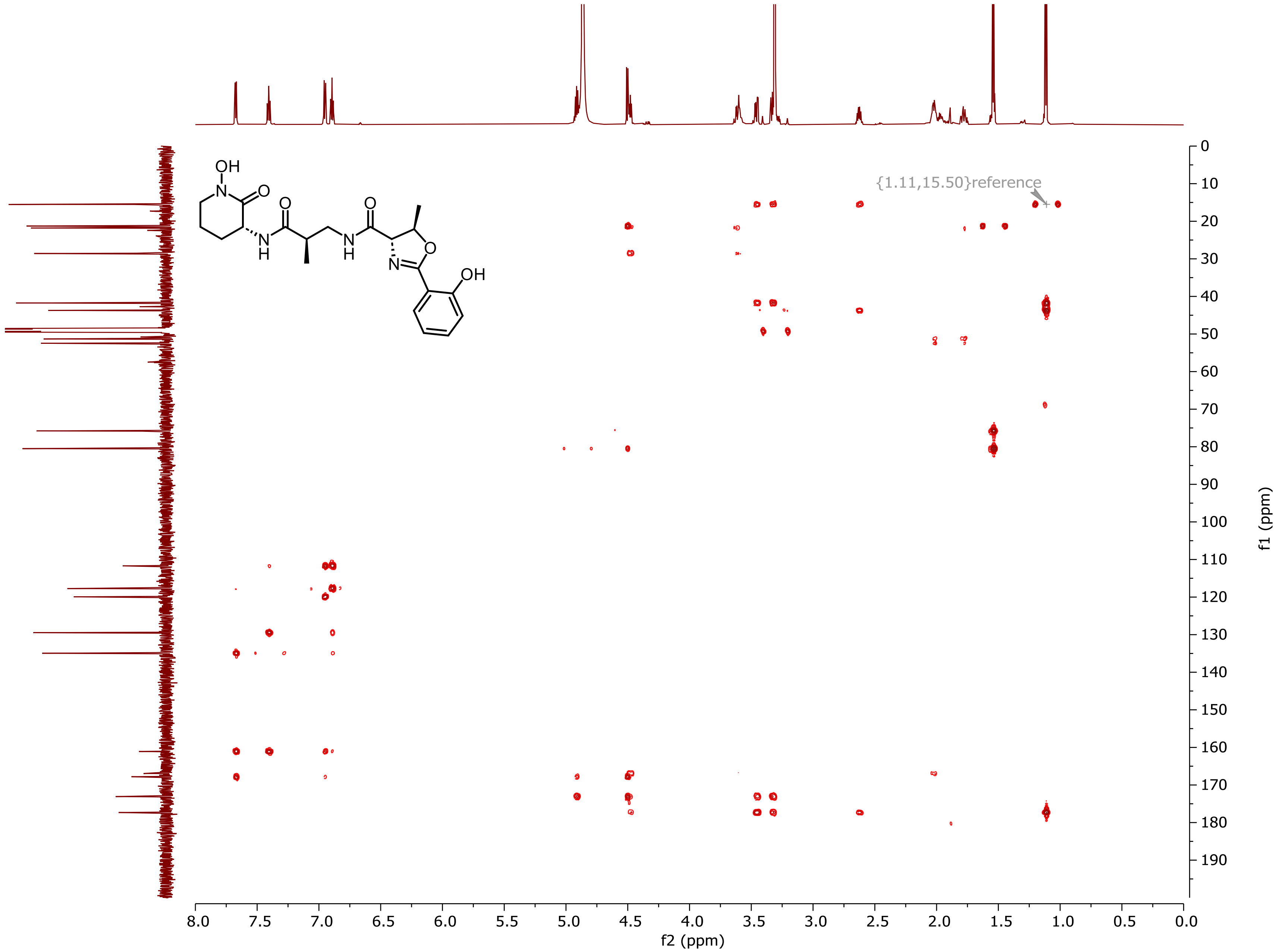

Figure S11 HMBC NMR (CD_3OD, 700 MHz) of kasichelin A (**1**)

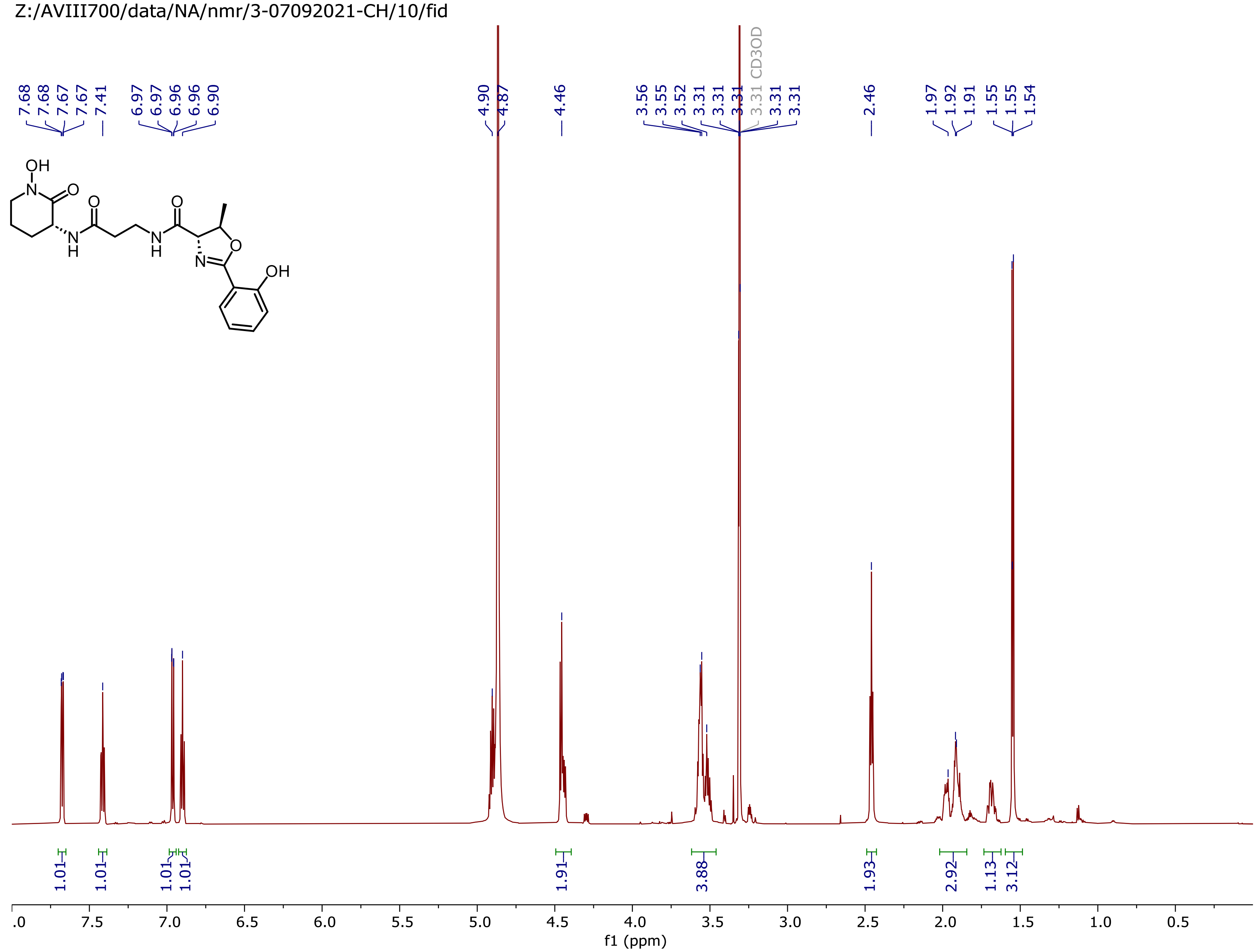

Figure S12 ^{1}H NMR (CD_3OD, 700 MHz) of kasichelin B (**2**)

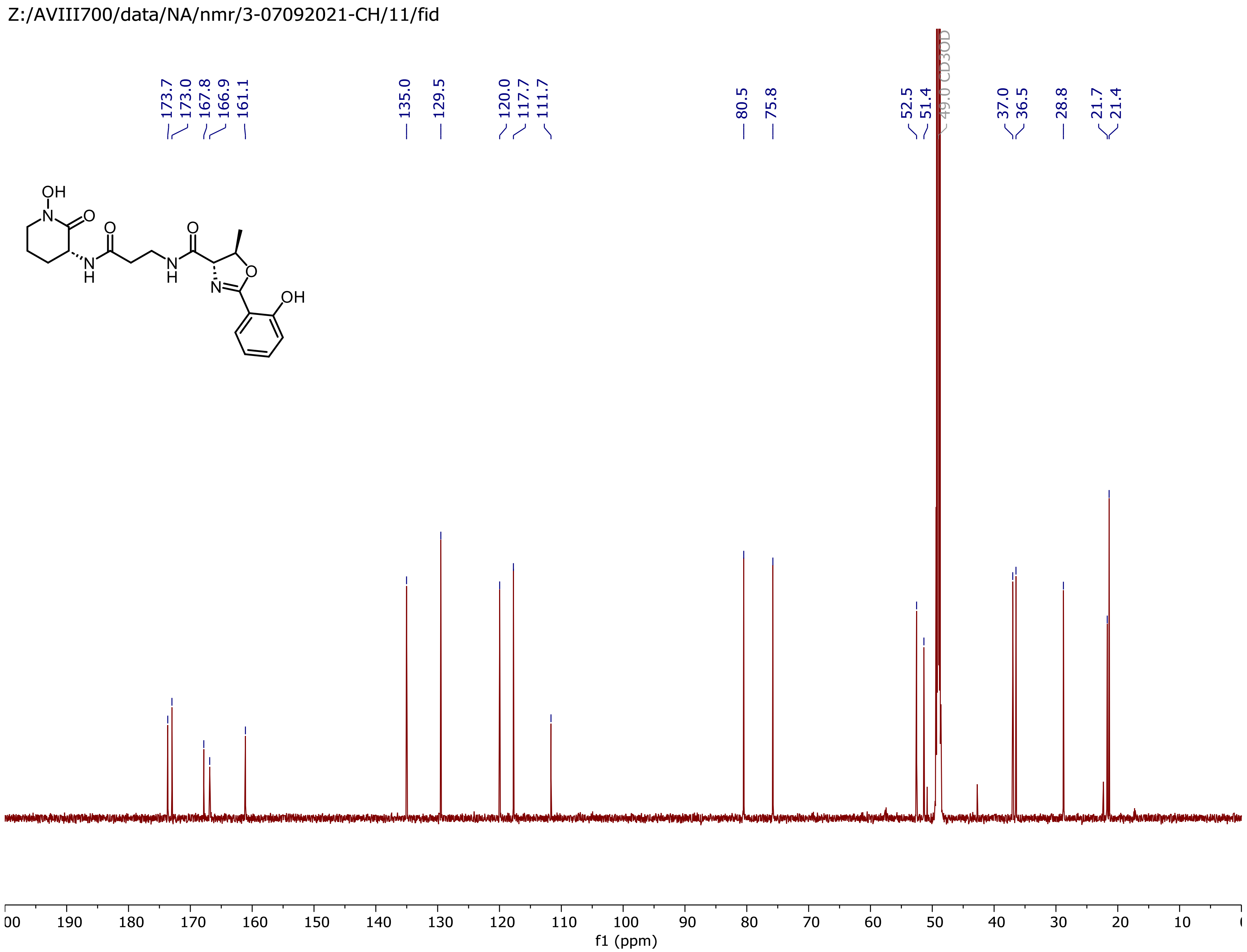

Figure S13 ^{13}C NMR (CD_3OD, 700 MHz) of kasichelin B (**2**)

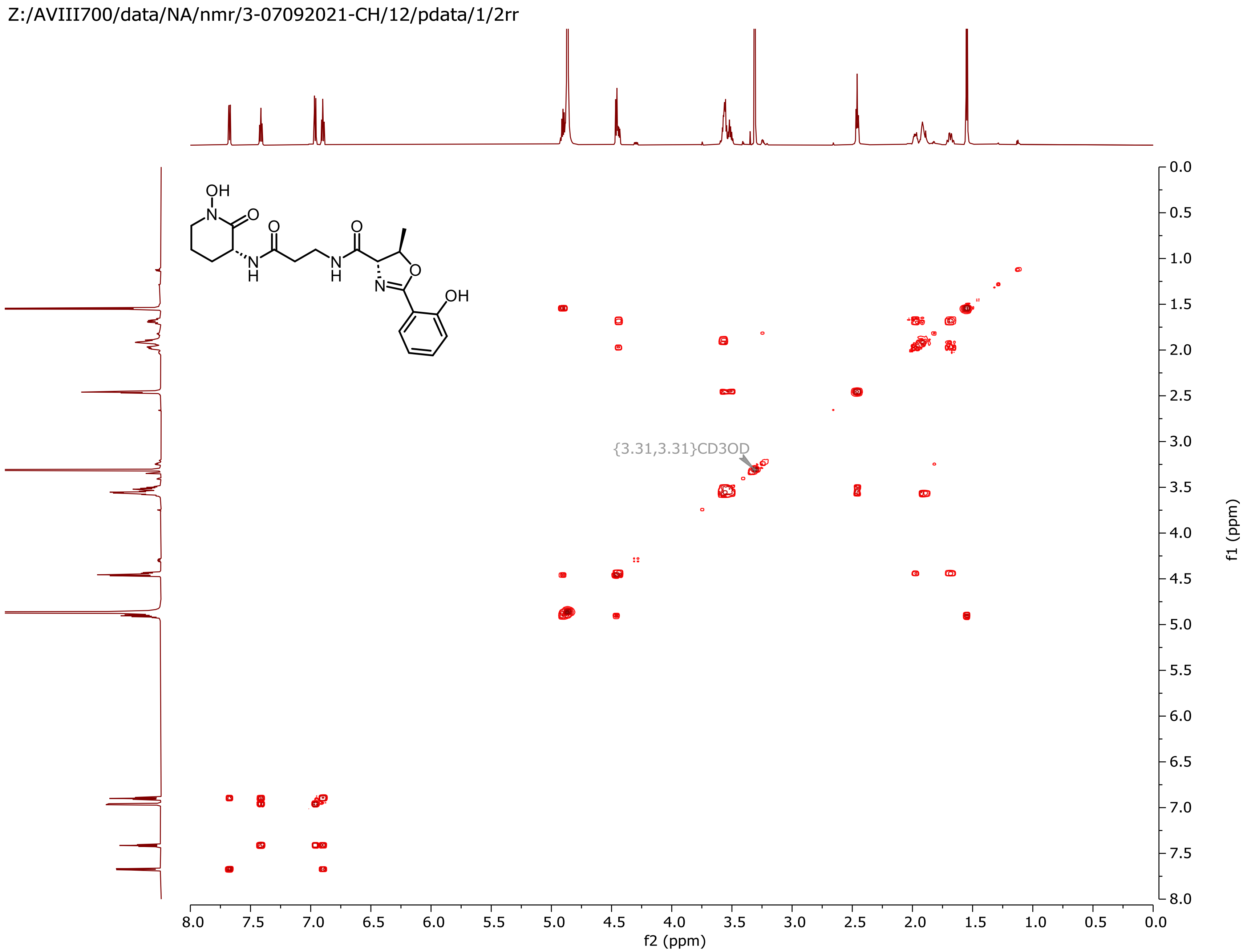

Figure S14 COSY NMR (CD_3OD, 700 MHz) of kasichelin B (**2**)

Z:/AVIII700/data/NA/nmr/3-07092021-CH/13/pdata/1/2rr

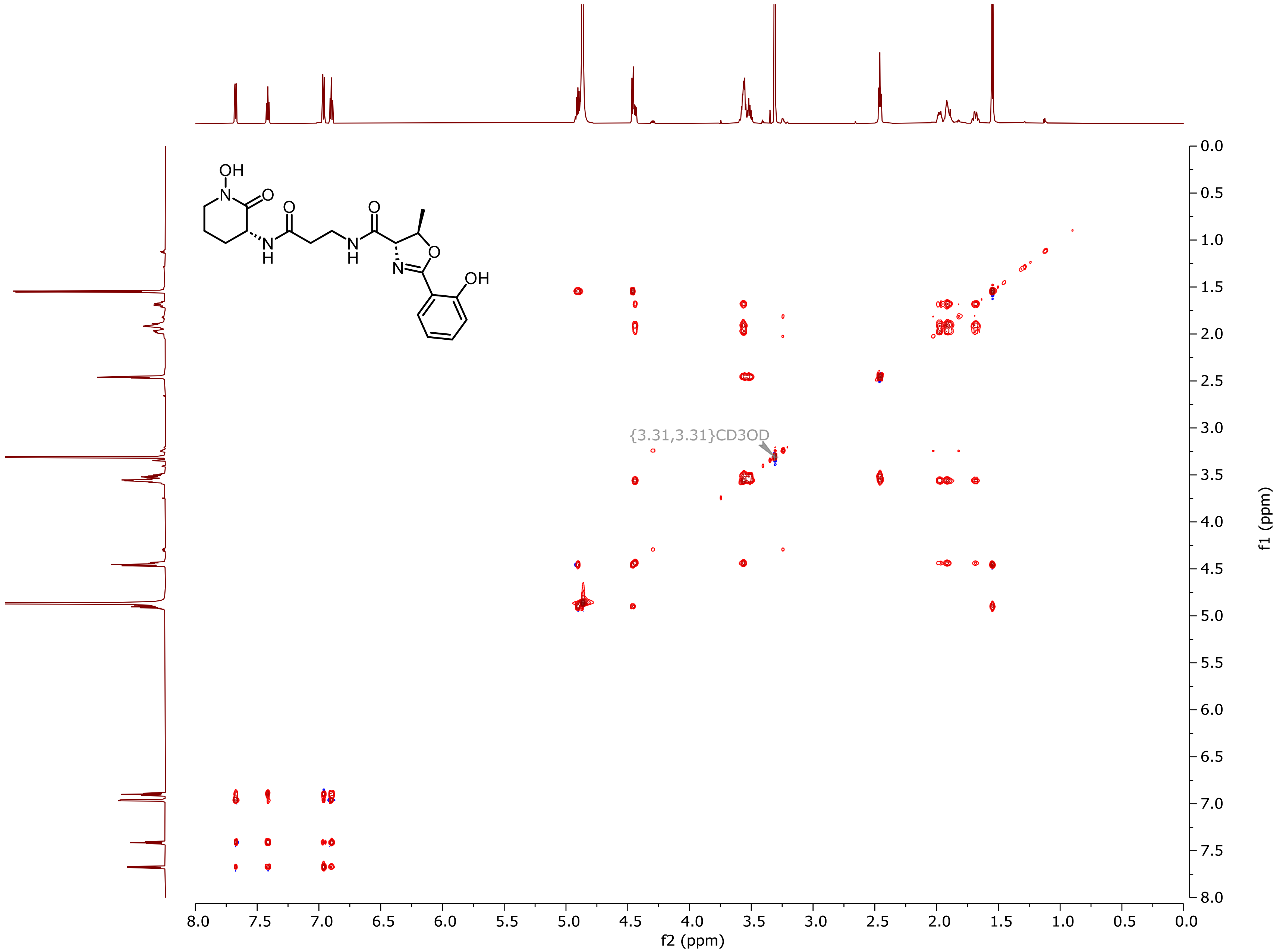

Figure S15 TOCSY NMR (CD_3OD, 700 MHz) of kasichelin B (**2**)

Z:/AVIII700/data/NA/nmr/3-07092021-CH/14/ser

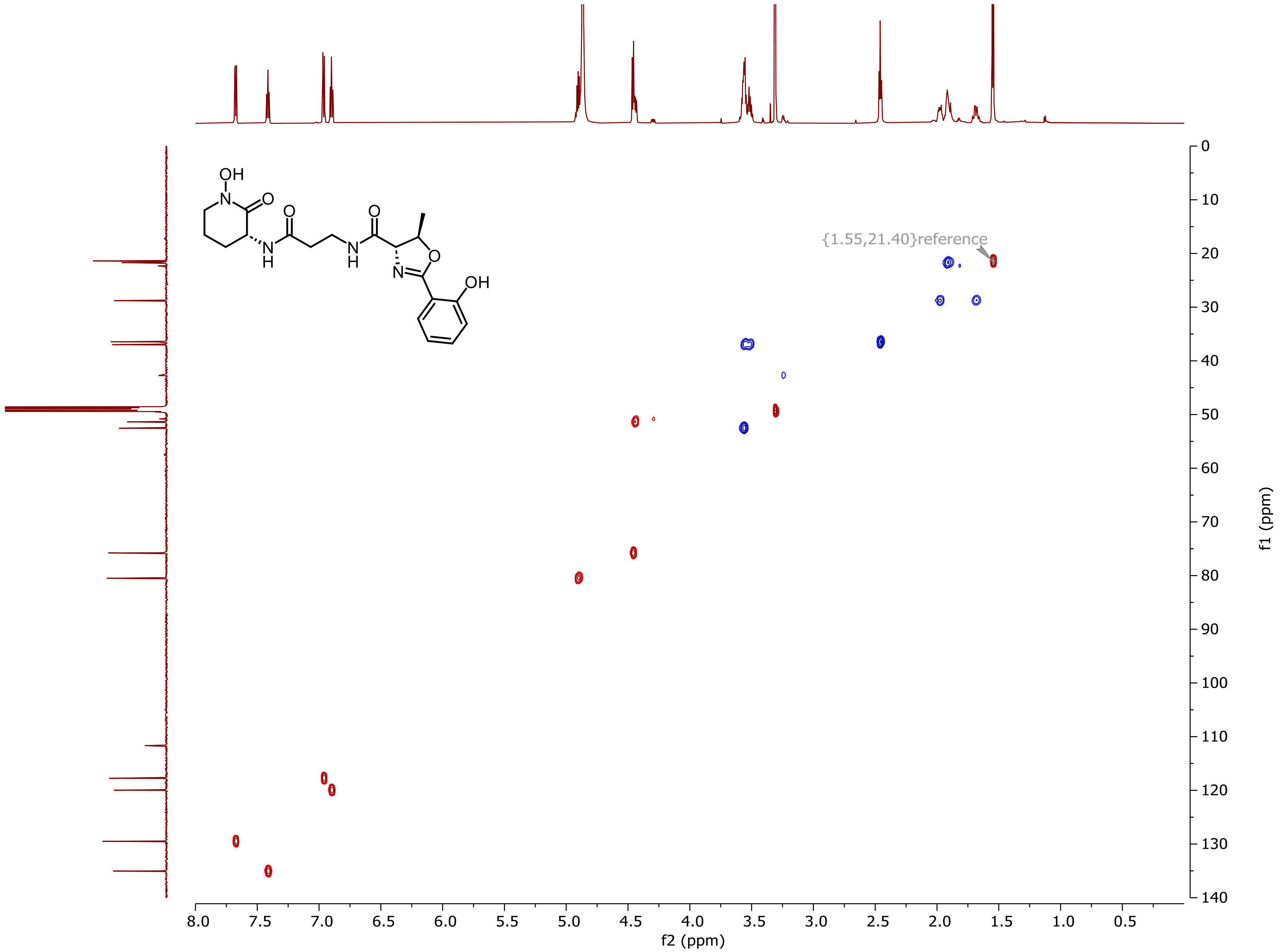

Figure S16 HSQC NMR (CD_3OD, 700 MHz) of kasichelin B (**2**)

Z:/AVIII700/data/NA/nmr/3-07092021-CH/15/pdata/1/2rr

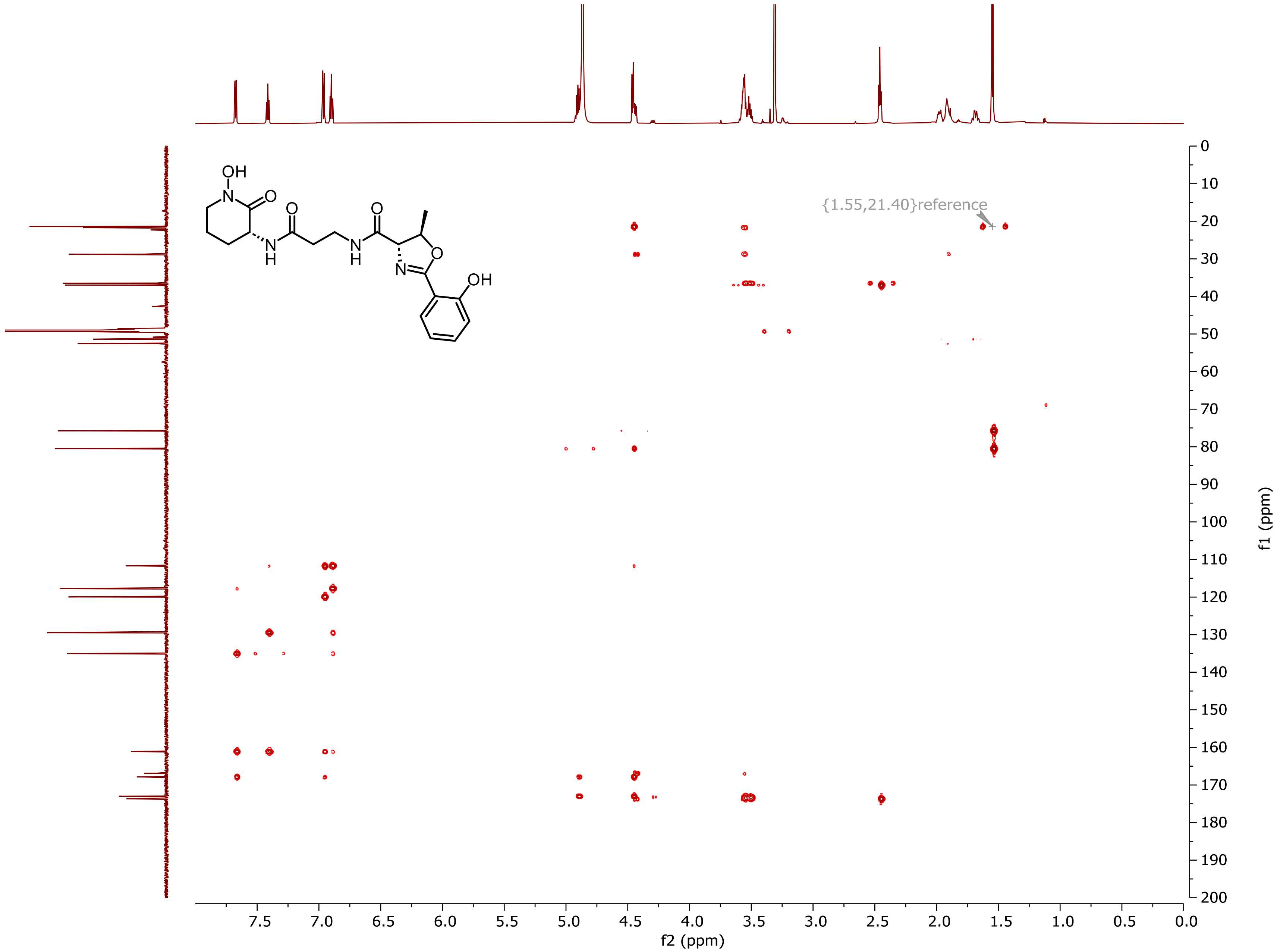

Figure S17 HMBC NMR (CD_3OD, 700 MHz) of kasichelin B (**2**)

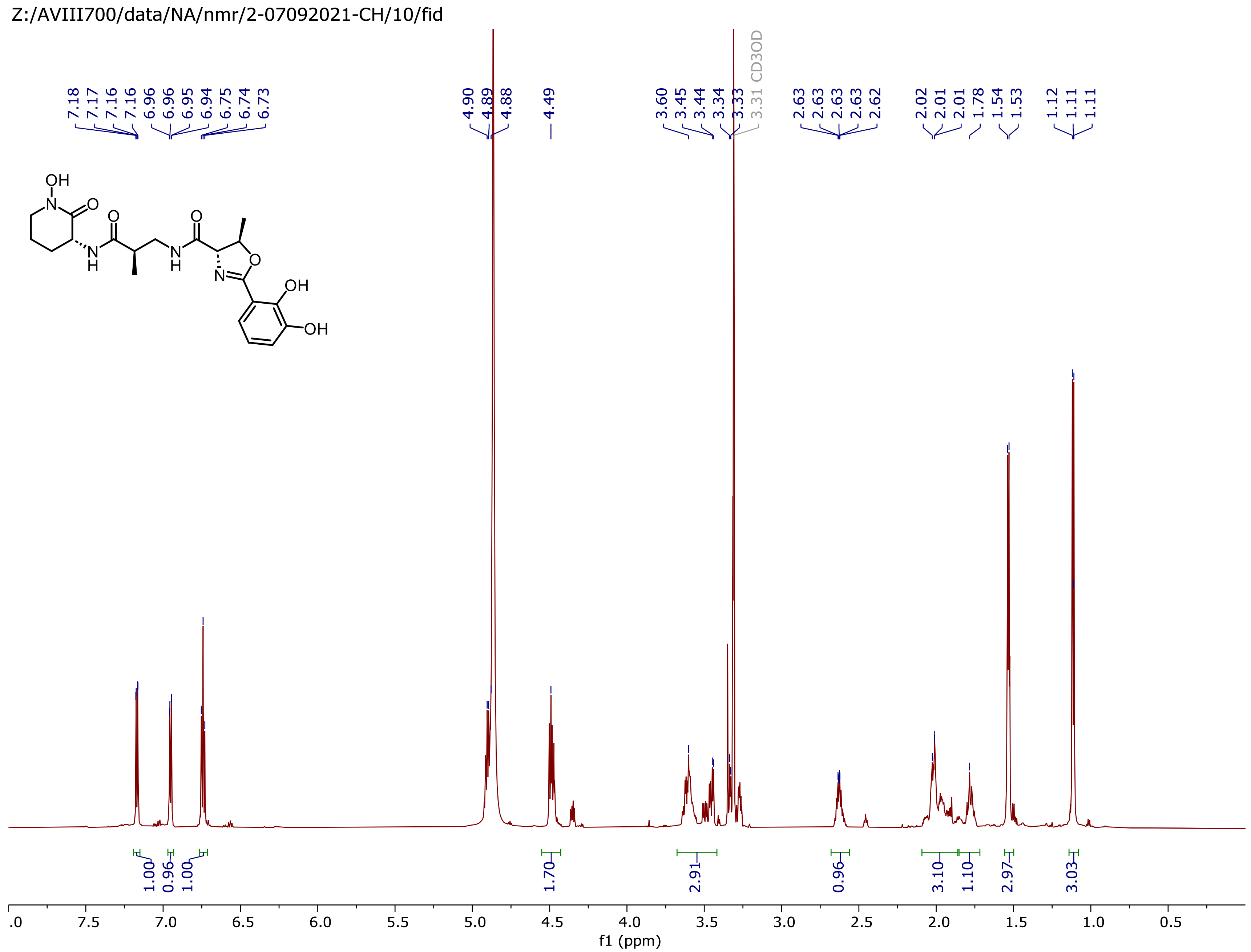

Figure S18 ^{1}H NMR (CD_3OD, 700 MHz) of kasichelin C (**3**)

Z:/AVIII700/data/NA/nmr/2-07092021-CH/11/pdata/1/1r

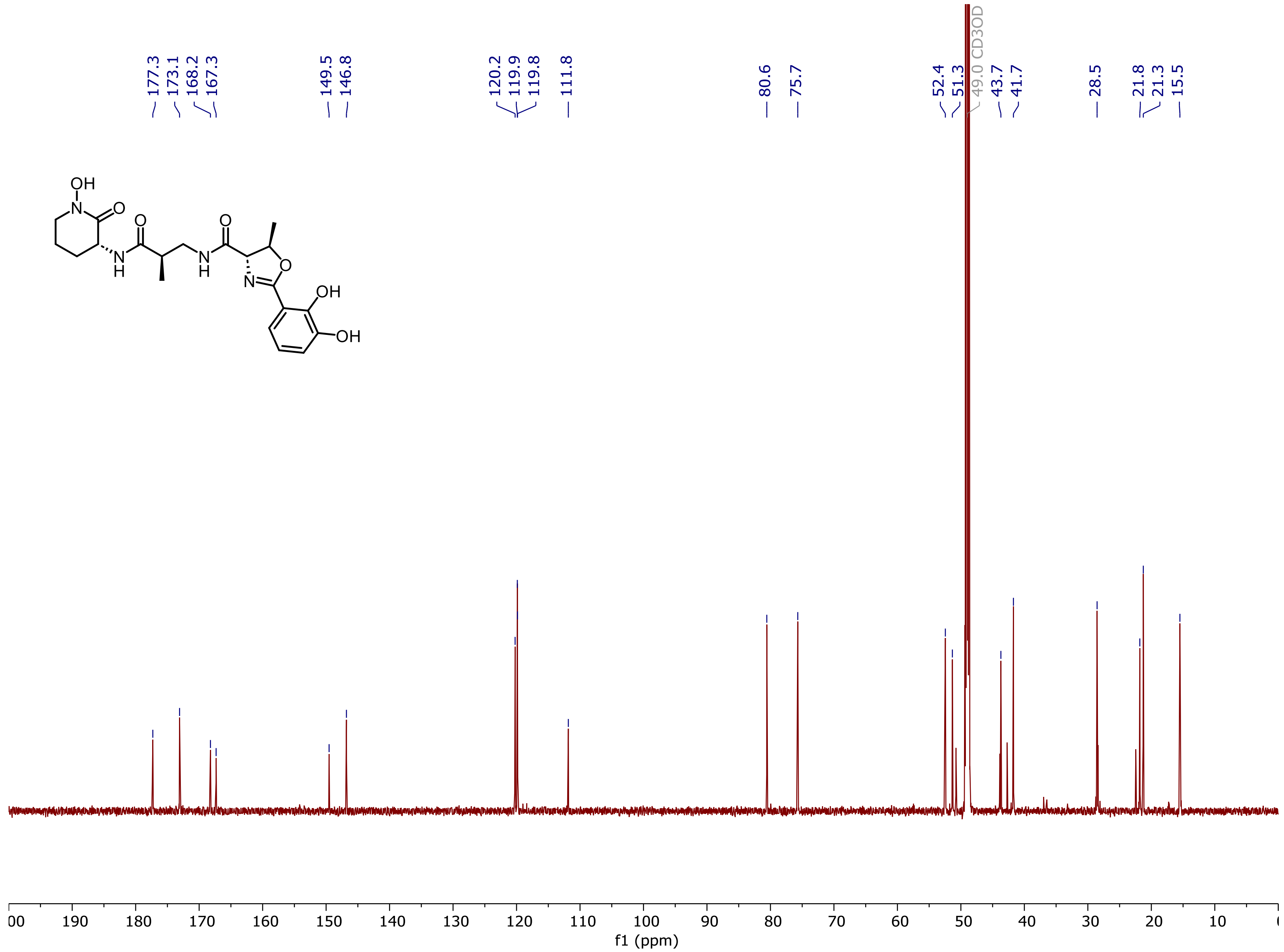

Figure S19 ^{13}C NMR (CD_3OD, 700 MHz) of kasichelin C (**3**)

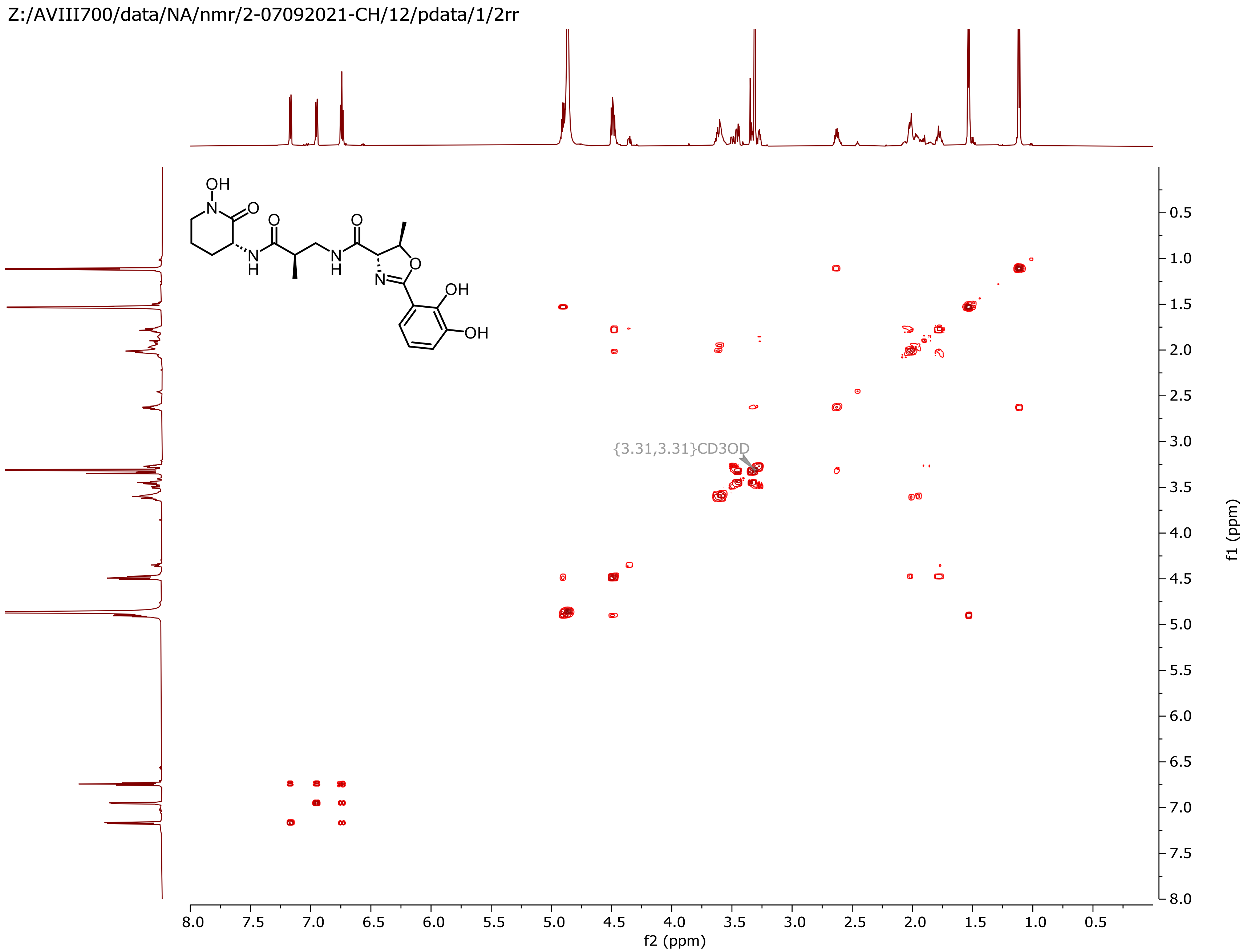

Figure S20 COSY NMR (CD_3OD, 700 MHz) of kasichelin C (**3**)

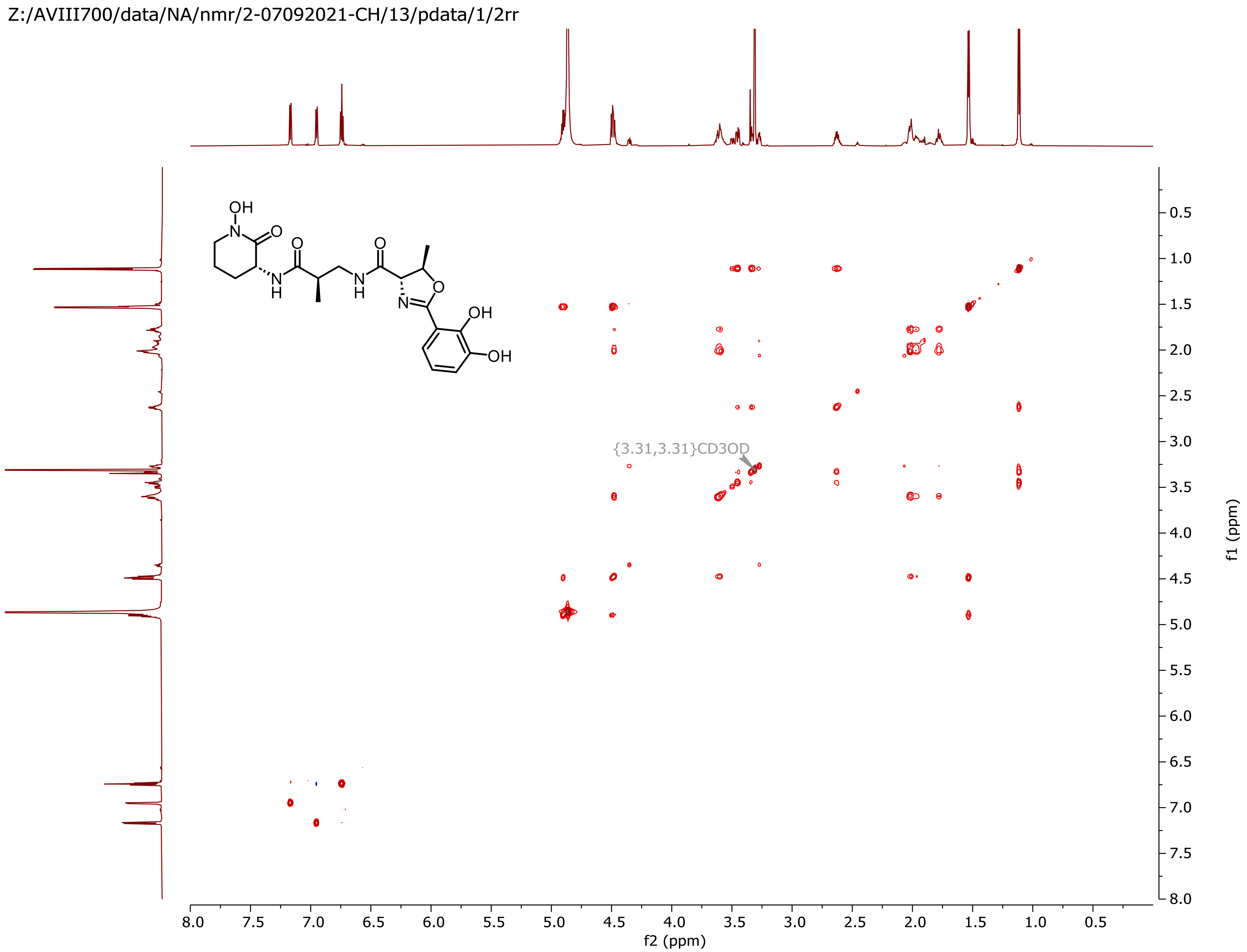

Figure S21 TOCSY NMR (CD_3OD, 700 MHz) of kasichelin C (**3**)

Z:/AVIII700/data/NA/nmr/2-07092021-CH/14/ser

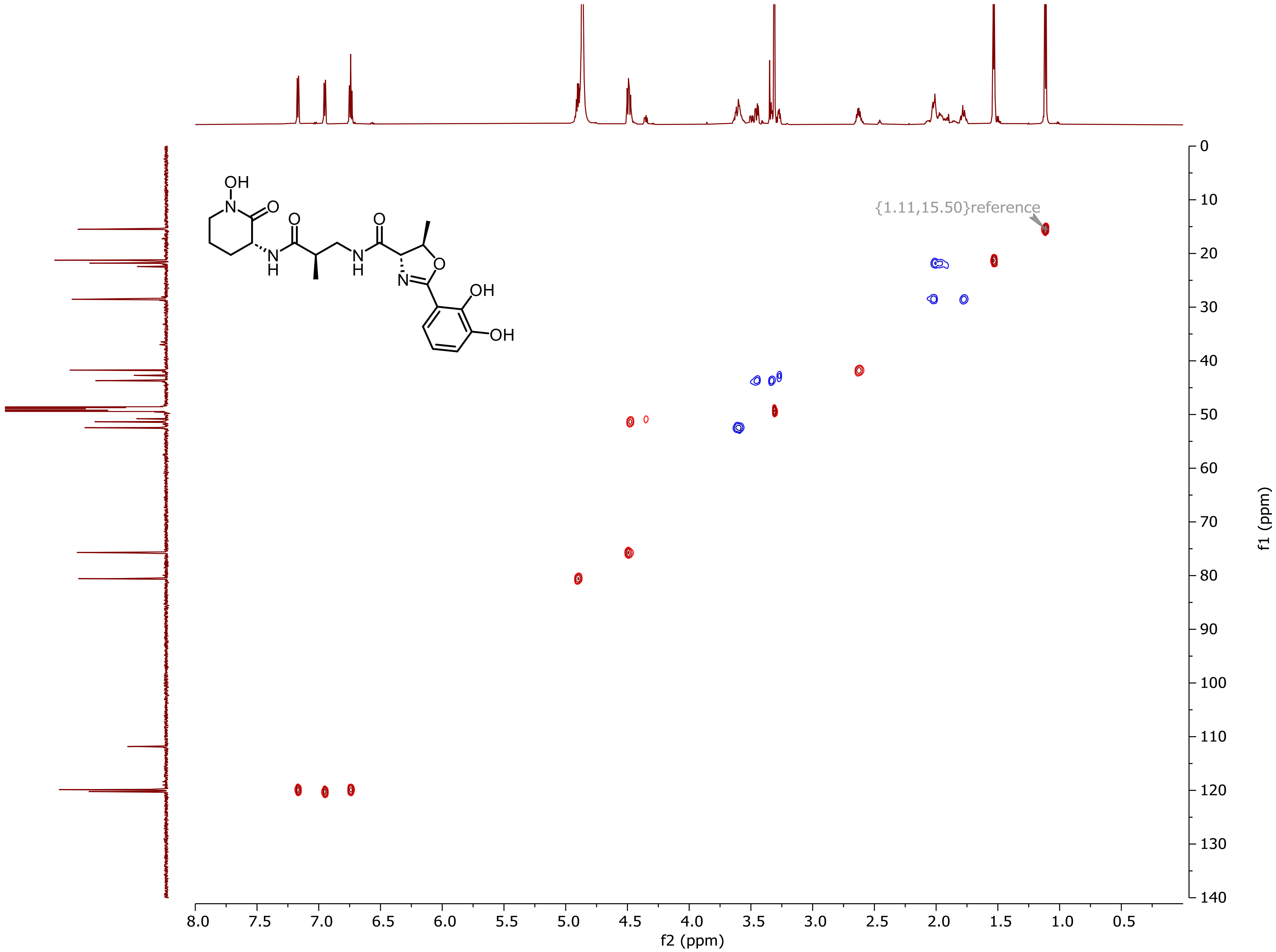

Figure S22 HSQC NMR (CD_3OD, 700 MHz) of kasichelin C (**3**)

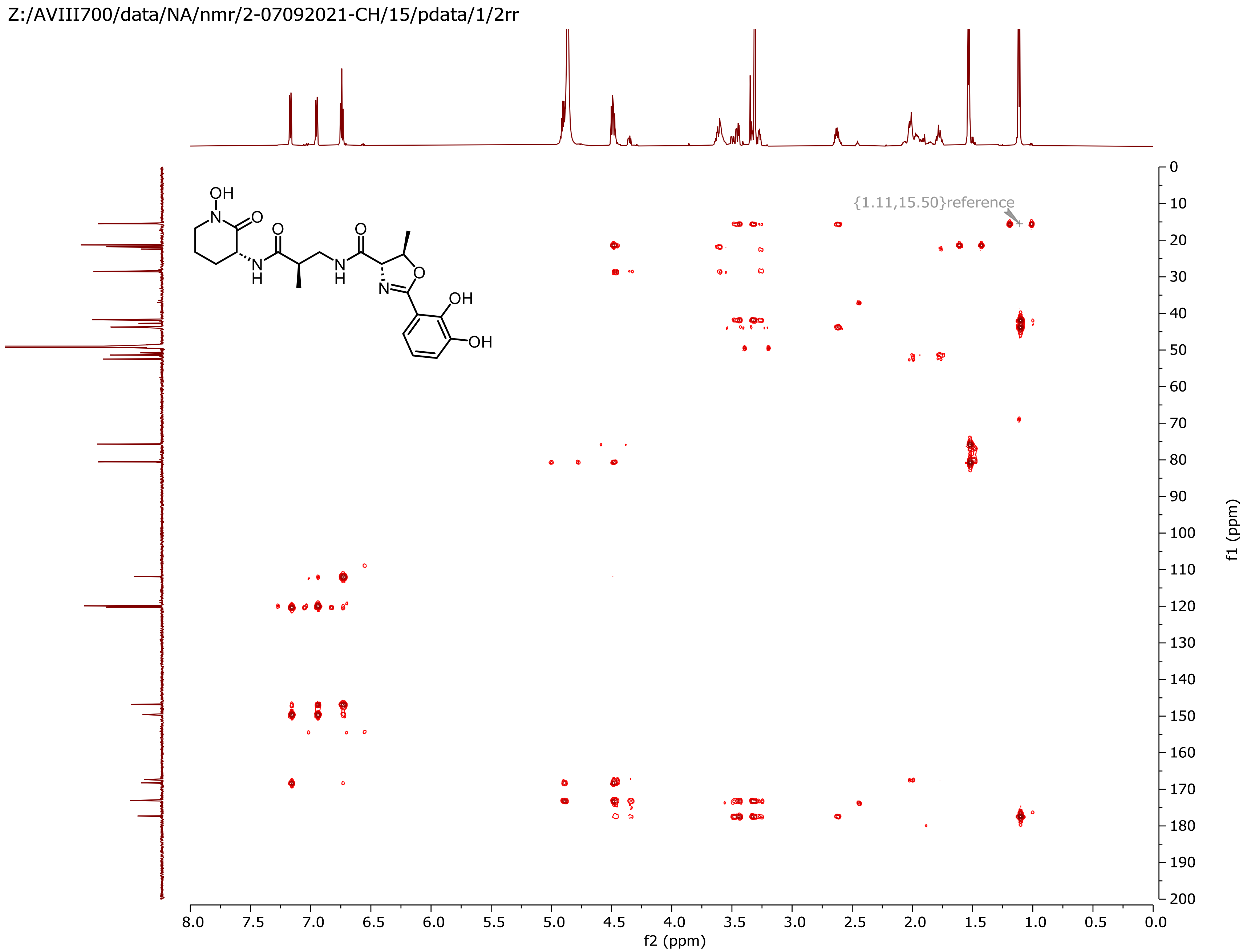

Figure S23 HMBC NMR (CD_3OD, 700 MHz) of kasichelin C (**3**)

Z:/AVIII700/data/NA/nmr/1-07092021-CH/10/fid

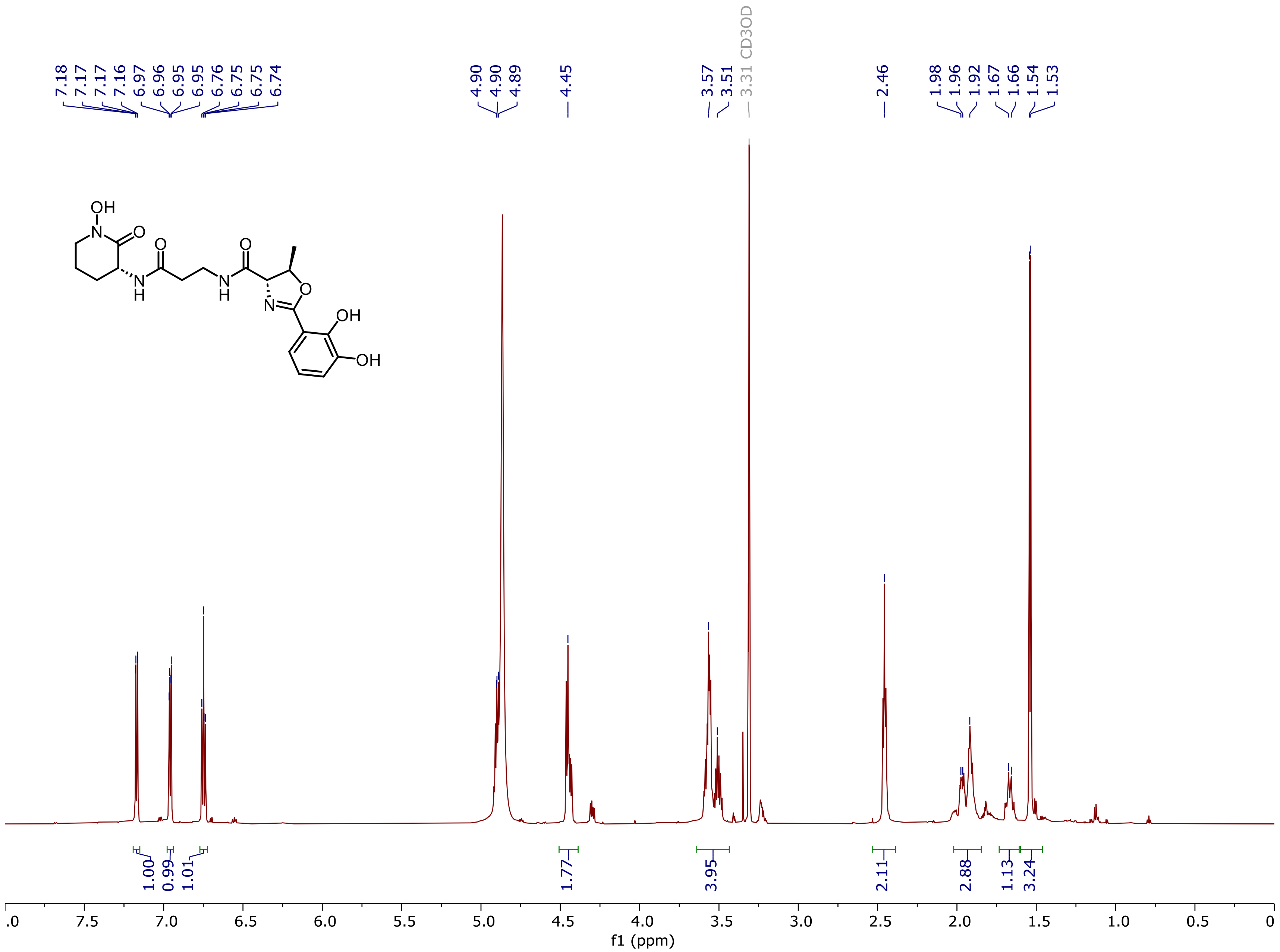

Figure S24 ^{1}H NMR (CD_3OD, 700 MHz) of kasichelin D (**4**)

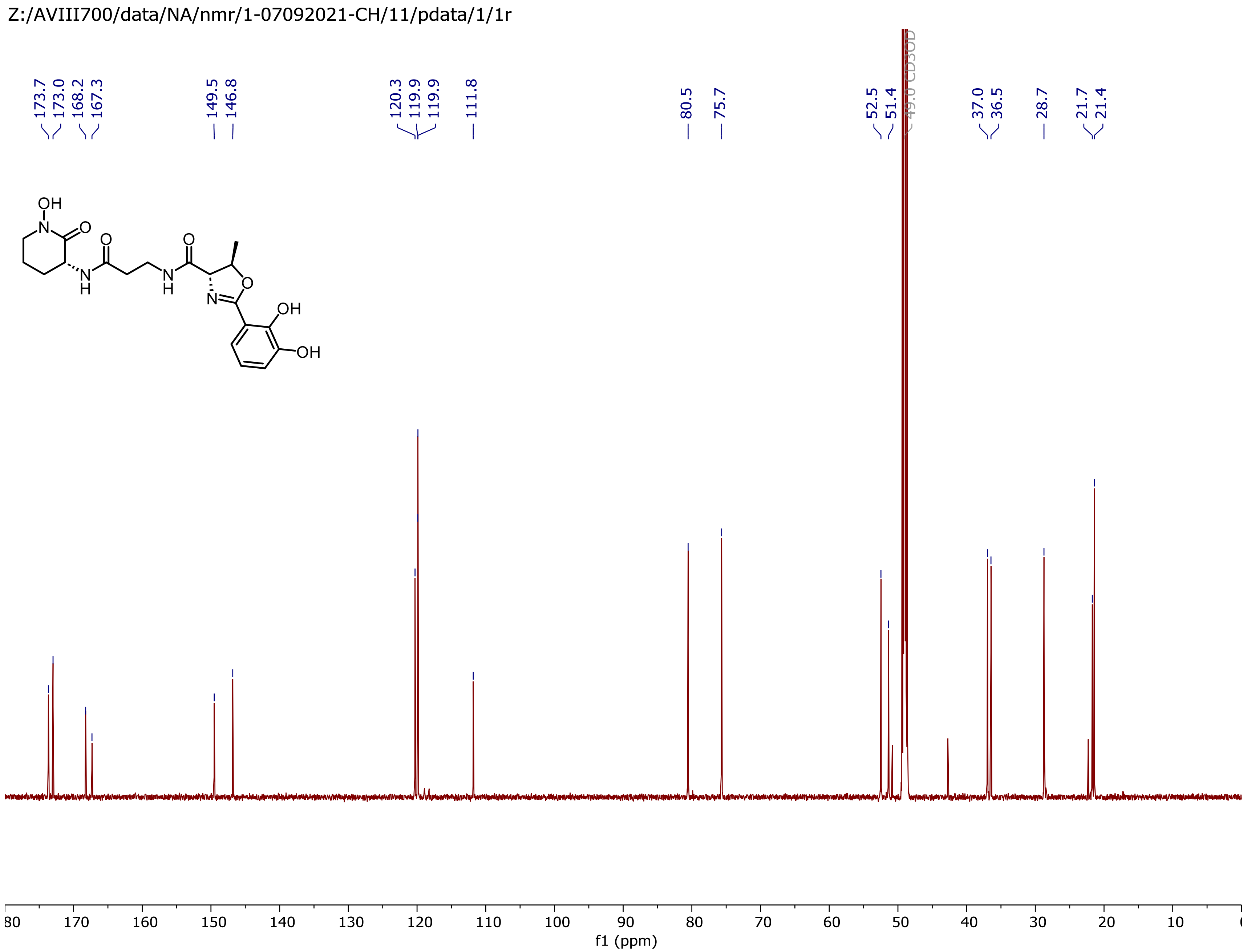

Figure S25 ^{13}C NMR (CD_3OD, 700 MHz) of kasichelin D (**4**)

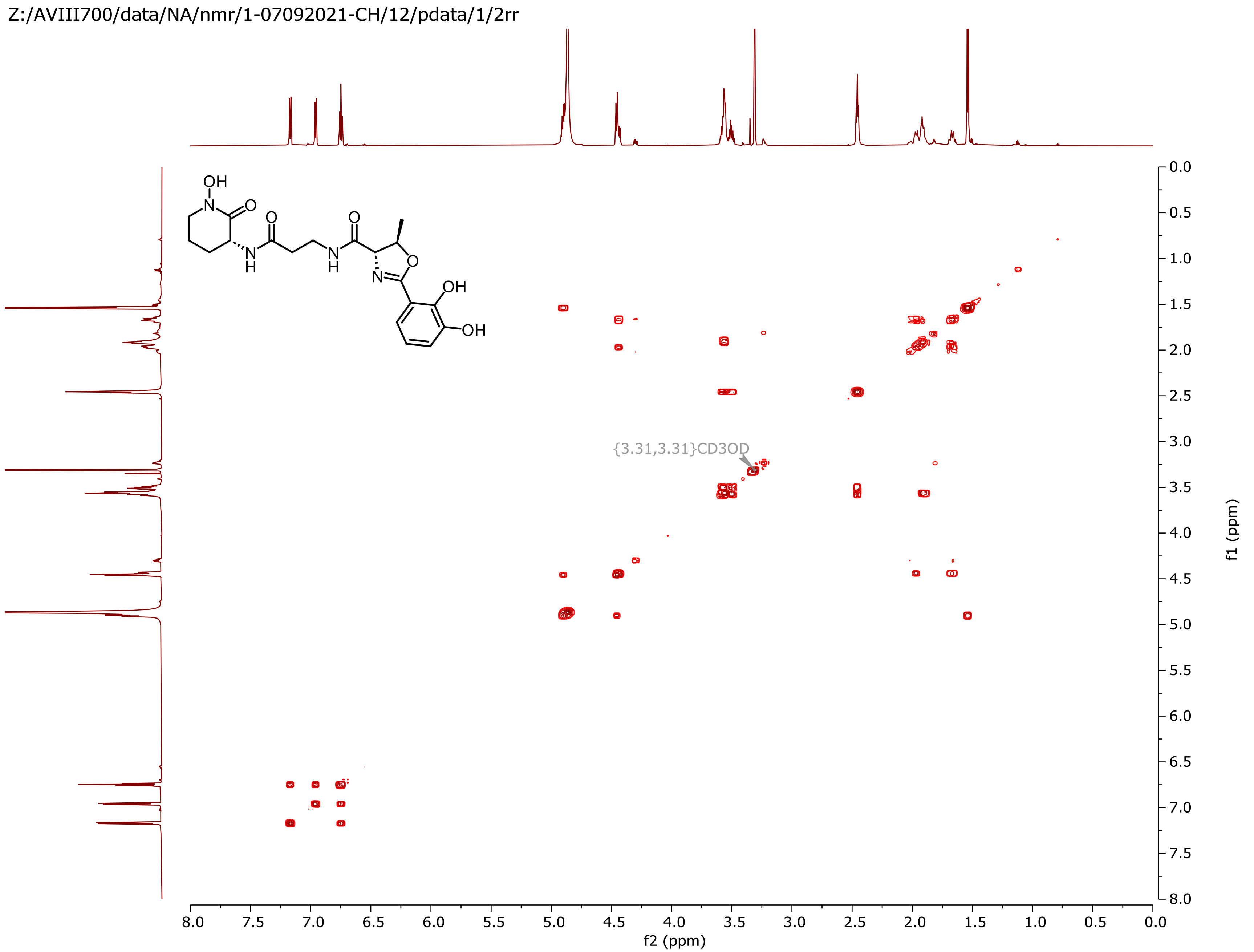

Figure S26 COSY NMR (CD_3OD, 700 MHz) of kasichelin D (**4**)

Z:/AVIII700/data/NA/nmr/1-07092021-CH/13/pdata/1/2rr

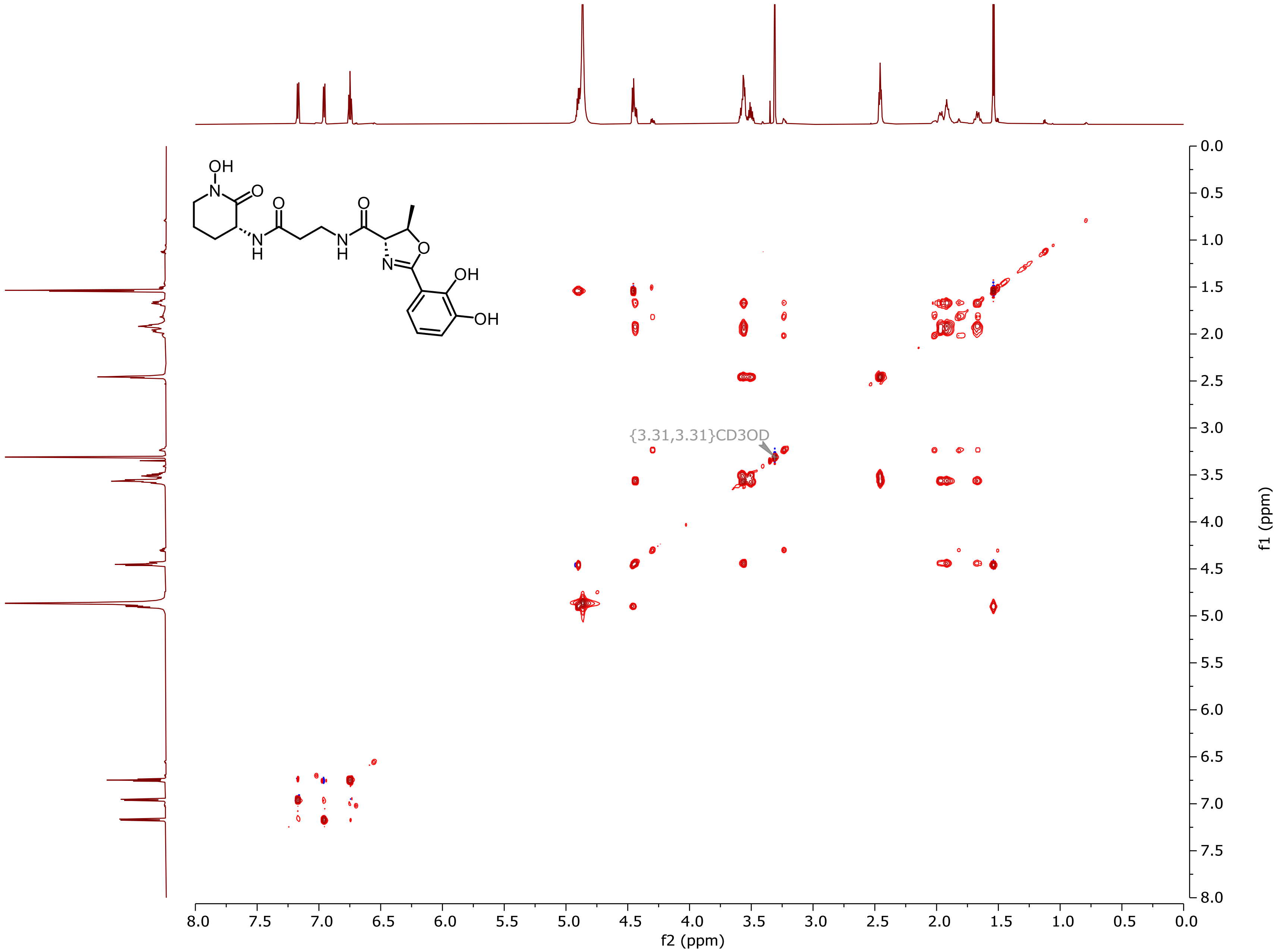

Figure S27 TOCSY NMR (CD_3OD, 700 MHz) of kasichelin D (**4**)

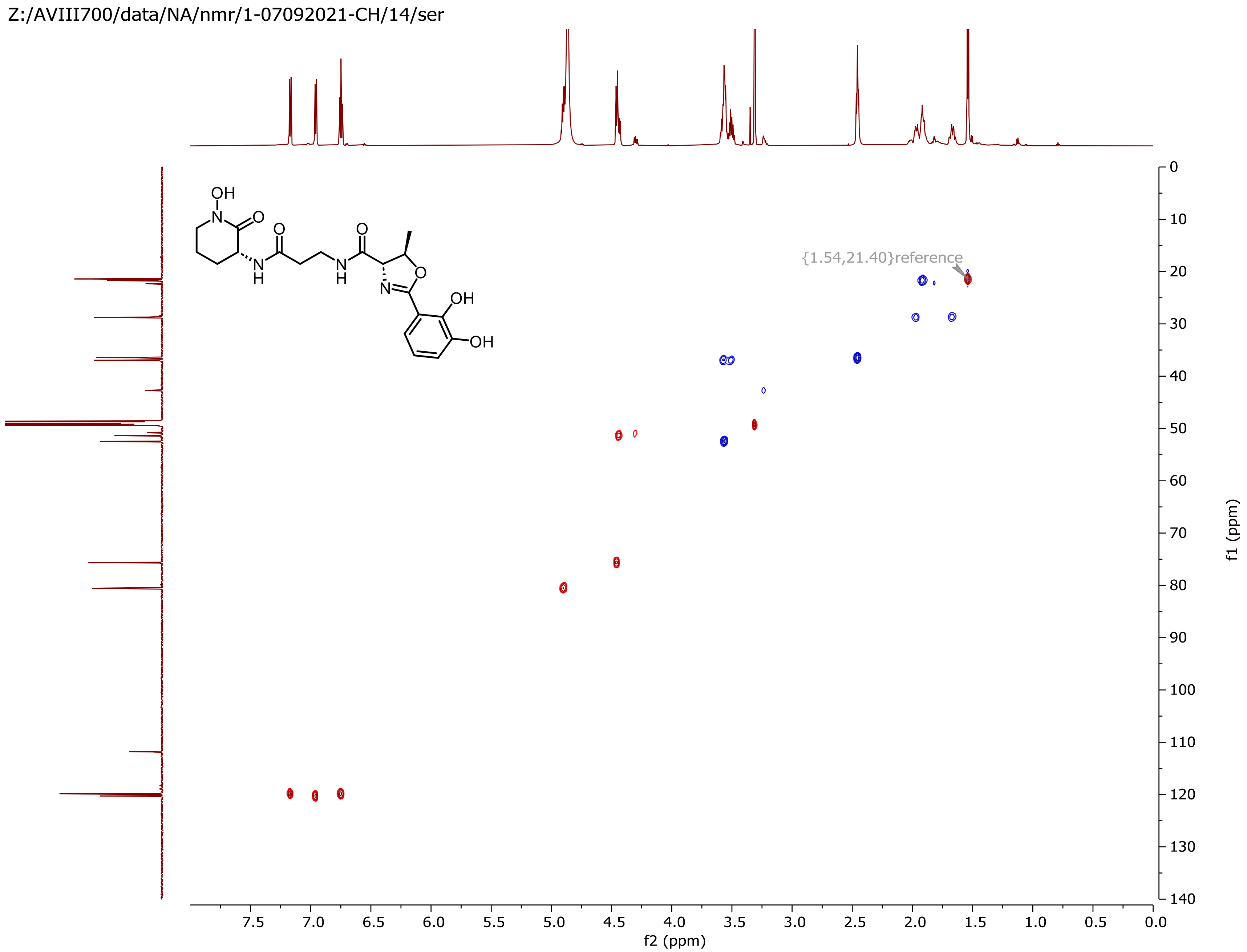

Figure S28 HSQC NMR (CD_3OD, 700 MHz) of kasichelin D (**4**)

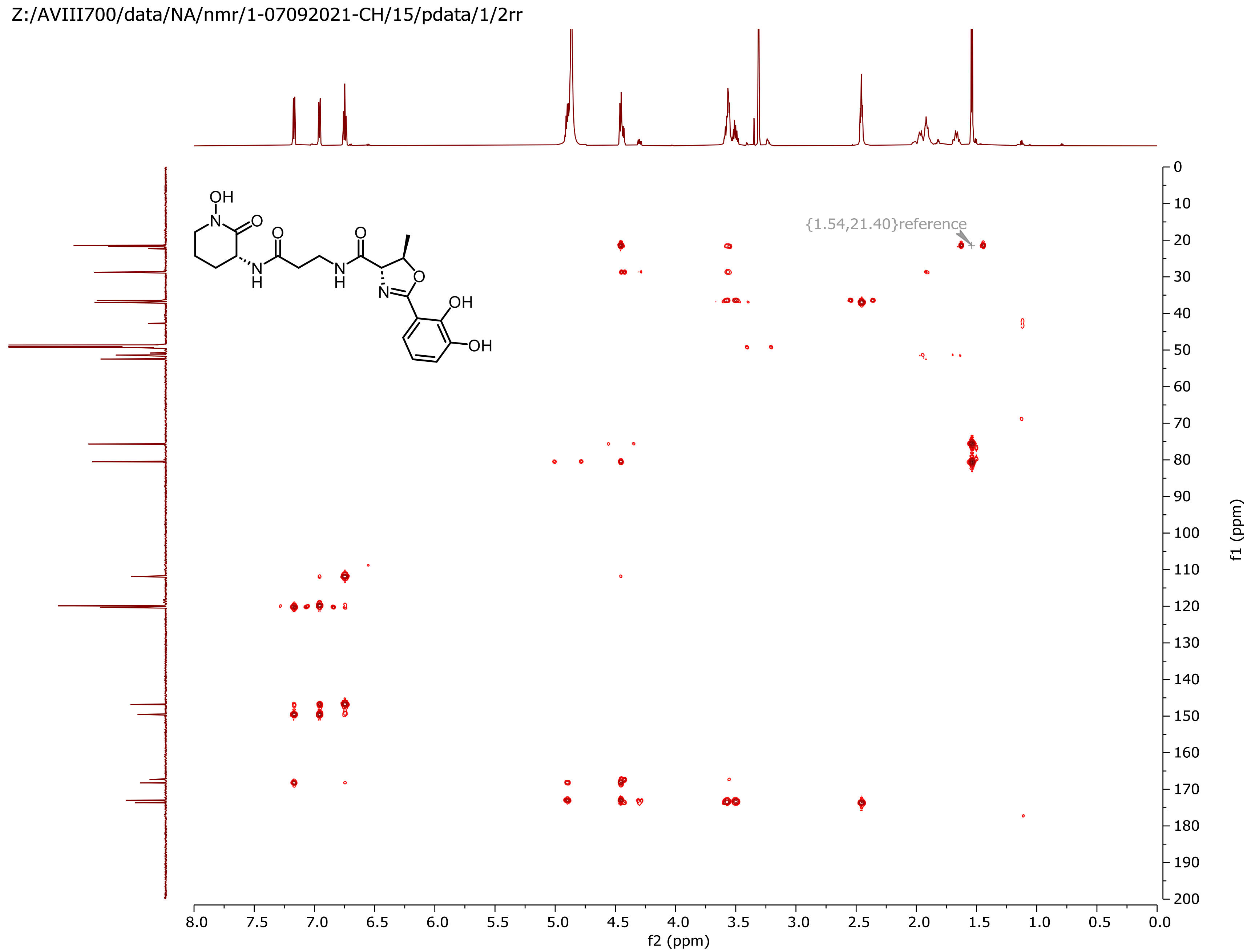

Figure S29 HMBC NMR (CD_3OD, 700 MHz) of kasichelin D (**4**)

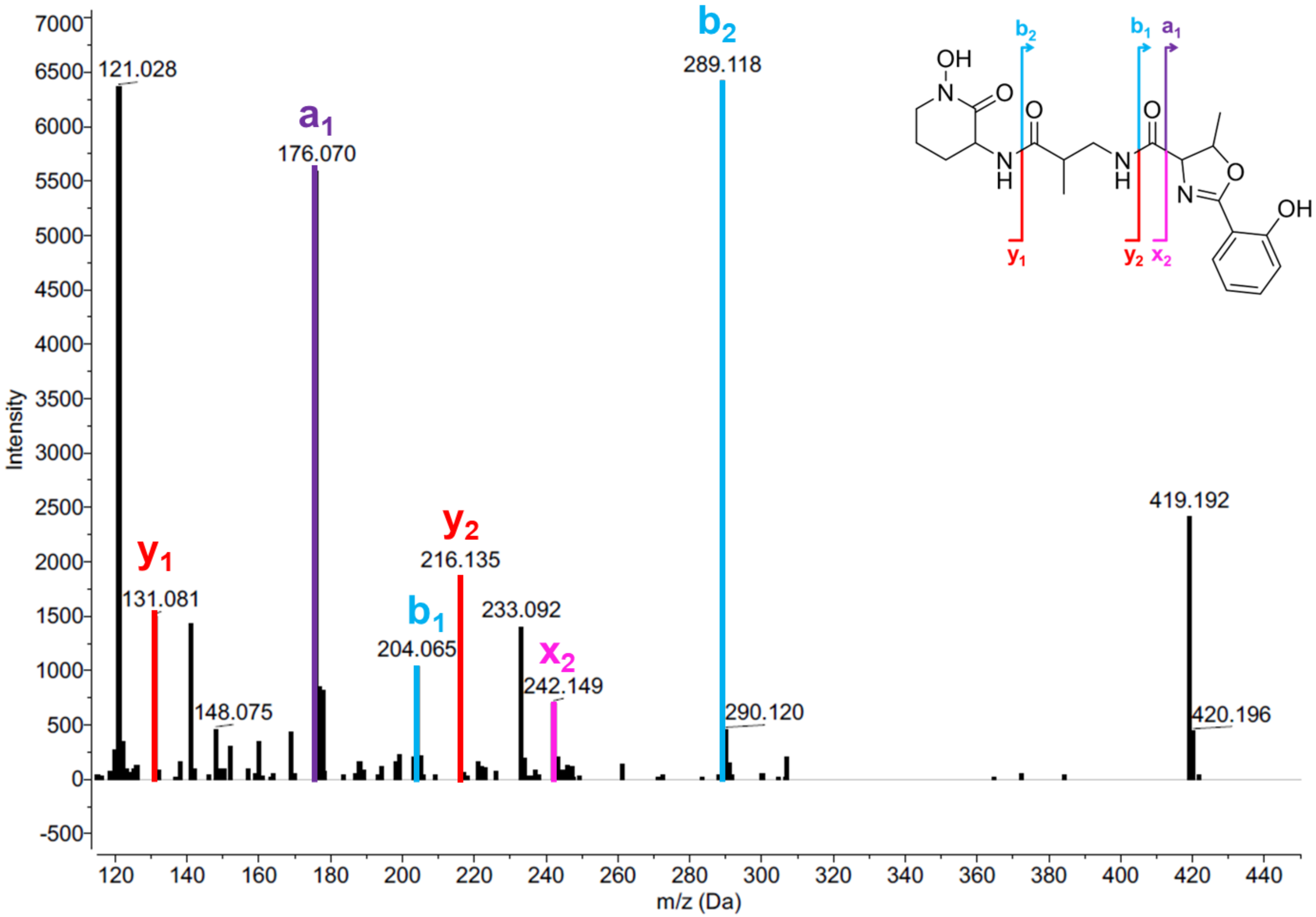

Figure S30 MS/MS fragmentation of kasichelin A (**1**)

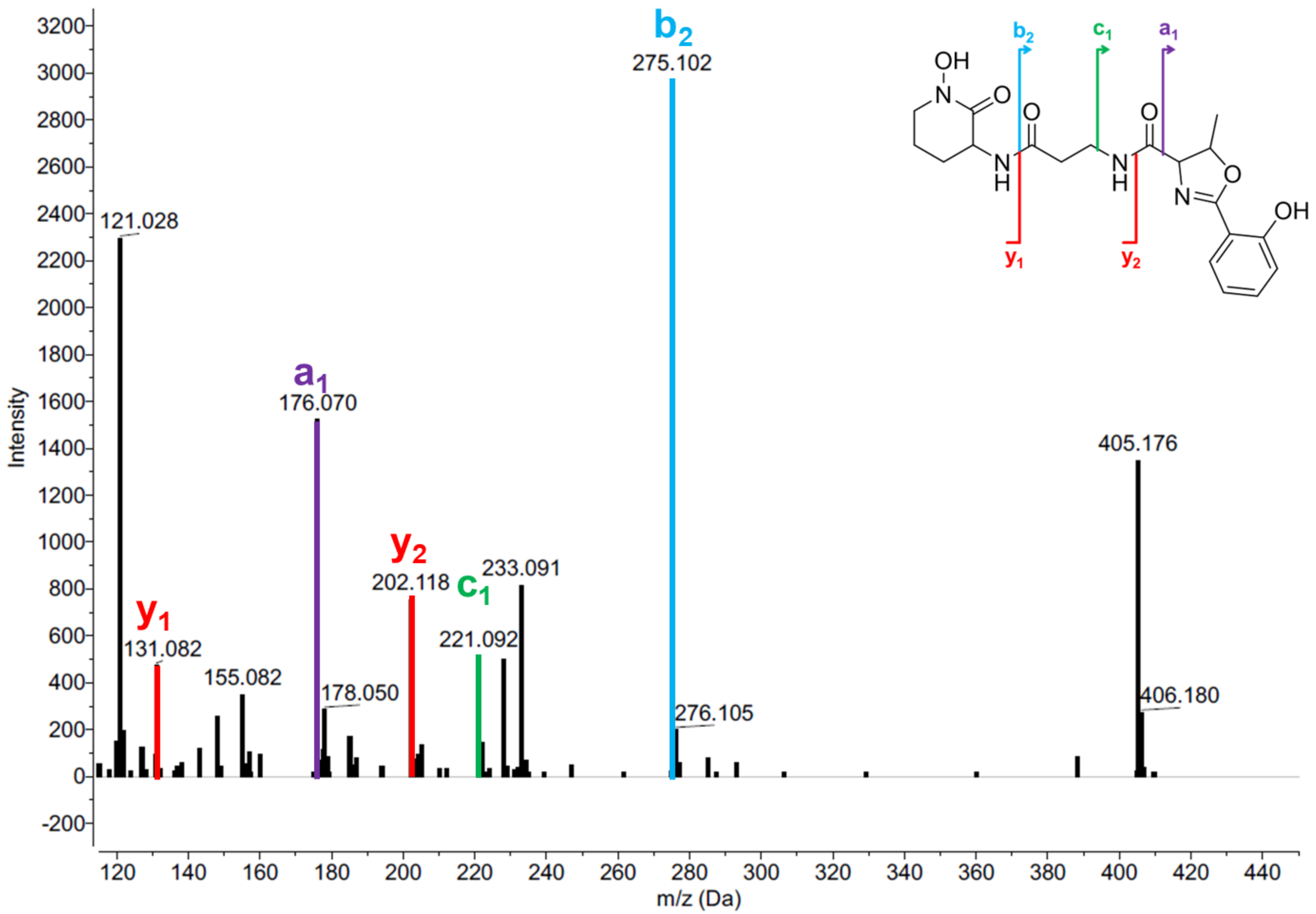

Figure S31 MS/MS fragmentation of kasichelin B (**2**)

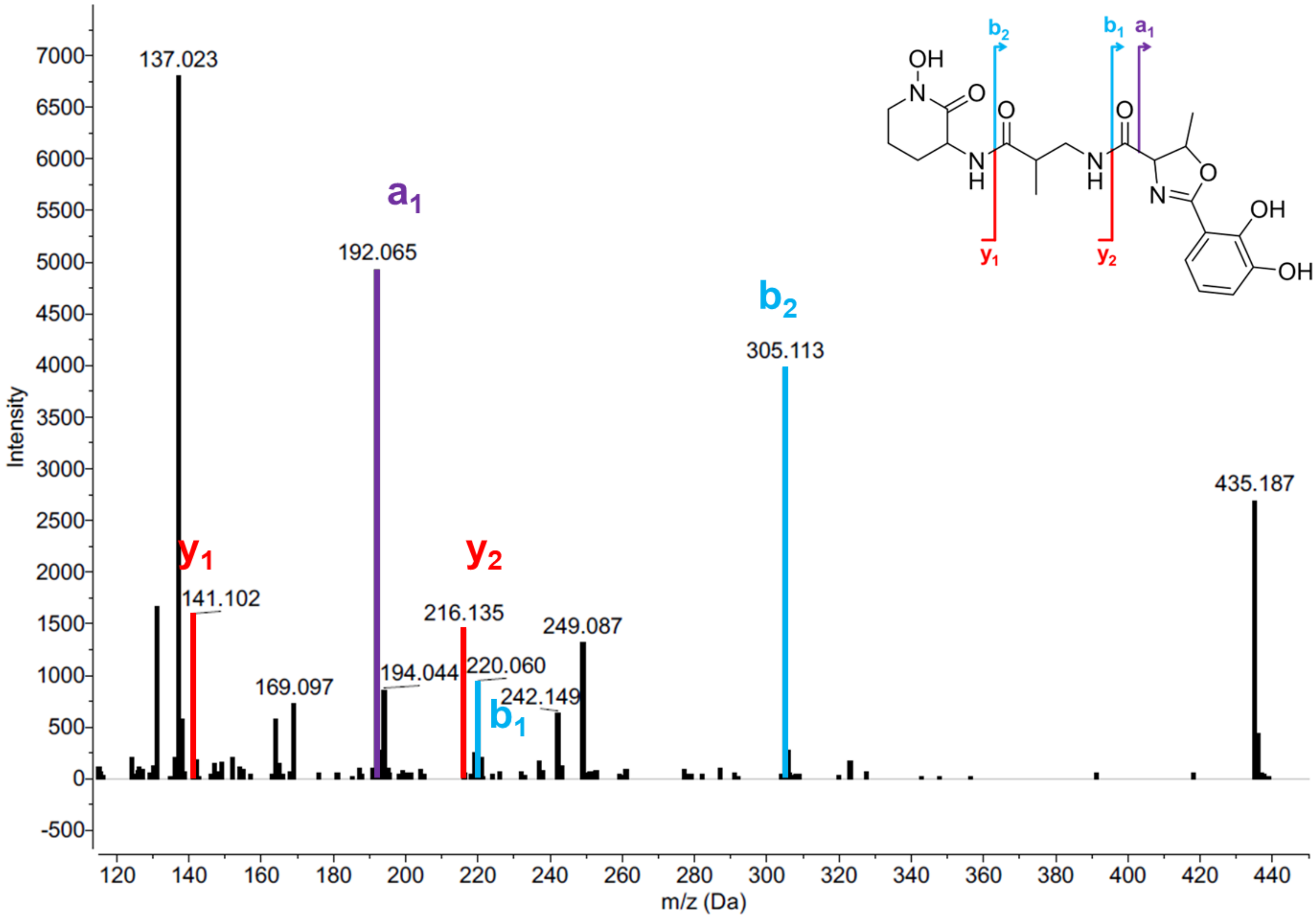

Figure S32 MS/MS fragmentation of kasichelin C (**3**)

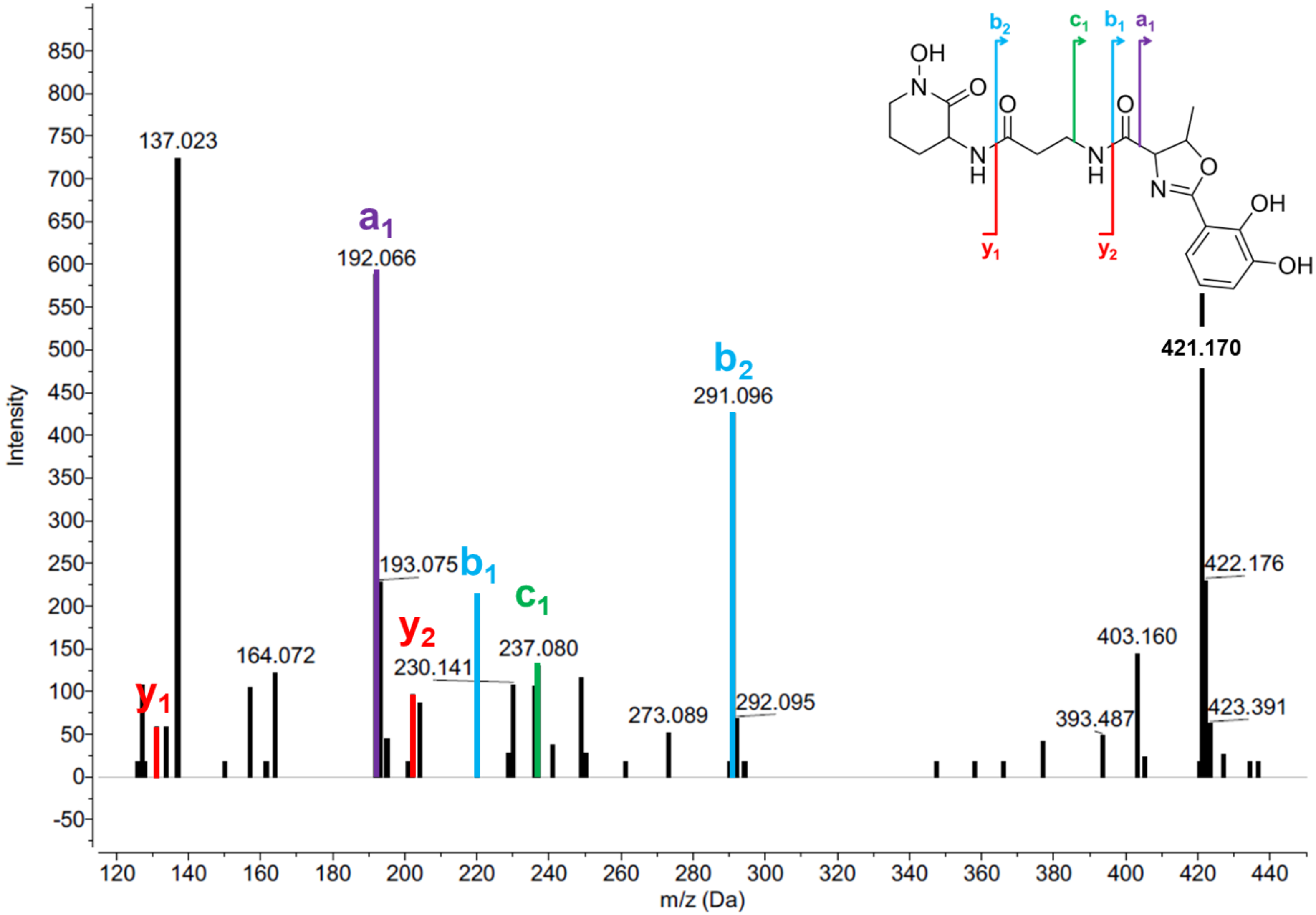

Figure S33 MS/MS fragmentation of kasichelin D (**4**)

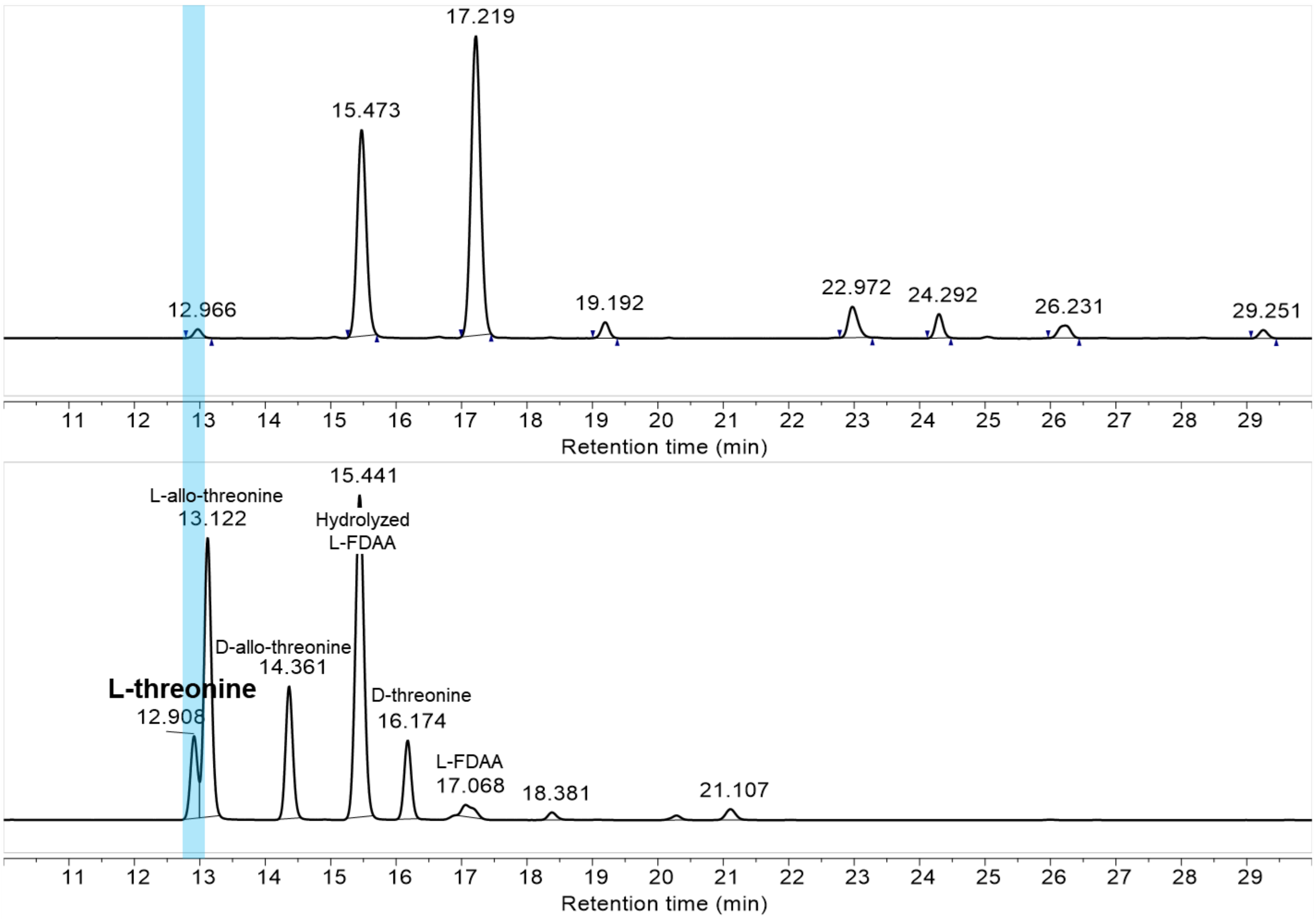

Figure S34 Marfey's analysis of kasichelin C (**3**) (top) and threonine standards (bottom). The retention time of FDAA-labeled L-threonine was 12.9 min. The retention times of the other FDAA-labeled threonine were 13.1 min (L-allo-threonine), 14.4 min (D-allo-threonine), and 16.2 min (D-threonine). Hydrolyzed L-FDAA and L-FDAA eluted at 15.5 min and 17.2 min.
10 – 60 % ACN in H_2O with 0.1% formic acid over 50 minutes.

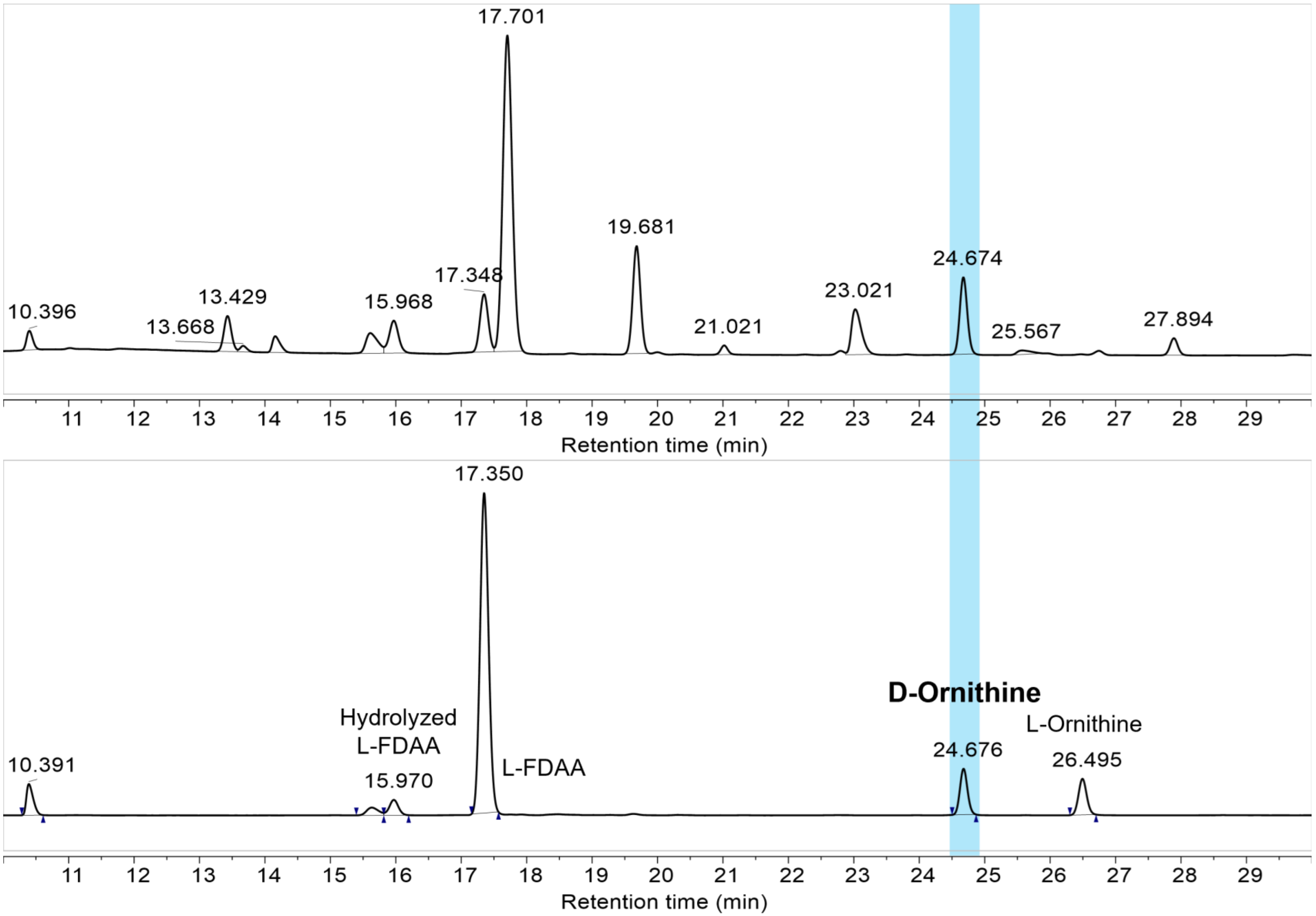

Figure S35 Marfey's analysis of kasichelin C (**3**) (top) and ornithine standards (bottom). The retention time of double-FDAA-labeled D-ornithine was 24.7 min. The retention time of double-FDAA-labeled-L-ornithine was 26.5 min. Hydrolyzed L-FDAA and L-FDAA eluted at 16.00 min and 17.4 min. ** The peak at 26.5 minutes, corresponding to the retention time of double-FDAA-derivatized L-ornithine, increased in intensity with longer hydrolysis time.
10 – 60 % ACN in H_2O with 0.1% formic acid over 50 minutes.

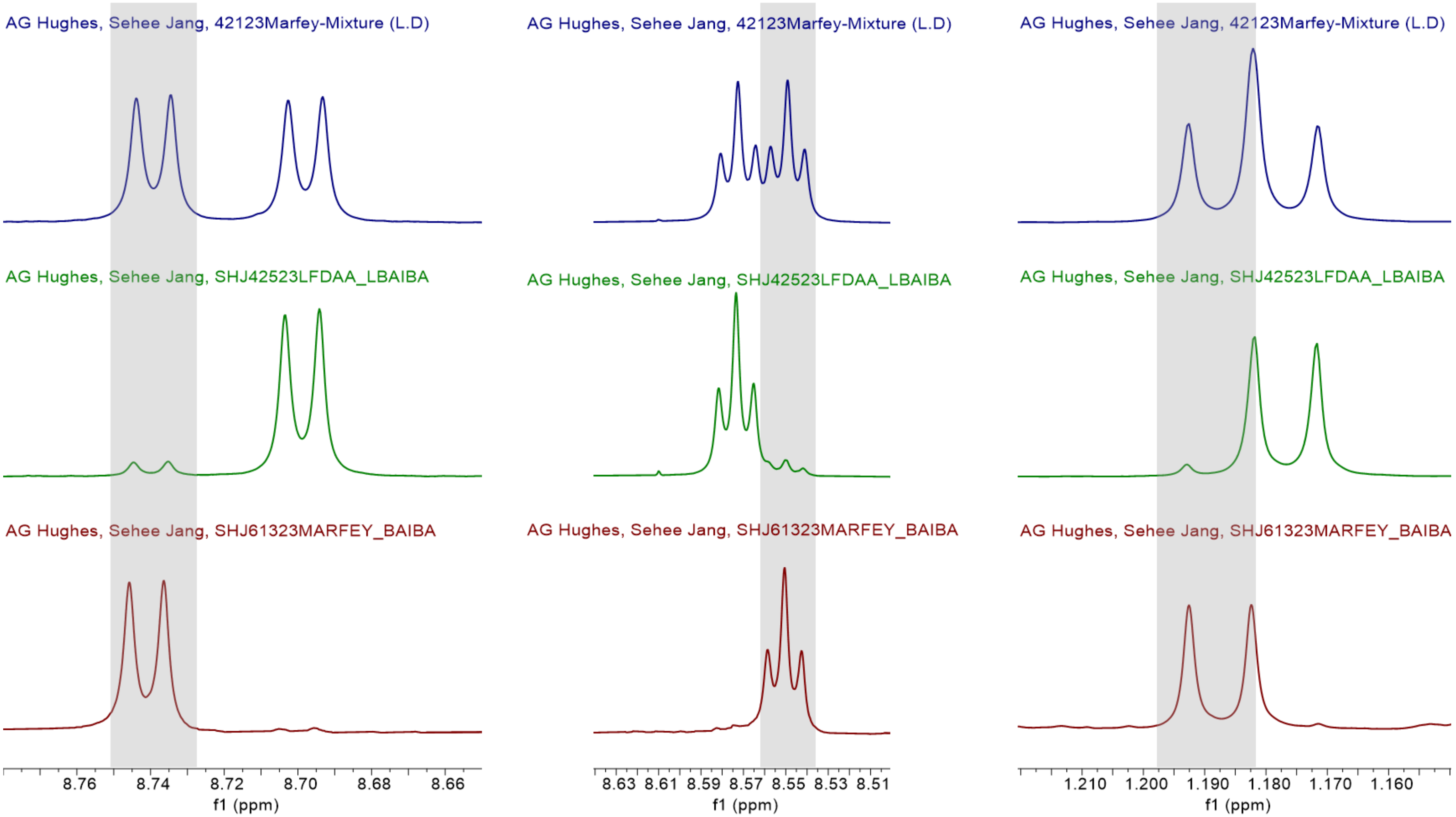

Figure S36 ^{1}H NMR (DMSO-d_6, 700 MHz) of L-FDAA-D/L-BAIBA, L-FDAA-L-BAIBA, and L-FDAA-BAIBA derived from kasichelin C (**3**), which matches L-FDAA-D-BAIBA.

(a)

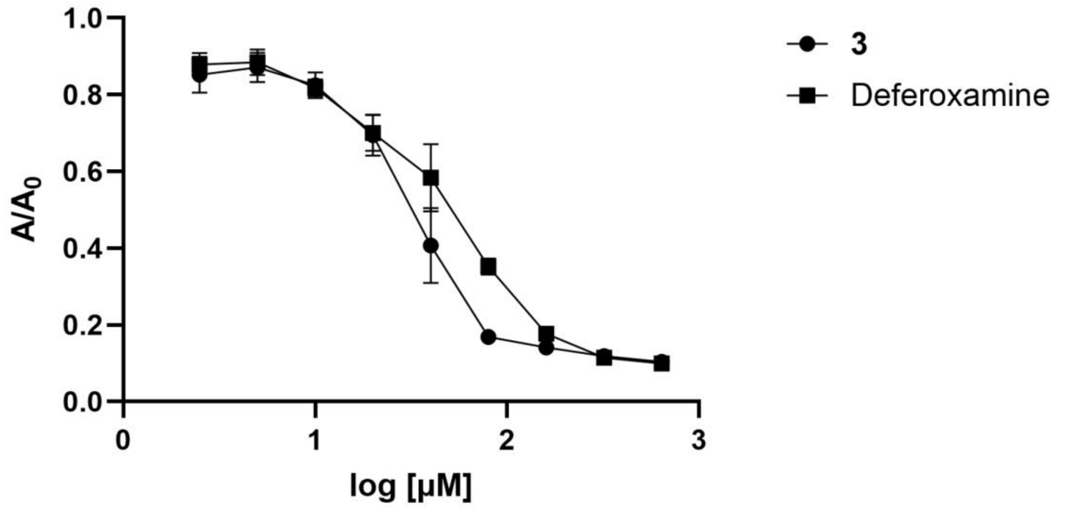

(b)

Compound	EC_{50} (μM)	95 % CI (μM)
**3**	16.54	14.85 to 18.38
Deferoxamine	25.87	22.19 to 30.19

Figure S37 (a) Dose−response curves for the iron binding activity of kasichelin C (**3**) and deferoxamine (b) EC_{50} values and 95% confidence intervals.

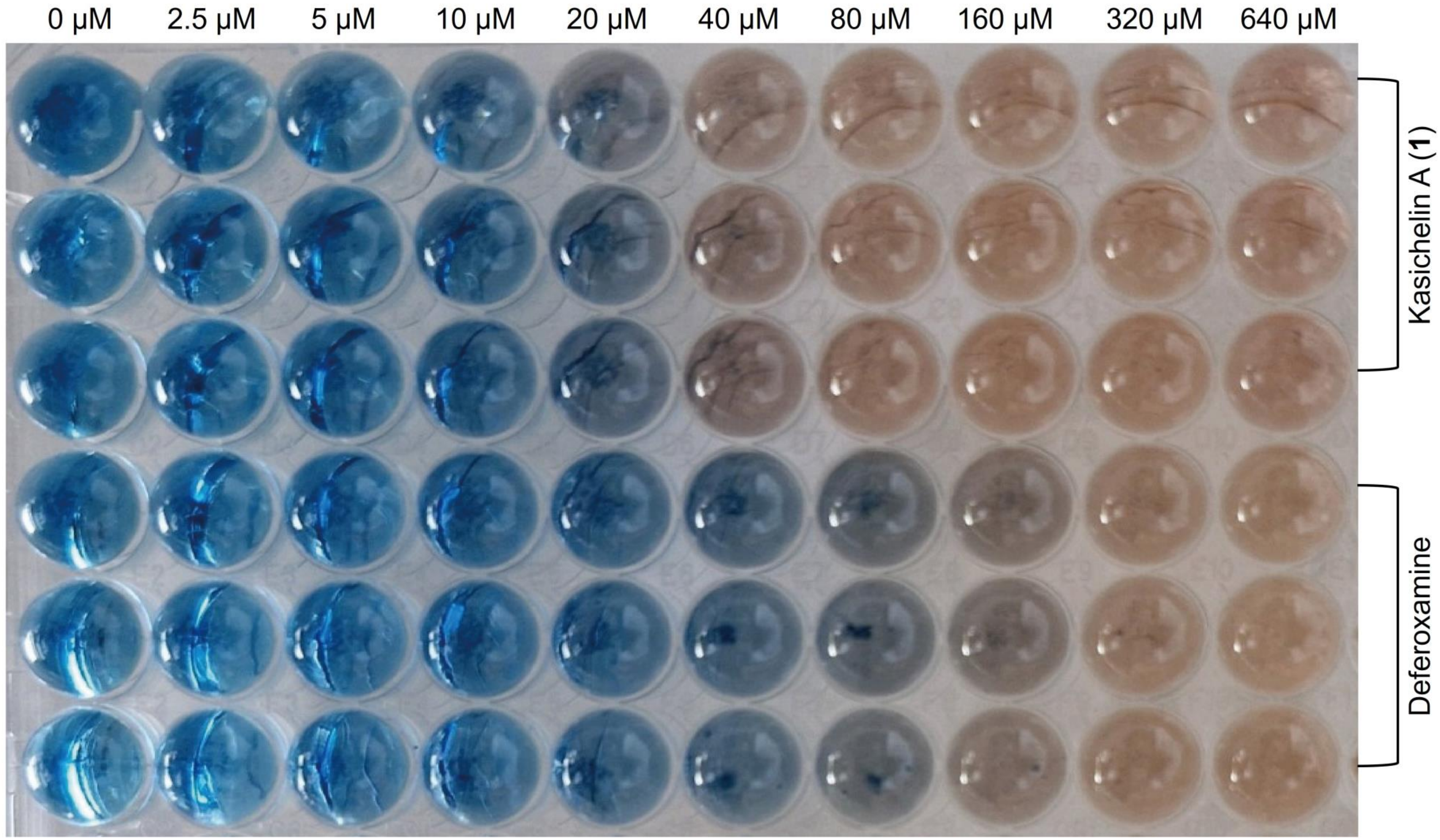

Figure S38 Chrome Azurol S (CAS) assay of kasichelin A (**1**)

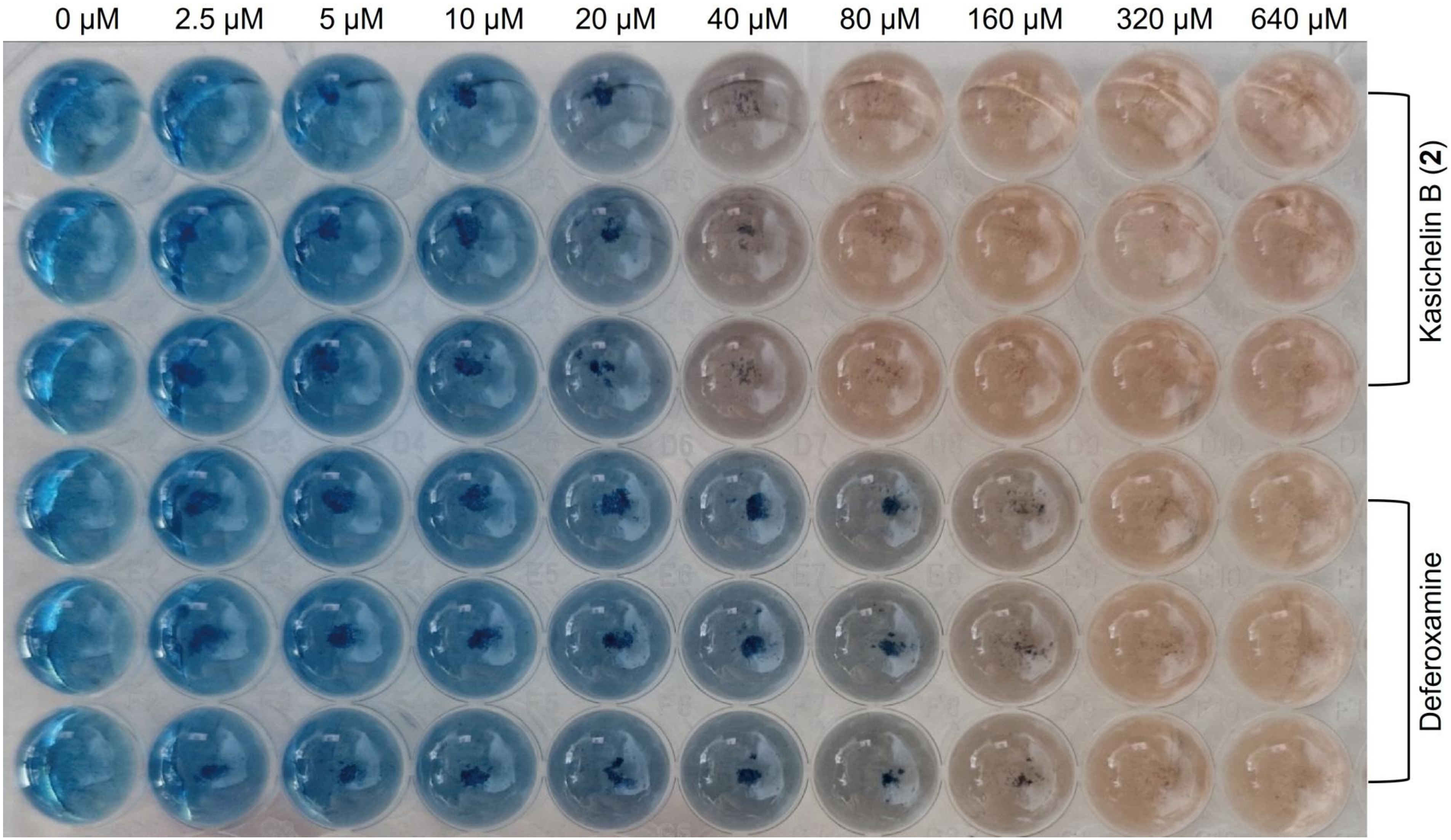

Figure S39 Chrome Azurol S (CAS) assay of kasichelin B (**2**)

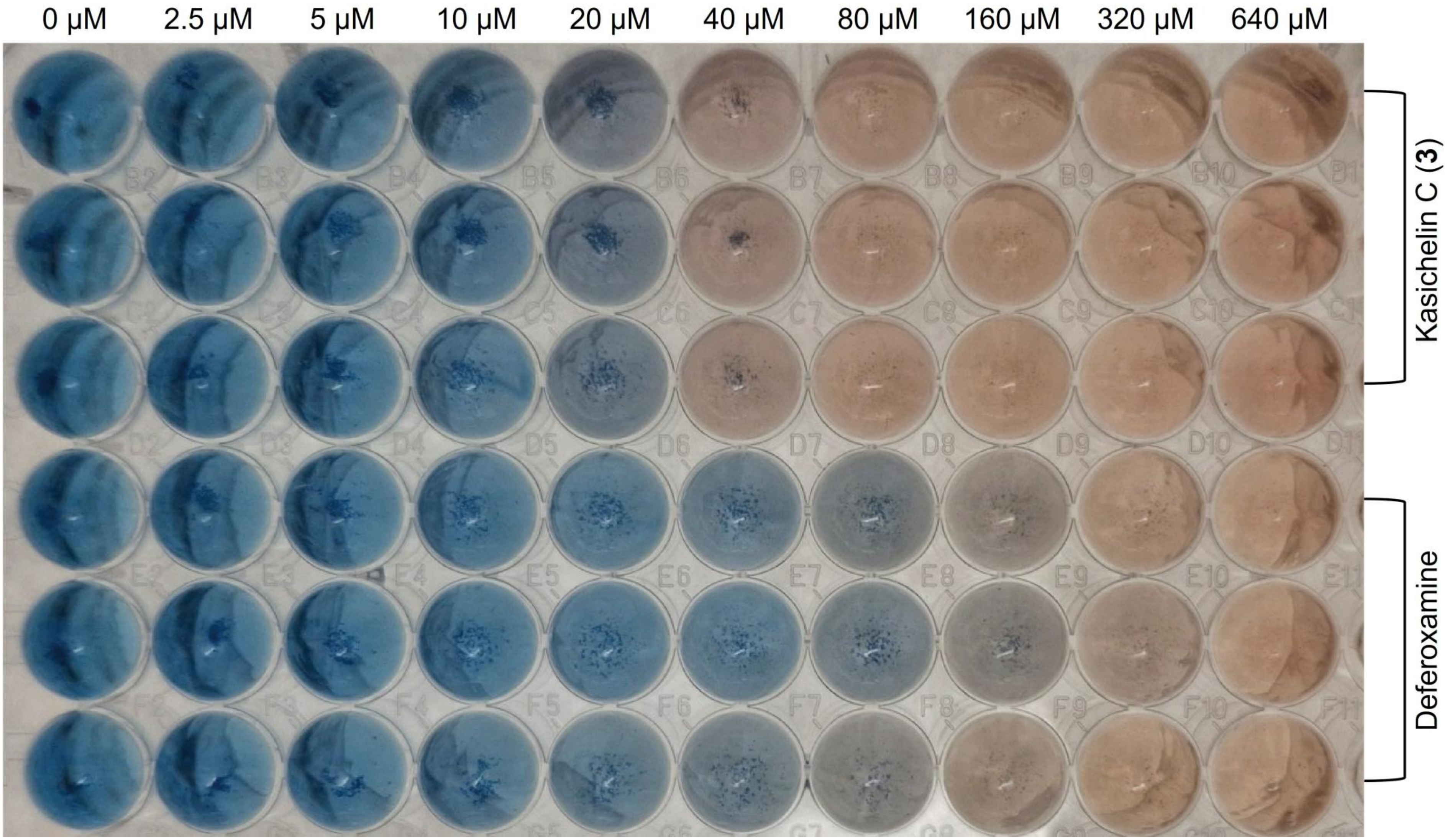

Figure S40 Chrome Azurol S (CAS) assay of kasichelin C (**3**)

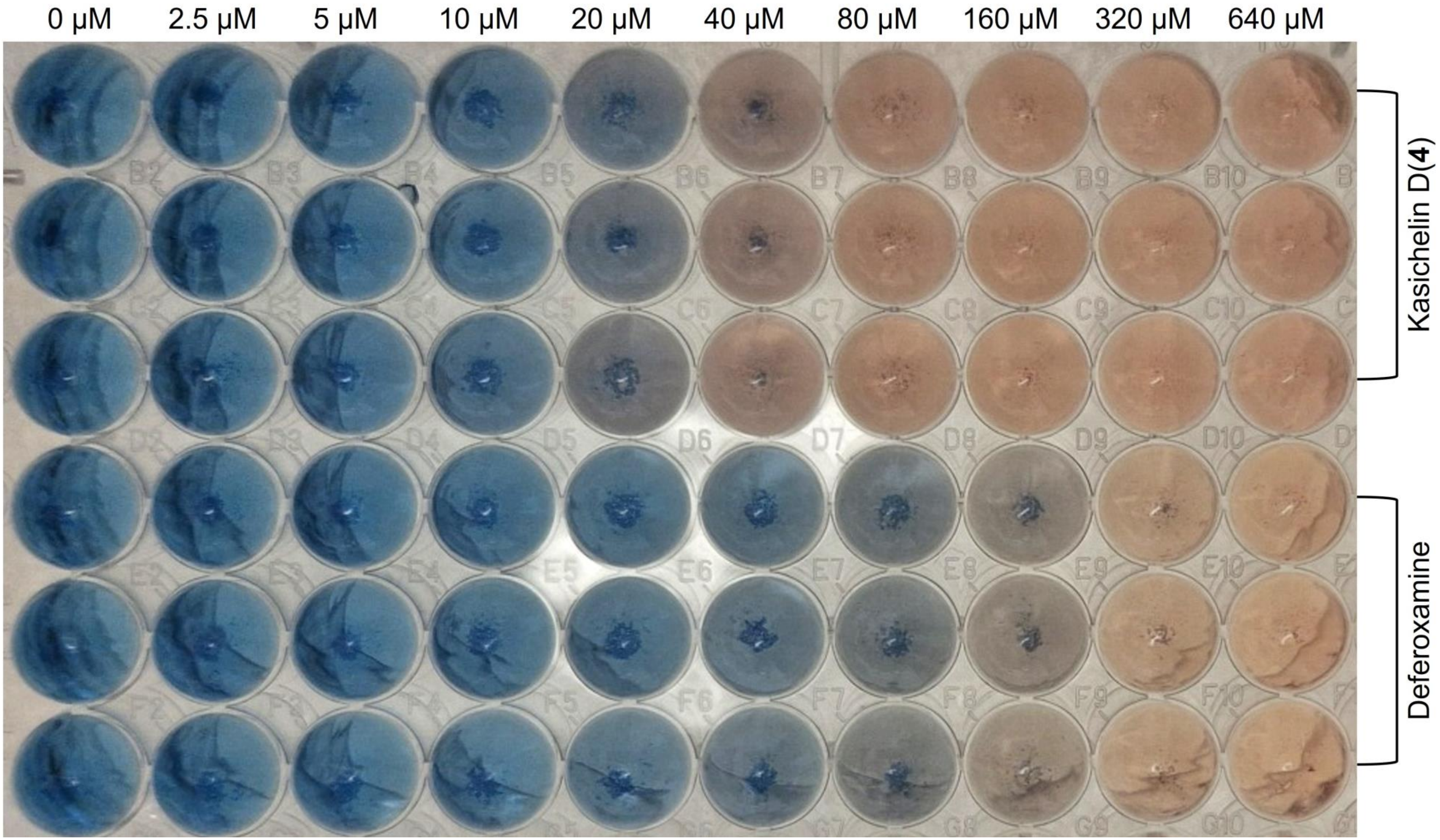

Figure S41 Chrome Azurol S (CAS) assay of kasichelin D (**4**)

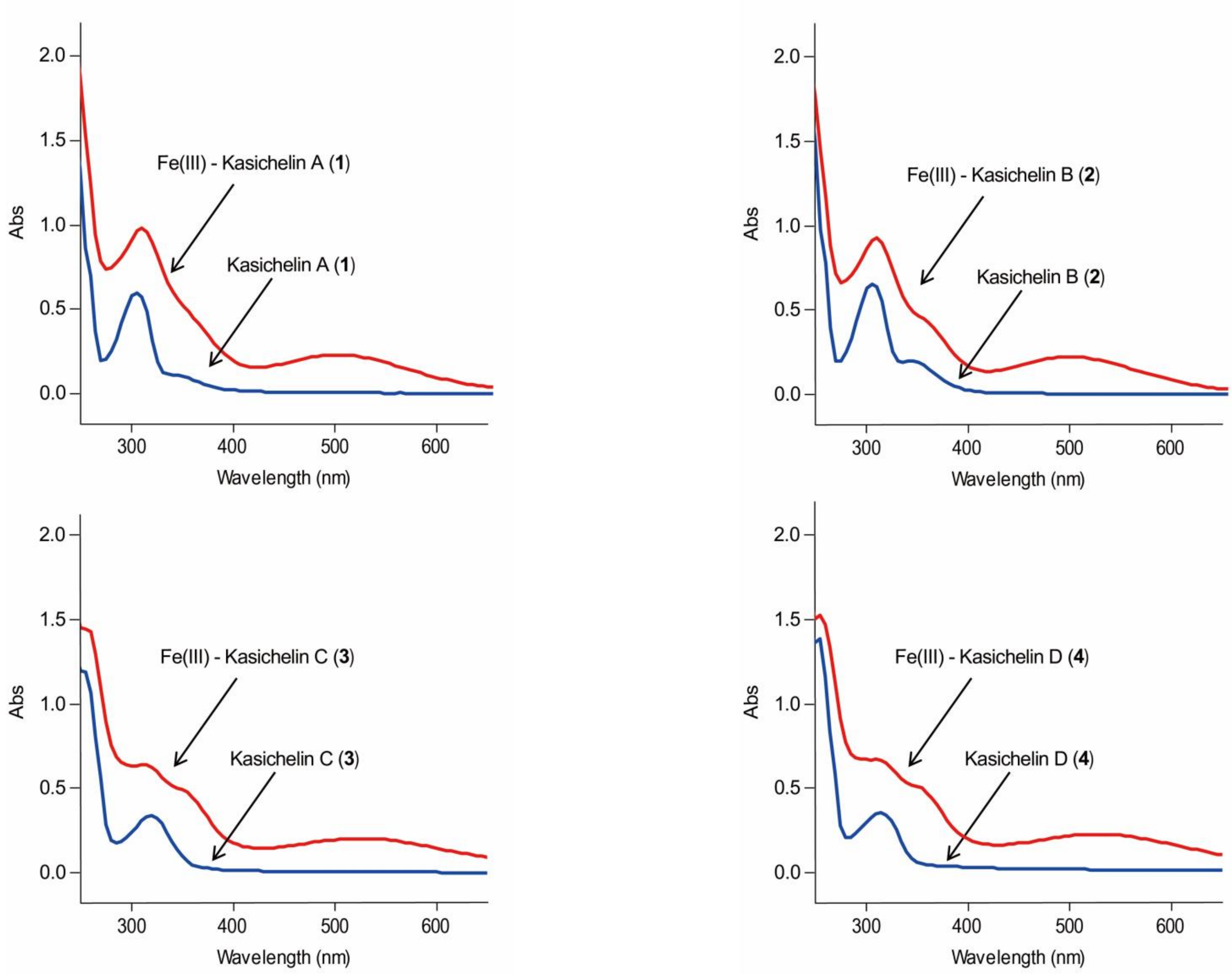

Figure S42 UV-visible spectra of kasichelins A-D (**1**-**4**) and Fe(III)-kasichelin A-D complexes

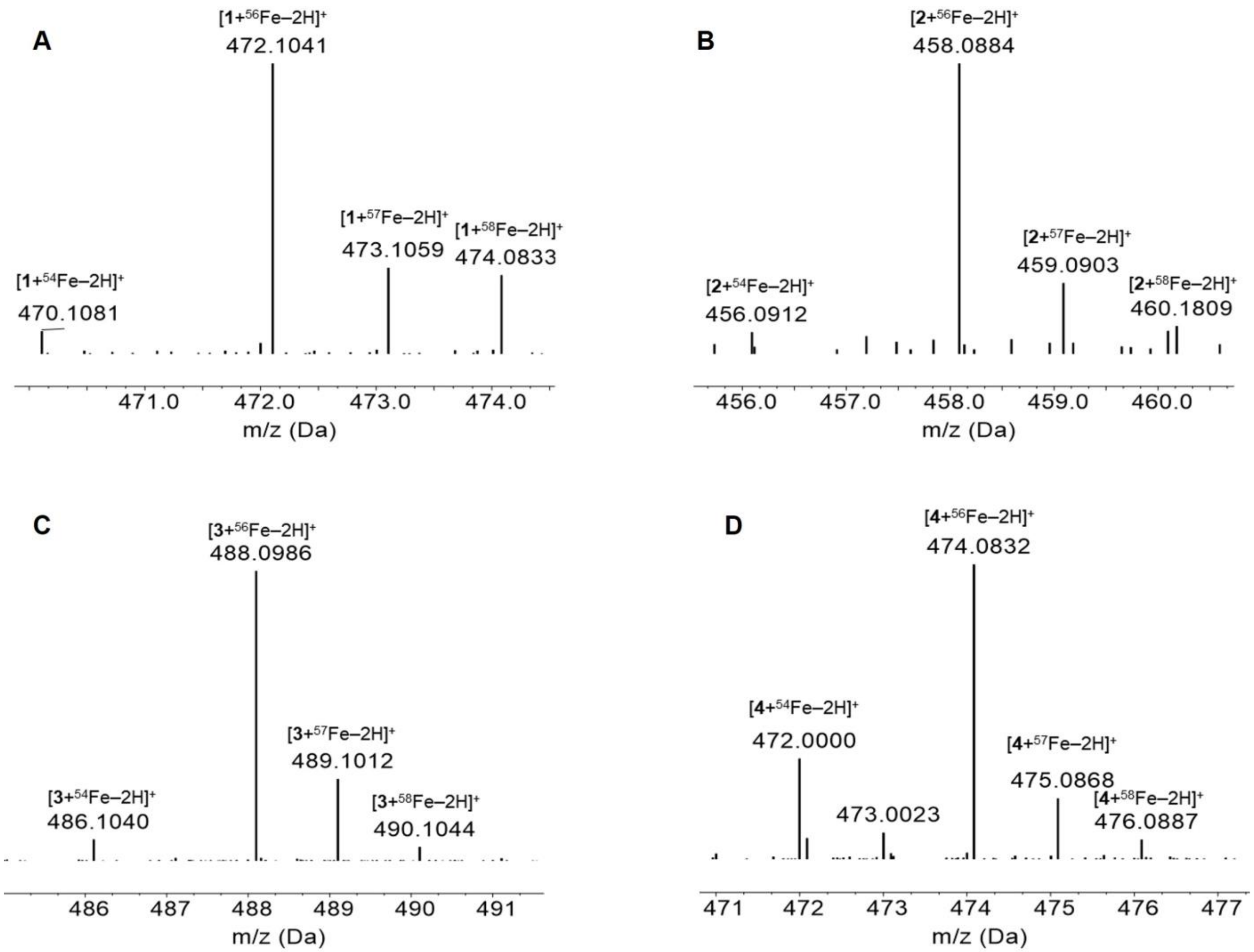

Figure S43 MS spectra of Fe(III)-kasichelin A-D complexes

Table S2. ^{1}H and ^{13}C NMR data for kasichelin C (**3**) with Ga(III) and $^{13}C_3$-labeled compound

M3 = 8 mg, DMSO-d_6 700 MHz, +HCl
M4 = 8 mg, DMSO-d_6 700 MHz, +Ga^{3+}
M5 = 5.5 mg, DMSO-d_6 700/600 (udeft) MHz, $^{13}C_3$-glycerin
M6 = 5.5 mg, CD_6OD 600 MHz (udeft), $^{13}C_3$-glycerin

kasichelin C (**3**)

	δ_C		δ_C (mult., J in Hz)				δ_H (mult., J in Hz)			
position	M3(HCl)	M4 (Ga^{3+})	M5 (700)	M5 (600udeft)	M6	type	M3 (HCl)	M4 (Ga^{3+})	M5 (700)	M6
**1**	164.94	164.99	164.93	164.93 (d, 41.21)	167.31	C	--	--	--	--
**2**	49.52	49.56	49.45	49.45	51.35	CH	4.25 (ddd, 5.25, 8.34, 10.59)	4.25 (m)	4.32 (td, 5.23, 8.46, 10.62)	4.46 (m)
**2-NH**	--	--	--	--	--	NH	8.10 (d, 8.35)	8.11 (d, 8.37)	8.17 (d, 8.53)	--
**3a**<br>**3b**	27.41	27.45	27.46	27.44	28.56	CH2	1.65 (m)<br>1.86 (m)	1.65 (m)<br>1.82 (m)	1.65 (m)<br>1.87 (m)	1.78 (m)<br>2.03 (m)
**4**	20.33	20.36	20.32	20.31	21.81 (d, 33.22)	CH2	1.86 (m)	1.86 (m)	1.87 (m)	2.03 (m)
**5**	51.19	51.23	51.18 (s/d, 33.93)	51.17 (s/d, 32.97)	52.45 (d, 44.46)	CH2	3.44 (d, 4.54)	3.45 (m)	3.46 (dd, 4.64, 9.42)	3.61 (m)
**5-NOH**	--	--	--	--	--	NOH	9.62 (br. s.)	*vanished*	--	--
**6**	173.98	174.01	173.89	173.88	177.31 (d, 37.42)	C	--	--	--	--
**7**	n.d.	39.59 (*)	39.4 (*)	39.28 (*)	41.73 (s/d, 41.69)	CH	overlap (2.50)	2.50 (m, ~)	2.55 (q, 6.98)	2.63 (m)
**7''**	15.36	15.40	15.26 (d, 37.12)	15.23 (s/d, 38.41)	15.49 (d, 45.17)	CH3	0.98 (dd, 2.06, 6.91)	0.98 (d, 6.55)	0.98 (d, 6.88)	1.12 (d, 6.99)
**8a**<br>**8b**	41.93	41.95	42.09	42.08 (s/d, 34.97)	43.72 (s/d, 41.56)	CH2	3.16 (td, 2.93, 6.56, 6.17)	3.16 (t, 6.63)	3.16 (m)<br>3.22 (dt, 6.79, 6.79, 13.33)	3.33 (m)<br>3.46 (dd, 5.12, 13.39)
**8-NH**	--	--	--	--	--	NH	7.95 (t, 5.97)	7.94 (t, 5.86)	8.29 (t, 5.98))	--
**9**	169.94	169.95	169.61	169.60	173.10	C	--	--	--	--
**10**	59.05	59.03	73.50	73.50	75.72	CH	4.37 (dd, 8.02, 4.40)	4.37 (dd, 7.92, 4.27)	4.48 (d, 6.88)	4.49 (m)
**10-NH**	--	--	--	--	--	NH	8.61 (d, 7.87)	8.52 (d, 8.16)	--	--
**11**	66.50	66.54	78.83	78.82	80.58	CH	4.08 (m)	4.08 (m)	4.88 (p, 6.39)	4.90 (m)
**12**	20.24	20.28	20.51	20.50	21.26	CH3	1.08 (d, 6.29)	1.08 (d, 6.16)	1.44 (d, 6.30)	1.53 (d, 6.34)
**13**	167.94	167.94	165.70 (s/d, 70.54)	165.69 (s/d, 70.97)	168.25 (s/d, 71.53)	C	--	--	--	--
**14**	116.74	116.80	110.28	110.27 [110.36 (dd, 29.10, 70.78)]	111.85	C	--	--	--	--
**15**	148.05	147.81	148.39 (d, 35.24)	148.27 (t, 35.04)	149.56 (t, 35.09 o. d, 70.1)	C	--	--	--	--
**15-OH**	--	--	--	--	--	OH	11.80 (s)	*vanished*	11.79 (s)	--
**16**	146.08	146.02	145.78	145.77	146.84	C	--	--	--	--
**16-OH**	--	--	--	--	--	OH	9.62 (s)	*vanished*	10.45 (d, 7.23)	--
**17**	118.42	118.61	119.39 (s/d, 52.72)	119.38 (s/d, 52.48)	119.98 (m)	CH	6.93 (dd, 1.54, 7.76)	6.93 (dd, 1.48, 7.69)	6.96 (dd, 1.73, 7.79)	6.95 (dd, 1.54, 7.87)
**18**	118.08	118.35	118.63 (d, 57.36)	118.59 (t, 53.77)/118.62	119.98 (m)	CH	6.70 (t, 7.91, 7.91)	6.71 (t, 7.88)	6.73 (t, 7.87)	6.75 (t, 7.95)
**19**	118.90	118.91	117.83 (d, 30.95)	117.82 (d, 54.62)	119.98 (m)	CH	7.38 (dd, 1.58, 8.05)	7.37 (dd, 1.68, 8.10)	7.06 (dd, 1.63, 7.95)	7.17 (dd, 1.44, 7.93)

* by HSQC, ~: by HMBC

CrpDA1 (Cryptophycin BGC):	G	D	A	V	F	S	L	A	D	K
KasEA1 (Kasichelin BGC):	L	D	G	T	I	S	L	A	D	K
SurCA1 (Surugamide BGC):	L	D	T	T	V	S	L	A	D	K
CdeIA1 (Cadaside BGC):	L	D	T	T	V	S	L	G	D	K
KasGA1 (Kasichelin BGC):	D	A	Q	E	G	G	F	V	D	K
KasHA1 (Kasichelin BGC):	D	M	F	N	L	G	V	L	W	K

Figure S44 Analysis of the A domains predicted from the kasichelin biosynthetic gene cluster (BGC)

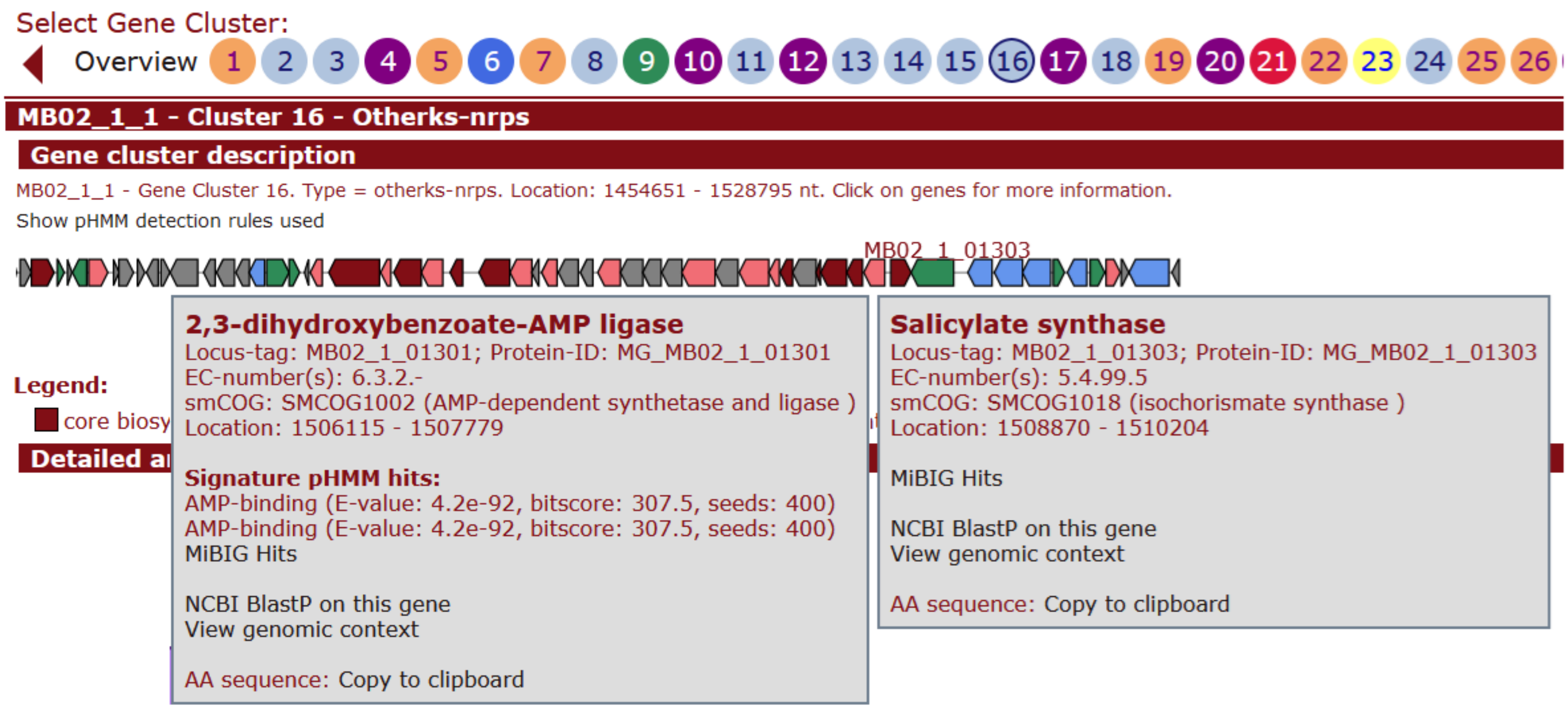

Figure S45 Genetic organization of cluster 16 from *Streptomyces* sp. K17/9

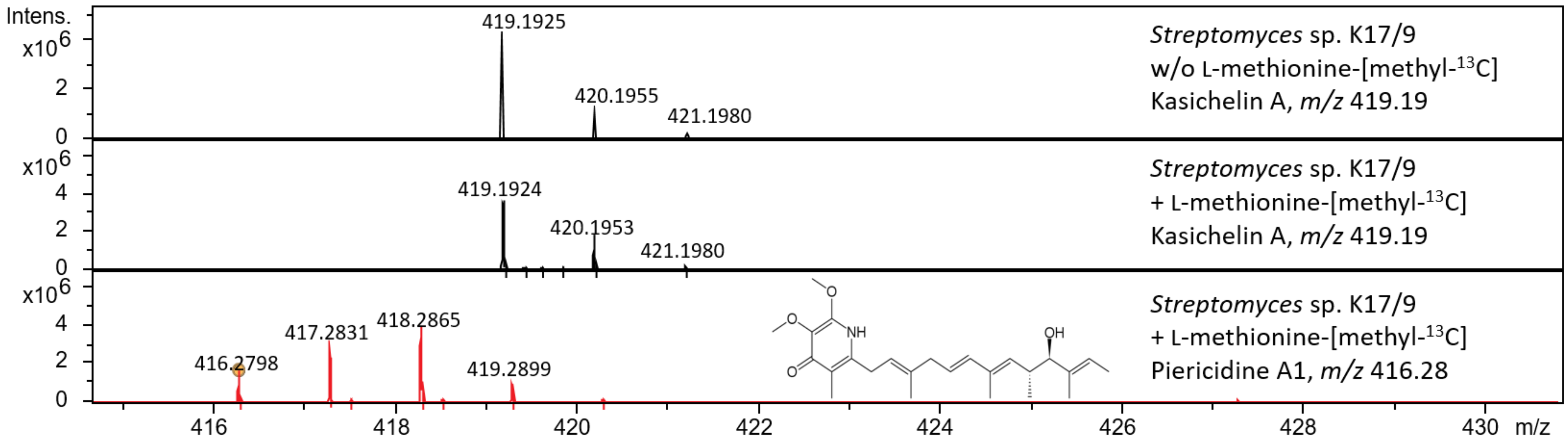

Figure S46 LC-MS analysis of *Streptomyces* sp. K17/9 cultures supplemented with L-methionine-[methyl-^{13}C]
